# Multi-center evaluation of the Selux next-generation phenotyping system for gram-negative direct-from-positive blood culture antimicrobial susceptibility testing

**DOI:** 10.1128/jcm.01819-24

**Published:** 2025-03-31

**Authors:** Vincent Streva, Joseph Gajewski, Jacqueline Pento, Alamelu Chandrasekaran, Matt Green, Jill Lindley, Micahel Huband, Asmae El ganbour, Kristen Roberts, Kelly Flentie, Jingzi Sherman, Eric Stern, Gregory J. Berry

**Affiliations:** 1 Northwell Heath, New York, New York, USA; 2 Element, Acton, Massachusetts, USA; 3 The Center for Advanced Laboratory Medicine, Department of Pathology and Cell Biology, Columbia University Irving Medical Center 21611, New York, New York, USA; 4 Element Iowa City (JMI Laboratories), North Liberty, Iowa, USA; 5 Selux Diagnostics, Charlestown, Massachusetts, USA; Endeavor Health, Evanston, Illinois, USA

**Keywords:** rapid diagnostics, multi-drug resistance, antimicrobial susceptibility test, blood culture

## Abstract

Accurate and rapid antimicrobial susceptibility test (AST) results from positive blood cultures are crucial for patient care and combatting antimicrobial resistance. Although recent advancements in rapid direct-from-positive blood culture (PBC) identification platforms have enabled the provision of species-level identification and some resistance marker information within hours after blood culture positivity, AST results required for clinical decision-making often require 48 h after blood culture positivity. This study evaluated the Selux next-generation phenotyping system, including an automated PBC Separator and the Selux AST system in a multicenter clinical trial for their ability to perform AST directly from PBCs for gram-negative bacilli. The PBC separator produces McFarland equivalent inocula from positive blood cultures within 1 h, facilitating direct processing on the Selux AST system. The study evaluated 162 fresh clinical PBC samples, 307 seeded clinical samples, and 87 seeded challenge samples across 4 sites for each of the 17 antimicrobials included in the panel. The results demonstrate that the Selux system’s clinical performance, reproducibility, and analytical performances are consistent when using positive blood cultures held for up to 16 h after positivity on the BACTEC and BacT/ALERT 3D and BacT/ALERT VIRTUO blood culture systems, including all major BACTEC and BacT/ALERT blood culture bottle types. These findings suggest that the PBC Separator with the Selux AST system is a valuable addition to the arsenal of tools available for rapid sepsis diagnosis and management.

**IMPORTANCE:**

Technologies that consistently and substantially shorten the time between blood bottle positivity, organism identification, and complete AST results are crucial for ensuring that antimicrobial therapy can be tailored. The Selux PBC Separator and the Selux AST system perform rapid AST directly from positive blood culture bottles. This substantially shortens the gap between obtaining a positive blood bottle and organism identification and the availability of a fully actionable AST result.

## INTRODUCTION

Overuse of broad-spectrum antimicrobials results in poor patient outcomes (1, 2) and is partly responsible for the current antimicrobial resistance crisis (3, 4). A major driver of antimicrobial overuse is the time required for antimicrobial susceptibility test (AST) results to become available (5–9). Although bacterial identification (ID) often occurs within a day of sample collection owing to recent technological advances in multiplex molecular and mass spectrometry (5, 6), AST results for blood cultures often require 3–3.5 days from the time when the samples were received at the laboratory to the time the AST result is available (5–9). In clinical laboratories in the United States, much of the delay in time to AST results is due to limitations of current automated AST systems, namely, their inability to perform AST directly from positive blood culture (PBC) samples or to perform accurate AST within a typical 8 h shift (6, 10, 11). Additionally, staffing struggles in clinical laboratories worsen the delay in obtaining rapid AST results. Furthermore, the cost of purchasing and implementing advanced AST systems presents a barrier for many hospitals, which limits the widespread adoption of these technologies. Despite these challenges, the benefits of advanced AST systems in improving patient outcomes and reducing healthcare costs make it worthwhile for hospitals to invest in these technologies, despite the financial commitment.

The Selux AST system gained the Food and Drug Administration (FDA) clearance for isolated colonies in 2023 (12). This system utilizes complementary viability and surface area fluorescence assays (13) to provide rapid AST results across broad antimicrobial menus, including newly approved drugs (14). The PBC Separator with the Selux AST system possesses several notable features, such as the ability to rapidly process positive blood culture samples from nearly all available blood culture systems and bottle types and provide results for a comprehensive antimicrobial menu, including drugs used for escalation and de-escalation, within 6–8 h of blood culture positivity. The 384-well consumable used by the Selux AST system also accommodates broad dilution ranges that align with the FDA Susceptibility Test Interpretive Criteria (STIC) website (15) and CLSI M100 34th edition (16) breakpoints (17).

This study evaluated the Selux direct-from-positive-blood-culture workflow, which includes a PBC Separator sample preparation device that automatically prepares tuned McFarland equivalent inocula from positive blood culture bottles for use with the Selux AST system. A multi-center evaluation was performed at four study sites, which tested samples on the PBC Separator with the Selux AST system, and one reference laboratory, which was blinded to the clinical site results and performed broth microdilution (BMD) reference method testing from isolated colonies (e.g. sub-cultures) of all samples.

## MATERIALS AND METHODS

### PBC Separator with the Selux AST system

The PBC Separator with the Selux AST system comprises three automated instruments: the PBC Separator, the inoculator, and the analyzer. The latter two instruments, which inoculate and process 384-well microplate consumables for AST, are referred to as the Selux AST system and have been described previously (12). The PBC Separator, which automatically prepares Selux AST system-ready inocula from positive blood culture bottles, is described for the first time here.

### PBC Separator

The PBC Separator is an automated sample preparation system that separates intact bacteria from positive blood culture bottles and provides users with tuned McFarland inocula in saline for use with the Selux AST system. The system utilizes saponin, a mammalian cell-specific lytic reagent, and repeated centrifugation-wash cycles to separate bacteria from endogenous and exogenous blood components. The system is designed for compatibility with all models of Becton Dickinson BACTEC and bioMérieux BacT/ALERT 3D and VIRTUO continuous monitoring blood culture systems (18–20). Following confirmation of a positive patient blood sample by an automated blood culture system, an aliquot (9 mL) of the positive blood sample is prepared by the operator for processing by the PBC Separator. While gram status is not required for the initiation of PBC Separator processing, it is necessary for processing the PBC sample on the Selux AST system. The PBC Separator can accommodate up to two samples for automated processing, with an approximate processing time of 45 min for one sample and 1 h for two samples. The instrument transfers the blood to intermediate system modules, isolates the bacteria, and resuspends the bacteria in a saline solution. Upon successful completion of this process, the separated McFarland-equivalent inoculum is transferred by the operator to the Workbench for AST sample preparation within 45 min. The remaining isolated bacteria are contained in a tube in pelleted form for disposal or general laboratory use.

### Study sites

Four sites were used for testing the PBC Separator and Selux AST system: Northwell Health (New York City, New York; Site 1), Selux Diagnostics (Charlestown, MA; Site 2), Element Acton (Acton, MA; Site 3), and New York Presbyterian Hospital-Columbia University Irving Medical Center (New York City, New York; Site 4). All study sites received IRB approval before the study started. Site 1 and site 4 were additionally designated as collection sites for the fresh PBC samples. Collectively, the two New York City-based integrated health network sites comprised fresh clinical samples from more than 50 New York City-area hospitals to ensure that the organisms in this region were sufficiently diverse for the performance evaluation. The prospectively collected fresh PBC samples were tested at site 1 and site 4, respectively, and distributed to site 2 and site 3 for testing. Isolates used for seeded samples were collected from geographically diverse sites across the US: Loyola University (Chicago, IL), the Clinical Microbiology Institute (Tualatin, OR), and annual surveillance studies conducted by International Health Management Associates (IHMA; Schaumberg, IL) and Element Iowa City (JMI Laboratories). All reference method testing was performed at Element Iowa City (a.k.a. JMI Laboratories; North Liberty, IA).

### Clinical study design

The study comprised testing fresh and seeded clinical samples and seeded challenge samples. All samples were gram-negative Enterobacterales (including *C. freundii* complex, *C. koseri*, *E. cloacae* complex, *E. coli*, *K. aerogenes, K. oxytoca, K. pneumoniae*, *M. morganii*, *P. mirabilis*, *P. vulgaris*, and *S. marcescens*), *Acinetobacter baumannii complex*, or *Pseudomonas aeruginosa*. Challenge isolates were specifically selected for resistance and on-scale minimum inhibitory concentrations (MICs) from the Centers for Disease Control and Prevention Antibiotic Resistance bank and Selux banked samples.

Fresh samples were de-identified as remnant positive blood culture samples from the routine clinical workflow. They were loaded into the Selux separator for processing within 16 h of registering positive for bacterial growth. The organism identification of all freshly collected PBC samples obtained at collection sites 1 and 4 was initially performed following routine clinical laboratory procedures and subsequently verified at the reference laboratory (Element Iowa City) utilizing Matrix-Assisted Laser Desorption/Ionization Time-of-Flight Mass Spectrometry (MALDI-TOF MS).

Seeded clinical and challenge samples were prepared by spiking isolates into sterile BACTEC or BacT/ALERT standard aerobic blood culture bottles together with the manufacturer-recommended volume of healthy human donor blood (19, 20). Specifically, 8–10 mL of freshly collected whole blood from healthy donors was drawn into non-charcoal blood culture bottles and provided to testing sites. These bottles were kept at room temperature (RT) until they were seeded with bacteria for clinical and challenge samples within 36 h of collection. The McFarland cell suspension from the F1 plate was serially diluted in saline to spike the blood culture bottles at 10–10,000 CFU/bottle (approximately 1–1,000 CFU/mL) according to the previously reported clinically relevant range for bacteremic patients (18). Seeded bottles were loaded into the clinical site’s continuous monitoring blood culture system and processed according to the manufacturer’s instructions until registering positive for the PBC Separator processing.

All clinical seeded and challenge samples underwent organism identification using the MALDI-TOF MS method at the reference laboratory (Element Iowa City) prior to testing.

All PBC Separator with the Selux AST system testing at each clinical site, including daily QC testing and clinical sample testing, was conducted following the respective IFUs.

Reference method testing on isolated colonies from sub-cultures of each clinical sample was performed by triplicate BMD following the CLSI M100 and M07 guidance documents (16, 21). A single MIC was obtained for each bug-drug combination by taking either the mode of the results or the median if the results spanned three sequential dilutions. If neither of these conditions existed, two additional BMD tests were conducted, and the median of the five results was reported. Additionally, QC testing was performed daily on BMD panels. To minimize bias, readers were blinded to the PBC Separator with Selux AST system results, and panels were randomized each day. Results were not reported for antimicrobial/strain combinations for which two of the BMD results resulted in either a major or very major discrepancy (22) with respect to the third BMD result, and the discrepant result was not in essential agreement (EA) with at least one of the other results. Frozen glycerol stocks containing resuspended isolated colonies from purity plates were shipped from the clinical sites to the reference laboratory for BMD testing of fresh samples.

Testing was conducted from March 2022 through January 2024. 219 fresh clinical positive blood culture samples, 318 seeded clinical samples, and 88 seeded challenge samples were evaluated across the four study sites. A total of 59 (9.4%) samples were excluded, comprising 28 samples (4.5%) due to operational errors (e.g. protocol adherence issues, incorrect ID), 11 samples (1.8%) due to the system not reporting results, 5 samples (0.8%) due to contamination or an absent purity plate, 4 samples (0.7%) due to QC failures, and 4 samples to a software issue at one site. Additionally, four samples required repeat separator processing and three required repeat inoculator processing due to processing issues. No bias was observed in the sample exclusion. Consequently, a total of 162 fresh clinical positive blood culture samples, 307 seeded clinical samples, and 87 seeded challenge samples with valid results were included in the data analysis. Performance was evaluated for the 17 antimicrobials listed in Table 1. Table 1 also lists the reporting range and applied breakpoints for each reporting group. Breakpoints for 510 (k) submission to the FDA are defined by the FDA STIC website (15) and are accurate to February 2024 when FDA clearance was granted. Details on the evaluated organism-antimicrobial combinations are given in Table S1.

**TABLE 1:** Reporting range and breakpoints applied in the evaluation of the PBC Separator and the Selux AST system

Antimicrobial agent	Reporting group	PBC Separator with the Selux AST system reporting range	FDA breakpoints
Amikacin	*A. baumannii* complex	≤0.12 to ≥256 µg/mL	≤16/32/≥64
	*P. aeruginosa*		
	Enterobacterales	≤2 to ≥256 µg/mL	
Amoxicillin-Clavulanate	Enterobacterales	≤2 to ≥128 µg/mL	≤8/16/≥32
Ampicillin	Enterobacterales	≤2 to ≥128 µg/mL	≤8/16/≥32
Ampicillin-Sulbactam	*A. baumannii* complex	≤2 to ≥128 µg/mL	≤8/16/≥32
	Enterobacterales	≤0.5 to ≥128 µg/mL	
Cefazolin	Enterobacterales	≤0.12 to ≥128 µg/mL	≤2/4/≥8
Cefepime	Enterobacterales	≤0.5 to ≥32 µg/mL	≤2/4–8/≥16
	*P. aeruginosa*	≤0.25 to ≥128 µg/mL	≤8/≥16
Ceftazidime	*P. aeruginosa*	≤0.25 to ≥256 µg/mL	≤8/≥16
	Enterobacterales	≤0.25 to ≥64 µg/mL	≤4/8/≥16
Ceftazidime-Avibactam	Enterobacterales	≤0.12 to ≥64 µg/mL	≤8/≥16
	*P. aeruginosa*		
Ceftriaxone	Enterobacterales	≤0.25 to ≥32 µg/mL	≤1/2/≥4
Ciprofloxacin	Enterobacterales	≤0.03 to ≥16 µg/mL	≤0.25/.5/≥1
	*P. aeruginosa*		≤0.5/1/≥2
Ertapenem	Enterobacterales	≤0.03 to ≥16 µg/mL	≤0.5/1/≥2
Gentamicin	Enterobacterales	≤1 to ≥64 µg/mL	≤4/8/≥16
	*P. aeruginosa*	≤0.5 to ≥64 µg/mL	
Imipenem	*A. baumannii* complex	≤0.5 to ≥64 µg/mL	≤2/4/≥8
	Enterobacterales	≤0.25 to ≥16 µg/mL	≤1/2/≥4
Meropenem	*A. baumannii* complex	≤0.12 to ≥64 µg/mL	≤2/4/≥8
	Enterobacterales		≤1/2/≥4
	*P. aeruginosa*		≤2/4/≥8
Minocycline	*A. baumannii* complex	≤0.25 to ≥64 µg/mL	≤4/8/≥16
	Enterobacterales		
Piperacillin-Tazobactam	*A. baumannii* complex	≤4 to ≥512 µg/mL	≤16/32–64/≥128
	Enterobacterales	≤2 to ≥128 µg/mL	≤8/16/≥32
	*P. aeruginosa*	≤0.25 to ≥512 µg/mL	≤16/32–64/≥128
Tobramycin	*P. aeruginosa*	≤0.12 to ≥128 µg/mL	≤4/8/≥16
	Enterobacterales		

### Reproducibility studies

Intra- and inter-site reproducibility studies were performed for at least one representative antimicrobial for each antimicrobial class represented on the 17-antimicrobial menu. A minimum of five strains were tested for each antimicrobial, including a minimum of one strain from each reporting group relevant to the antimicrobial under test. Each strain was tested in triplicate from three different PBC Separator-prepared inoculums derived from seeded blood culture bottles at each of the three clinical sites. There are, thus, a minimum of 45 (5 × 3 × 3) results per antimicrobial per site. In turn, intra-site reproducibility has a minimum of 45 results per antimicrobial, and inter-site reproducibility has a minimum of 135 (45 × 3) results per antimicrobial. Following FDA guidance (22), performance is determined by the number of results for each strain that is within the EA of the modal result for that strain.

### Analytical studies

All analytical studies were conducted using bacteria seeded at previously reported clinically relevant concentrations (10–10,000 CFU/bottle) (18) into blood culture bottles containing the manufacturer-recommended volume (Table 2) of human blood that had been collected from healthy donors no more than 36 h prior to use and stored at RT during this time. Each sample was incubated in a BacT/ALERT or BACTEC continuous monitoring blood culture system. After registering positive for growth, each sample began processing on the PBC Separator within 16 h. Each result was derived from a separate PBC Separator-produced McFarland inoculum prepared from a separate blood culture bottle. For all analytical studies other than the positive blood culture sample stability study, Selux results are compared with reference BMD performed by Element Iowa City, and acceptable performance is defined as an EA of >89.9% between the methods. For the positive blood culture sample stability study, Selux results at subsequent time points were compared with results at the initial time point, T0.

**TABLE 2:** Blood culture bottle types, manufacturer-recommended fill volumes, and corresponding blood culture systems.

Bottle type	Direct fill blood volume	Blood culture system compatibility
BACTEC Plus Aerobic	8–10 mL	BD BACTEC
BACTEC Plus Anaerobic	8–10 mL	BD BACTEC
BACTEC Standard Aerobic	8–10 mL	BD BACTEC
BACTEC Standard Anaerobic	5–7 mL	BD BACTEC
BACTEC Lytic Anaerobic	8–10 mL	BD BACTEC
BACTEC Peds Plus	1–3 mL	BD BACTEC
BacT/ALERT FA Plus	8–10 mL	bioMérieux BacT/ALERT 3D, VIRTUO
BacT/ALERT FN Plus	8–10 mL	bioMérieux BacT/ALERT 3D, VIRTUO
BacT/ALERT SA	8–10 mL	bioMérieux BacT/ALERT 3D, VIRTUO
BacT/ALERT SN	8–10 mL	bioMérieux BacT/ALERT 3D, VIRTUO
BacT/ALERT PF Plus	1–3 mL	bioMérieux BacT/ALERT 3D, VIRTUO

### Seeded bacterial concentration in the blood bottle study

This study evaluated the performance of the Selux system across the bacterial burden that can be found in the blood of bacteremic patients (18). This comprised a 3-log range, including microorganism concentrations of 10, 100, 1,000, and 10,000 CFU/bottle, equivalent to approximately 1, 10, 100, and 1,000 CFU/mL in blood. It is important to note that the final concentration of bacteria inoculated into each antimicrobial dilution well by the Selux inoculator, approximately 5 × 10^5^ CFU/mL, is independent of the concentration of bacteria in the blood culture bottle. A minimum of one sample from each reporting group for each drug was tested for each concentration.

### Positive blood culture sample stability study

To test the stability of positive blood culture specimens prior to beginning processing with the PBC Separator, seeded blood culture bottles were tested at 0, 4, 8, 12, 16, or 18 h at RT after registering positive and being removed from a continuous monitoring blood culture system. The study design followed CLSI EP25 guidance (23) such that the last time point (18 h) is >10% later than the time point (16 h) to be claimed for stability. Positive blood culture samples were removed from the blood culture system immediately upon registering positive growth and were stored at RT until PBC Separator processing began. At each time point, a minimum of two results from each reporting group for each drug was collected.

### Blood culture bottle compatibility study

The 11 BD BACTEC and bioMérieux BacT/ALERT blood culture bottles listed in Table 2 were evaluated. Four species were tested for each bottle type, with a minimum of two results per species. Aerobic bottles were seeded with *A. baumannii* complex, *E. coli*, *K. pneumoniae*, or *P. aeruginosa*, and anaerobic bottles were seeded with *E. coli*, *K. pneumoniae*, *P. mirabilis*, or *M. morganii*.

### Blood culture system compatibility study

Samples were incubated in either a BacT/ALERT VIRTUO or a BACTEC 9050. A minimum of eight results from each reporting group for each drug were collected for each blood culture system. The number of samples for which AST results could be determined was also collected.

### Interfering substance studies

Seven endogenous and four exogenous interfering substances were evaluated using non-resin bottles. The endogenous interfering substances evaluated were red blood cells, white blood cells, platelets, conjugated bilirubin, unconjugated bilirubin, triglycerides, and gamma globulins. The concentrations tested are listed in Table S2. The exogenous interfering substances evaluated were cefpodoxime, ciprofloxacin, penicillin, and gentamicin. The concentrations tested are listed in Table S3. Interferents were tested at or above the concentrations observed clinically. The concentrations for the endogenous and exogenous interferents were determined according to published clinically relevant levels (24–26). Each potentially interfering substance was inoculated into the healthy donor blood-filled blood culture bottle directly prior to bacterial seeding. A minimum of three results from each reporting group for each drug were collected for each potential interferent.

## RESULTS

The data from the bacterial seeding concentration study, provided in Table S4, demonstrate >95% overall EA for the PBC Separator with the Selux AST system compared with reference BMD regardless of bacteria seeding concentration throughout the 10–10,000 CFU/bottle range. The data indicated no overall effect of seeded bacteria concentration on AST results with the PBC Separator with the Selux AST system. In two cases, ciprofloxacin at bacterial seeding concentrations of 10 CFU/bottle and 1,000 CFU/bottle performance was <90% EA due to a single error in each case. Seeding was, thus, performed with a 10–10,000 CFU/bottle range for all seeded clinical and challenge samples.

The data from the positive blood culture sample stability study are presented in Table S5. The results suggest no impact of positive blood culture sample storage up to 16 h on AST results with the PBC Separator with the Selux AST system.

The data for the blood culture bottle compatibility study are provided in Table S6A and B. These data demonstrate the independence of the PBC Separator with the Selux AST system across the 11 primary blood culture bottle types (6 BACTEC and 5 BacT/ALERT), including all aerobic and anaerobic, resin-containing and resin-free, and pediatric and lytic bottles listed in Table 2. The overall performance demonstrated 95% EA in comparison to the BMD reference method, with the exception of minocycline with BD Plus Aerobic bottles.

The PBC Separator with the Selux AST system performance was also evaluated for a representative first- and second-generation continuous monitoring blood culture system, the BACTEC 9050 and the BacT/ALERT VIRTUO, respectively. The data for this study are provided in Table S7 and show no performance impact either in terms of EA or yield for the BacT/ALERT VIRTUO compared with BACTEC 9050. These data demonstrate the independence of the PBC Separator with the Selux AST system on the continuous monitoring blood culture system.

To evaluate the system for a variety of septic patients, the PBC Separator with the Selux AST system was evaluated in the presence of seven potential endogenous interfering substances that may be present at elevated ranges during sepsis. Elevated levels of cells and cell fragments—red blood cells, white blood cells, and platelets—and molecules—conjugated bilirubin, unconjugated bilirubin, triglycerides, and gamma globulins—were evaluated at the extent of their reported ranges (Table S2). The data for this study are provided in Table S8. These data show no overall performance impact of these common endogenous interfering substances on the system, with the exception of <89.9% EA observed for piperacillin-tazobactam for some endogenous interfering substances. The potential impact of orally available antimicrobials was also evaluated, and study results are given in Table S9. These data demonstrate no impact on the performance of AST results.

Inter-site reproducibility was assessed by processing at least five strains for each antimicrobial agent three times from separate PBC Separator-prepared inoculums on three separate days at each of three clinical sites for a representative antimicrobial of each class determined by the FDA. Intra-site reproducibility, defined as the performance at a single site, site 2, was performed as a part of the inter-site study. Results are given in Table 3 following FDA guidance (22), whereby the modal MIC result for each sample is determined, and all results for that sample that lie within one doubling dilution of that MIC are tallied, whereas those outside this range are not. The data in Table 3 meet the >95% FDA criteria for reproducibility, demonstrating PBC Separator with the Selux AST system performance independent of operator and site.

**TABLE 3:** Results of intra- and inter-site reproducibility studies for the PBC Separator and the Selux AST system^*a*^

Antimicrobial agent	Inter-site reproducibility		Intra-site reproducibility	
	Best-case	Worst-case	Best-case	Worst-case
Ampicillin	99.3% (134/135)	99.3% (134/135)	100% (45/45)	100% (45/45)
Ampicillin-sulbactam	100% (162/162)	100% (162/162)	100% (54/54)	100% (54/54)
Amoxicillin-clavulanate	100% (135/135)	100% (135/135)	100% (45/45)	100% (45/45)
Cefazolin	100% (135/135)	100% (135/135)	100% (45/45)	100% (45/45)
Ceftazidime-avibactam	100% (135/135)	100% (135/135)	100% (45/45)	100% (45/45)
Ciprofloxacin	100% (162/162)	100% (162/162)	100% (54/54)	100% (54/54)
Gentamicin	98.8% (160/162)	98.8% (160/162)	100% (54/54)	100% (54/54)
Meropenem	95.8% (181/189)	95.8% (181/189)	98.4% (62/63)	98.4% (62/63)
Minocycline	96.3% (156/162)	96.3% (156/162)	98.1% (53/54)	98.1% (53/54)

^*a*^ There was no difference between best- and worst-case performance for PBC samples. Best-case performance is defined as all results within EA of the modal result for that sample, whether or not the result is on-scale (defined as growth in at least one well of the dilution series and no growth in at least one well of the dilution series). Best-case performance must be ≥95%. Worst-case performance does not count off-scale results (no growth in any dilution or growth in all dilutions) as being in EA of the modal result. Best-case performance must be ≥89%.

The clinical study data comprise the clinical and challenge samples. These results are given in Table 4 for the 17 antimicrobials evaluated in the study. Results were evaluated for EA, categorical agreement (CA), very major errors (VMJ), and major errors (MAJ) according to the FDA STIC website breakpoints (15) at the time of 510 (k) clearance, February 2024. Results for all organisms enrolled in the study with recognized FDA breakpoints—which are restricted to organisms listed in the reference listed drug label—are included for each drug. The data in Table 4 demonstrate the PBC Separator with the Selux AST system meets FDA and ISO performance requirements (17, 22) for each drug. The CA of <89.9% for amoxicillin-clavulanate, ampicillin-sulbactam, and minocycline with Enterobacterales and minocycline with *A. baumannii* are deemed acceptable by the FDA following the guidance that <89.9% CA is acceptable provided EA is suitably high (22). The only VMJ in the study, *E. coli* with cefazolin, gave an acceptable VMJ rate of 1.9% for this species-drug combination, below the 2% requirement. The one MAJ error for each of ceftriaxone, cefepime, ciprofloxacin, gentamicin, and piperacillin-tazobactam gave MAJ error rates that meet the 3% requirement for each species-drug combination. The results for *K. oxytoca* and *M. morganii* with ampicillin-sulbactam are suppressed by the system due to low CAs despite EAs of 95.2% and 100%, respectively. Breakpoints and reporting ranges for each combination are provided in Table 1. The resistance mechanisms for the challenge samples that were characterized molecularly are given in Table S10 and the phenotypic AST profiles for each organism are provided in Table S11. Tables S12 and 13 provide a sub-analysis of the performance based on fresh versus contrived samples. The PBC Separator also demonstrated 100% QC performance at each trial site during the study.

**TABLE 4:** Results of clinical performance evaluation for the PBC Separator and the Selux AST system

Antimicrobial agent	Organism group	Total tested	# EA	% EA	Total eval	# Eval in EA	% EA of Eval	# CA	% CA	# R	# VMJ	# MAJ	# MIN
Amikican	*A. baumannii* complex	38	35	92.1	23	20	87	36	94.7	17	0	0	2
	Enterobacterales	216	208	96.3	24	16	66.7	213	98.6	8	0	0	3
	*P. aeruginosa*	44	42	95.5	43	41	95.3	40	90.9	6	0	0	4
Amoxicillin-clavulanate	Enterobacterales	330	328	99.4	222	220	99.1	295	89.4	39	0	0	35
Ampicillin	Enterobacterales	149	148	99.3	4	3	75	148	99.3	96	0	0	1
Ampicillin-sulbactam	*A. baumannii* complex	40	37	92.5	21	18	85.7	38	95	25	0	0	2
	Enterobacterales	352	347	98.6	303	298	98.3	306	86.9	135	0	0	46
Cefazolin	Enterobacterales	207	197	95.2	118	108	91.5	187	90.3	99	1	0	19
Cefepime	Enterobacterales	406	396	97.5	35	25	71.4	390	96.1	61	0	0	16
	*P. aeruginosa*	43	42	97.7	37	36	97.3	42	97.7	11	0	0	0
Ceftazidime	Enterobacterales	217	215	99.1	66	64	97	204	94	70	0	0	13
	*P. aeruginosa*	40	40	100	35	35	100	40	100	9	0	0	0
Ceftazidime-avibactam	Enterobacterales	418	409	97.8	96	87	90.6	418	100	4	0	0	0
	*P. aeruginosa*	43	43	100	39	39	100	42	97.7	9	0	0	0
Ceftriaxone	Enterobacterales	373	370	99.2	18	15	83.3	370	99.2	111	0	1	2
Ciprofloxacin	Enterobacterales	469	461	98.3	82	74	90.2	457	97.4	117	0	1	11
	*P. aeruginosa*	43	43	100	32	32	100	42	97.7	13	0	0	1
Ertapenem	Enterobacterales	412	405	98.3	62	55	88.7	408	99	28	0	0	4
Gentamicin	Enterobacterales	466	459	98.5	33	26	78.8	459	98.5	64	0	1	6
	*P. aeruginosa*	43	42	97.7	31	30	96.8	42	97.7	8	0	0	1
Imipenem	*A. baumannii* complex	39	38	97.4	4	3	75	39	100	26	0	0	0
	Enterobacterales	213	205	96.2	14	6	42.9	209	98.1	20	0	1	3
Meropenem	*A. baumannii* complex	39	37	94.9	14	12	85.7	39	100	27	0	0	0
	Enterobacterales	394	388	98.5	19	13	68.4	390	99	19	0	0	4
	*P. aeruginosa*	43	40	93	28	25	89.3	39	90.7	13	0	0	4
Minocycline	*A. baumannii* complex	39	38	97.4	24	23	95.8	33	84.6	12	0	0	6
	Enterobacterales	218	209	95.9	193	184	95.3	195	89.4	32	0	2	21
Piperacillin-tazobactam	*A. baumannii* complex	39	37	94.9	5	3	60	38	97.4	27	0	0	1
	Enterobacterales	320	313	97.8	36	29	80.6	312	97.5	37	0	1	7
	*P. aeruginosa*	43	42	97.7	40	39	97.5	42	97.7	9	0	0	1
Tobramycin	Enterobacterales	216	207	95.8	200	191	95.5	200	92.6	49	0	0	16
	*P. aeruginosa*	43	41	95.3	37	35	94.6	41	95.3	10	0	0	2

Breakpoints for certain antimicrobial agents with organism group combinations can differ between the Clinical and Laboratory Standards Institute (CLSI) and the FDA. For the antimicrobial-organism combinations evaluated in this study, the breakpoints differ for amikacin, ceftazidime, cefepime, gentamicin, piperacillin-tazobactam, and tobramycin. The performance of these antimicrobials was evaluated using CLSI breakpoints (Table S14). All antimicrobial-organism combinations demonstrated acceptable EA, CA, VMJ, and MAJ, except one VMJ for amikacin with Enterobacterales.

The time-to-results (TTRs) for all clinical and challenge samples were also collected during the study to determine the speed of sample processing. The TTR data are given in Table 5 and are divided into time for PBC Separator operation and time for Selux AST operation. The speed of sample processing with the Selux AST system is unchanged from that of isolated colonies (12). The PBC Separator adds approximately 45 min if one sample is prepared or 1 h if two are prepared simultaneously.

**TABLE 5:** TTR for the PBC Separator and the Selux AST system

Organism	Number of samples	Analyzer TTR^*a*^ (h)		Total TTR,^*a*^ separator start to analyzer end (h)	
		Median	95% CI	Median	95% CI
*Acinetobacter baumannii* complex	40	5.27	(5.17/5.34)	6.54	(6.43/6.70)
*Citrobacter freundii* complex	20	5.15	(5.12/5.25)	6.54	(6.48/6.67)
*Citrobacter koseri*	20	5.16	(5.07/5.26)	6.54	(6.44/6.67)
*Enterobacter cloacae* complex	33	5.21	(5.17/5.26)	6.55	(6.51/6.60)
*Escherichia coli*	157	5.19	(5.16/5.22)	6.51	(6.48/6.56)
*Klebsiella aerogenes*	20	5.18	(5.13/5.27)	6.63	(6.45/6.74)
*Klebsiella oxytoca*	21	5.17	(5.10/5.30)	6.48	(6.39/6.79)
*Klebsiella pneumoniae*	113	5.22	(5.19/5.27)	6.61	(6.57/6.66)
*Morganella morganii*	21	5.18	(5.11/5.31)	6.63	(6.37/6.74)
*Proteus mirabilis*	22	5.16	(5.08/5.25)	6.53	(6.41/6.67)
*Proteus vulgaris*	22	5.15	(5.10/5.21)	6.57	(6.44/6.65)
*Pseudomonas aeruginosa*	44	6.66	(6.59/6.75)	7.97	(7.82/8.09)
*Serratia marcescens*	23	5.12	(5.06/5.19)	6.55	(6.39/6.71)
Reporting group					
*Acinetobacter baumannii* complex	40	5.27	(5.17/5.34)	6.54	(6.43/6.70)
Enterobacterales	472	5.19	(5.17/5.21)	6.56	(6.54/6.58)
*Pseudomonas aeruginosa*	44	6.66	(6.59/6.75)	7.97	(7.82/8.09)
Total	556	5.21	(5.19/5.22)	6.58	(6.55/6.61)

^*a*^ Total TTR is defined as the duration from the point at which samples are loaded onto the PBC Separator to the time when MIC results are provided to the user. Note that this time does not include identification or monomicrobial confirmation, which is presumed to occur in parallel with AST processing. Analyzer TTR is defined as the time each sample spends in the Selux Analyzer and excludes separator and inoculator processing times.

## DISCUSSION

Technologies that consistently and substantially shorten the time between blood bottle positivity, organism identification, and complete AST results are crucial for ensuring that antimicrobial therapy can be tailored. While empiric therapy is a critical component of early sepsis treatment, narrowing patients to a tailored therapy rapidly, such as by obtaining phenotypic AST results within a short time frame, is essential for several reasons, including avoiding adverse antimicrobial effects for patients and maintaining successful antimicrobial stewardship to prevent antimicrobial resistance (27)(28–33). De-escalation of empirical therapy has also been associated with lower mortality in ICU patients (34). For example, Teshome *et al*. described in a 2019 study (35) that for each additional day of exposure to antipseudomonal beta-lactam antimicrobials, there is a significantly increased risk of antimicrobial resistance development.

The PBC Separator and the Selux AST system perform rapid AST directly from positive blood culture bottles. This substantially shortens the gap between obtaining a positive blood bottle and organism identification and the availability of a fully actionable AST result. In this multi-center clinical investigation, the PBC Separator with the Selux AST system was evaluated in comparison to the established gold standard BMD reference method. The results demonstrated satisfactory clinical performance with the same shift result generation. Moreover, the system’s consistent performance across various laboratory settings was confirmed through analytical evaluations. Specifically, the data indicated the system’s compatibility with major blood culture systems and various bottle types, thereby allowing flexibility in laboratory settings. This is important because second-generation systems demonstrate positive growth 10%–20% more rapidly on average than first-generation systems, resulting in a reduced bacterial load at the time of positivity (20, 24, 25). Furthermore, non-fastidious gram-negative samples exhibited positive growth across various bottle types and blood culture systems. The extended positive blood culture sample stability for up to 16 h prior to PBC Separator processing may facilitate increased processing time for couriers in integrated health networks or hospitals that require additional time to process these samples due to personnel limitations. The assay performance remained unaffected by endogenous and antimicrobial interferents at clinically relevant concentrations, which is of particular importance for sepsis patients, as individuals with suspected sepsis may receive oral antimicrobials prior to hospitalization, despite the recommendation that blood cultures be obtained before administering antimicrobials. Overall, this study provided the data necessary for the Selux next-generation phenotyping system to obtain FDA clearance as the first AST system capable of processing both isolates and PBC samples.

Limitations of this study include that few Enterobacterales resistant to ceftazidime-avibactam were included due to the infrequency of this phenotype during the study period. Additionally, while approximately 25% of samples were freshly collected, seeded samples were included to enrich the sample size. The interfering substances study was designed with the FDA to evaluate a wide range of clinical endogenous and exogenous interferents. However, it should be noted that some septic patients may have endogenous interferent levels beyond the extents evaluated, such as significantly elevated white blood cell counts, so further investigations assessing the potential impact of abnormal levels of interfering substances on testing accuracy in patients with sepsis may be beneficial. Lastly, it is also important to note that the system is currently only FDA-cleared for non-fastidious gram-negative organisms.

While the widespread adoption of rapid AST methodologies has the potential to dramatically improve the time to optimize antibacterial therapy in patients with sepsis, both clinical and laboratory practices may need to be adjusted to leverage these results fully. In addition to addressing personnel and space limitations, laboratory workflows should be optimized to provide bacterial identification, genotypic results, and rapid AST results concurrently to healthcare providers. This synchronization is essential for generating phenotypic AST results and guiding antimicrobial selections. Advanced technologies such as MALDI-TOF mass spectrometry and multiplex molecular platforms have revolutionized this process. These methods can identify bacterial species directly from positive blood cultures within minutes or hours, substantially reducing the time compared to traditional methods that rely on sub-culture. Multiplex molecular platforms, in particular, can accurately identify single and mixed bacterial cultures, the latter of which complicate rapid AST due to multiple species affecting accurate identification and results. Therefore, combining rapid identification and AST is particularly valuable in managing complex cases, such as polymicrobial infections, where species-specific treatment is essential. It is noteworthy that the PBC Separator evaluated in this study also produces bacterial pellets for general laboratory use; future studies to investigate the feasibility of utilizing these pellets for rapid bacterial identification could present opportunities for this system to facilitate both rapid ID and downstream rapid phenotypic AST directly from positive blood cultures.

Furthermore, it is vital to ensure that healthcare providers such as pharmacists and infectious disease physicians are adequately trained in interpreting and applying rapid AST methodologies and integrating these results into the overall treatment plan for patients with sepsis. A recent Nordic multicenter study demonstrated that while improved blood culture technologies have led to some improvements in diagnosis, limited laboratory operating hours and staffing issues have limited their effectiveness (36). The study underscores the necessity for additional resources and investment in laboratory infrastructure to fully realize the potential benefits of these novel tools. For instance, when rapid bacterial organism identification and phenotypic AST were combined with antimicrobial stewardship, this integration demonstrated the capacity to facilitate more expeditious antimicrobial modifications during treatment for gram-negative bacteremia and contribute to timely and efficacious therapy (37).

In summary, the availability of rapid AST results allows for early tailoring of antimicrobial therapy to optimize the care of patients with sepsis. This study demonstrates that the PBC Separator and the Selux AST system create an opportunity to more rapidly tailor the antimicrobial therapy of septic patients with positive blood cultures. However, it is important to note that the data in this space consistently show that a team-based healthcare approach incorporating the entire patient care team will be needed to translate these results to better treatment outcomes. In order to ensure the successful implementation of these new technologies, clinicians and laboratories must ensure that the results are effectively communicated and acted upon in a timely manner. Furthermore, careful post-implementation analysis is warranted to assess patient outcomes and ensure that the benefits of new technologies are fully realized.

## SUPPLEMENTAL MATERIAL

Supplemental tables jcm.01819-24-s0001.docx Tables S1 to S14.

ASM does not own the copyrights to Supplemental Material that may be linked to, or accessed through, an article. The authors have granted ASM a non-exclusive, world-wide license to publish the Supplemental Material files. Please contact the corresponding author directly for reuse.
