# Supplementary material for: Multi-center evaluation of the Selux next-generation phenotyping system for gram-negative direct-from-positive blood culture antimicrobial susceptibility testing
Source: J Clin Microbiol. 2025 Mar 31;63(5):e01819-24. doi: 10.1128/jcm.01819-24 (PMC12077214; doi:10.1128/jcm.01819-24)
Supplement: Supplemental tables — Tables S1 to S14. [file jcm.01819-24-s0001.docx]

**Supplementary Table 1 Antimicrobial Agent and Organism Combinations evaluated for the PBC Separator and Selux AST System**

| **Organism** | **Amikacin** | **Amoxicillin-clavulanate** | **Ampicillin** | **Ampicillin-sulbactam** | **Cefazolin** | **Cefepime** | **Ceftazidime** | **Ceftazidime-avibactam** | **Ceftriaxone** | **Ciprofloxacin** | **Ertapenem** | **Gentamicin** | **Imipenem** | **Meropenem** | **Minocycline** | **Piperacillin-tazobactam** | **Tobramycin** |
| --- | --- | --- | --- | --- | --- | --- | --- | --- | --- | --- | --- | --- | --- | --- | --- | --- | --- |
| ***Acinetobacter baumannii complex*** | X |  |  | X |  |  |  |  |  |  |  |  | X | X | X | X |  |
| ***Citrobacter freundii complex*** |  |  |  |  |  | X |  | X | X | X | X | X |  | X |  |  |  |
| ***Citrobacter koseri*** |  |  |  | X |  | X |  | X | X | X | X | X |  | X |  | X |  |
| ***Enterobacter cloacae complex*** |  |  |  |  |  | X |  | X | X | X | X | X |  | X |  |  |  |
| ***Escherichia coli*** | X | X | X | X | X | X | X | X | X | X | X | X | X | X | X | X | X |
| ***Klebsiella aerogenes*** |  |  |  |  |  | X |  | X | X | X | X | X |  |  |  |  |  |
| ***Klebsiella oxytoca*** |  | X |  |  |  | X |  | X | X | X | X | X |  | X |  |  |  |
| ***Klebsiella pneumoniae*** | X | X |  | X | X | X | X | X | X | X | X | X | X | X | X | X | X |
| ***Morganella morganii*** |  |  |  |  |  | X |  | X |  | X | X | X |  | X |  | X |  |
| ***Proteus mirabilis*** |  | X | X | X |  | X |  | X | X | X | X | X |  | X |  | X |  |
| ***Proteus vulgaris*** |  | X |  |  |  | X |  | X |  | X | X | X |  | X |  | X |  |
| ***Psuedomonas aeruginosa*** | X |  |  |  |  | X | X | X |  | X |  | X |  | X |  | X | X |
| ***Serratia marcescens*** |  |  |  |  |  | X |  | X | X | X | X | X |  | X |  | X |  |

**Supplementary Table 2 Concentrations of Endogenous Interfering Substances Utilized in the Interference Analytical Study**

| **Interfering substance** | **Evaluated Interfering substance Concentration** | **Population** | **Reference Range** |
| --- | --- | --- | --- |
| Red Blood Cells (RBCs) | 20 g/dL | Normal | 12.6-17.4 g/dL (men)  11.7-16.1 g/dL (women) |
| White Blood Cells (WBCs) | 12,000 cells/µL | Normal | 4,500 – 11,000 cells/µL |
|  |  | Severe Sepsis | >12,000 or <4,000 cells/µL |
| Platelets | 450,000 /µL | Normal | 150,000 – 450,000 /µL |
|  |  | Severe Sepsis | <150,000 /µL |
| Conjugated Bilirubin | 475 µmol/L | Normal | 0-2.4 µmol/L |
| Unconjugated Bilirubin | 684 µmol/L | Normal | 0-34 µmol/L |
| Triglycerides | 16.94 mmol/L | Normal | <1.69 mmol/L |
| Gamma Globulins | 50 g/L | Normal | 7-15 g/L |

**Supplementary Table 3 Concentrations of Exogenous Interfering Substances Utilized in the Interference Analytical Study**

| **Interfering substance** | **Class** | **Peak Serum Level for Oral Administration** | **Concentration Tested** |
| --- | --- | --- | --- |
| Cefpodoxime | **Cephalosporins** | **2.3 µg/mL** | **2.3 µg/mL** |
| Ciprofloxacin | **Fluoroquinolones** | **1.6-3.6 µg/mL** | **3.6 µg/mL** |
| Penicillin | **Penicillins** | **5-6 µg/mL** | **6 µg/mL** |
| Gentamicin | **Aminoglycosides** | **16-24 µg/mL*** | **24 µg/mL** |

***** Intramuscular injection only

**Supplementary Table 4 Results of Seeded Bacterial Concentration in Blood Bottles Study**

| **Antimicrobial Agent** | **10 CFU/bottle** | **100 CFU/bottle** | **1000 CFU/bottle** | **10,000 CFU/bottle** |
| --- | --- | --- | --- | --- |

|  | **# MIC Results** | **#EA** | **%EA** | **# MIC Results** | **#EA** | **%EA** | **# MIC Results** | **#EA** | **%EA** | **# MIC Results** | **#EA** | **%EA** |
| --- | --- | --- | --- | --- | --- | --- | --- | --- | --- | --- | --- | --- |

| **Amikacin** | **12** | **12** | **100%** | **12** | **12** | **100%** | **12** | **12** | **100%** | **12** | **12** | **100%** |
| --- | --- | --- | --- | --- | --- | --- | --- | --- | --- | --- | --- | --- |
| **Amoxicillin-clavulanate** | **4** | **4** | **100%** | **4** | **4** | **100%** | **4** | **4** | **100%** | **4** | **4** | **100%** |
| **Ampicillin** | **4** | **4** | **100%** | **4** | **4** | **100%** | **4** | **4** | **100%** | **4** | **4** | **100%** |
| **Ampicillin-sulbactam** | **8** | **8** | **100%** | **8** | **8** | **100%** | **8** | **8** | **100%** | **8** | **8** | **100%** |
| **Cefazolin** | **4** | **4** | **100%** | **4** | **4** | **100%** | **4** | **4** | **100%** | **4** | **4** | **100%** |
| **Cefepime** | **5** | **5** | **100%** | **5** | **5** | **100%** | **5** | **5** | **100%** | **6** | **6** | **100%** |
| **Ceftazidime** | **8** | **8** | **100%** | **8** | **8** | **100%** | **8** | **8** | **100%** | **8** | **8** | **100%** |
| **Ceftazidime-avibactam** | **8** | **8** | **100%** | **8** | **8** | **100%** | **8** | **8** | **100%** | **8** | **8** | **100%** |
| **Ceftriaxone** | **4** | **4** | **100%** | **4** | **4** | **100%** | **4** | **4** | **100%** | **4** | **4** | **100%** |
| **Ciprofloxacin** | **8** | **7** | **88%** | **8** | **8** | **100%** | **8** | **7** | **88%** | **8** | **8** | **100%** |
| **Ertapenem** | **4** | **4** | **100%** | **4** | **4** | **100%** | **4** | **4** | **100%** | **4** | **4** | **100%** |
| **Gentamicin** | **8** | **8** | **100%** | **8** | **8** | **100%** | **8** | **8** | **100%** | **8** | **8** | **100%** |
| **Imipenem** | **8** | **8** | **100%** | **8** | **8** | **100%** | **8** | **8** | **100%** | **8** | **8** | **100%** |
| **Meropenem** | **12** | **12** | **100%** | **12** | **12** | **100%** | **12** | **12** | **100%** | **12** | **11** | **92%** |
| **Minocycline** | **8** | **8** | **100%** | **8** | **8** | **100%** | **8** | **8** | **100%** | **8** | **8** | **100%** |
| **Piperacillin-tazobactam** | **12** | **12** | **100%** | **12** | **12** | **100%** | **12** | **12** | **100%** | **12** | **12** | **100%** |
| **Tobramycin** | **8** | **8** | **100%** | **8** | **8** | **100%** | **8** | **8** | **100%** | **8** | **8** | **100%** |
| **Total** | **125** | **124** | **99%** | **125** | **125** | **100%** | **125** | **124** | **99%** | **126** | **125** | **99%** |

**Supplementary Table 5 Results of Positive Blood Culture Sample Stability Study**

| **Antimicrobial Agent** | **4 hours after positivity** | | | **8 hours after positivity** | | | **12 hours after positivity** | | | **16 hours after positivity** | | | **18 hours after positivity** | | |
| --- | --- | --- | --- | --- | --- | --- | --- | --- | --- | --- | --- | --- | --- | --- | --- |
|  | **# MIC Results** | **#EA** | **%EA** | **# MIC Results** | **#EA** | **%EA** | **# MIC Results** | **#EA** | **%EA** | **# MIC Results** | **#EA** | **%EA** | **# MIC Results** | **#EA** | **%EA** |
| **Amikacin** | 8 | 8 | 100.0% | 8 | 8 | 100.0% | 8 | 8 | 100.0% | 18 | 18 | 100.0% | 13 | 13 | 100.0% |
| **Amoxicillin-clavulanate** | 4 | 4 | 100.0% | 4 | 4 | 100.0% | 4 | 4 | 100.0% | 14 | 14 | 100.0% | 7 | 7 | 100.0% |
| **Ampicillin** | 2 | 2 | 100.0% | 2 | 2 | 100.0% | 2 | 2 | 100.0% | 12 | 12 | 100.0% | 5 | 5 | 100.0% |
| **Ampicillin-sulbactam** | 6 | 6 | 100.0% | 6 | 6 | 100.0% | 6 | 6 | 100.0% | 16 | 16 | 100.0% | 9 | 9 | 100.0% |
| **Cefazolin** | 4 | 4 | 100.0% | 4 | 4 | 100.0% | 4 | 4 | 100.0% | 14 | 14 | 100.0% | 7 | 7 | 100.0% |
| **Cefepime** | 6 | 6 | 100.0% | 6 | 6 | 100.0% | 6 | 6 | 100.0% | 16 | 16 | 100.0% | 11 | 11 | 100.0% |
| **Ceftazidime** | 5 | 5 | 100.0% | 4 | 4 | 100.0% | 4 | 4 | 100.0% | 15 | 15 | 100.0% | 9 | 9 | 100.0% |
| **Ceftazidime-avibactam** | 6 | 6 | 100.0% | 6 | 6 | 100.0% | 6 | 6 | 100.0% | 16 | 16 | 100.0% | 11 | 11 | 100.0% |
| **Ceftriaxone** | 4 | 4 | 100.0% | 4 | 4 | 100.0% | 4 | 4 | 100.0% | 14 | 14 | 100.0% | 7 | 7 | 100.0% |
| **Ciprofloxacin** | 6 | 6 | 100.0% | 6 | 6 | 100.0% | 6 | 6 | 100.0% | 16 | 16 | 100.0% | 11 | 10 | 90.9% |
| **Ertapenem** | 4 | 4 | 100.0% | 4 | 4 | 100.0% | 4 | 4 | 100.0% | 14 | 14 | 100.0% | 7 | 7 | 100.0% |
| **Gentamicin** | 6 | 6 | 100.0% | 6 | 6 | 100.0% | 6 | 6 | 100.0% | 16 | 16 | 100.0% | 11 | 11 | 100.0% |
| **Imipenem** | 6 | 6 | 100.0% | 6 | 6 | 100.0% | 6 | 6 | 100.0% | 16 | 16 | 100.0% | 9 | 9 | 100.0% |
| **Meropenem** | 8 | 8 | 100.0% | 8 | 8 | 100.0% | 8 | 8 | 100.0% | 18 | 18 | 100.0% | 13 | 13 | 100.0% |
| **Minocycline** | 6 | 6 | 100.0% | 6 | 6 | 100.0% | 6 | 6 | 100.0% | 16 | 16 | 100.0% | 9 | 9 | 100.0% |
| **Piperacillin-tazobactam** | 8 | 7 | 87.5% | 8 | 8 | 100.0% | 8 | 8 | 100.0% | 18 | 17 | 94.4% | 13 | 12 | 92.3% |
| **Tobramycin** | 6 | 6 | 100.0% | 6 | 6 | 100.0% | 6 | 6 | 100.0% | 16 | 16 | 100.0% | 11 | 10 | 90.9% |
| **Total** | 95 | 94 | 98.9% | 94 | 94 | 100.0% | 94 | 94 | 100.0% | 265 | 264 | 99.6% | 163 | 160 | 98.2% |

**Supplementary Table 6A Results of Aerobic Blood Culture Bottle Comparability Study**

| **Antimicrobial Agent** | **BD BACTEC  Standard Aerobic** | | | **BD BACTEC  Plus Aerobic** | | | **BD BACTEC  Peds Plus** | | | **BioMérieux BacT/ALERT  SA** | | | **BioMérieux BacT/ALERT  FA Plus** | | | **BioMérieux BacT/ALERT  PF Plus** | | |
| --- | --- | --- | --- | --- | --- | --- | --- | --- | --- | --- | --- | --- | --- | --- | --- | --- | --- | --- |
|  | **# MIC Results** | **#EA** | **%EA** | **# MIC Results** | **#EA** | **%EA** | **# MIC Results** | **#EA** | **%EA** | **# MIC Results** | **#EA** | **%EA** | **# MIC Results** | **#EA** | **%EA** | **# MIC Results** | **#EA** | **%EA** |
| **Amikacin** | 25 | 25 | 100.0% | 15 | 15 | 100.0% | 15 | 14 | 93.3% | 43 | 43 | 100.0% | 15 | 15 | 100.0% | 14 | 14 | 100.0% |
| **Amoxicillin-clavulanate** | 11 | 11 | 100.0% | 10 | 10 | 100.0% | 10 | 10 | 100.0% | 28 | 28 | 100.0% | 9 | 9 | 100.0% | 8 | 8 | 100.0% |
| **Ampicillin** | 5 | 5 | 100.0% | 8 | 8 | 100.0% | 8 | 8 | 100.0% | 22 | 22 | 100.0% | 7 | 7 | 100.0% | 6 | 6 | 100.0% |
| **Ampicillin-sulbactam** | 19 | 19 | 100.0% | 13 | 13 | 100.0% | 12 | 12 | 100.0% | 37 | 37 | 100.0% | 12 | 12 | 100.0% | 11 | 11 | 100.0% |
| **Cefazolin** | 11 | 11 | 100.0% | 10 | 10 | 100.0% | 10 | 10 | 100.0% | 28 | 28 | 100.0% | 9 | 9 | 100.0% | 8 | 8 | 100.0% |
| **Cefepime** | 17 | 17 | 100.0% | 12 | 12 | 100.0% | 13 | 13 | 100.0% | 34 | 34 | 100.0% | 12 | 12 | 100.0% | 11 | 11 | 100.0% |
| **Ceftazidime** | 16 | 16 | 100.0% | 11 | 11 | 100.0% | 12 | 12 | 100.0% | 31 | 31 | 100.0% | 11 | 11 | 100.0% | 11 | 11 | 100.0% |
| **Ceftazidime-avibactam** | 17 | 17 | 100.0% | 12 | 12 | 100.0% | 13 | 13 | 100.0% | 34 | 34 | 100.0% | 12 | 12 | 100.0% | 11 | 11 | 100.0% |
| **Ceftriaxone** | 11 | 11 | 100.0% | 10 | 10 | 100.0% | 10 | 10 | 100.0% | 28 | 28 | 100.0% | 9 | 9 | 100.0% | 8 | 8 | 100.0% |
| **Ciprofloxacin** | 17 | 17 | 100.0% | 12 | 12 | 100.0% | 13 | 13 | 100.0% | 34 | 31 | 91.2% | 12 | 12 | 100.0% | 11 | 11 | 100.0% |
| **Ertapenem** | 11 | 11 | 100.0% | 10 | 10 | 100.0% | 10 | 10 | 100.0% | 28 | 28 | 100.0% | 9 | 9 | 100.0% | 8 | 8 | 100.0% |
| **Gentamicin** | 17 | 17 | 100.0% | 12 | 12 | 100.0% | 13 | 13 | 100.0% | 34 | 34 | 100.0% | 12 | 12 | 100.0% | 11 | 11 | 100.0% |
| **Imipenem** | 19 | 19 | 100.0% | 13 | 13 | 100.0% | 12 | 12 | 100.0% | 37 | 37 | 100.0% | 12 | 12 | 100.0% | 11 | 11 | 100.0% |
| **Meropenem** | 25 | 25 | 100.0% | 15 | 15 | 100.0% | 15 | 15 | 100.0% | 43 | 43 | 100.0% | 15 | 15 | 100.0% | 14 | 14 | 100.0% |
| **Minocycline** | 19 | 19 | 100.0% | 13 | 12 | 92.3% | 12 | 10 | 83.3% | 37 | 37 | 100.0% | 12 | 11 | 91.7% | 11 | 10 | 90.9% |
| **Piperacillin-tazobactam** | 25 | 25 | 100.0% | 15 | 15 | 100.0% | 15 | 15 | 100.0% | 43 | 41 | 95.3% | 15 | 15 | 100.0% | 14 | 14 | 100.0% |
| **Tobramycin** | 17 | 17 | 100.0% | 12 | 12 | 100.0% | 13 | 13 | 100.0% | 34 | 34 | 100.0% | 12 | 12 | 100.0% | 11 | 11 | 100.0% |
| **Total** | 282 | 282 | 100.0% | 203 | 202 | 99.5% | 206 | 203 | 98.5% | 575 | 570 | 99.1% | 195 | 194 | 99.5% | 179 | 178 | 99.4% |

**Supplementary Table 6B Results of Anaerobic Blood Culture Bottle Comparability Study**

| **Antimicrobial Agent** | **BD BACTEC  Standard Anaerobic** | | | **BD BACTEC  Plus Anaerobic** | | | **BD BACTEC  Lytic Anaerobic** | | | **BioMérieux BacT/ALERT  SN** | | | **BioMérieux BacT/ALERT  FN Plus** | | |
| --- | --- | --- | --- | --- | --- | --- | --- | --- | --- | --- | --- | --- | --- | --- | --- |
|  | **# MIC Results** | **#EA** | **%EA** | **# MIC Results** | **#EA** | **%EA** | **# MIC Results** | **#EA** | **%EA** | **# MIC Results** | **#EA** | **%EA** | **# MIC Results** | **#EA** | **%EA** |
| **Amikacin** | 13 | 13 | 100.0% | 9 | 9 | 100.0% | 10 | 10 | 100.0% | 6 | 6 | 100.0% | 9 | 9 | 100.0% |
| **Amoxicillin-clavulanate** | 15 | 15 | 100.0% | 11 | 11 | 100.0% | 12 | 12 | 100.0% | 8 | 8 | 100.0% | 11 | 11 | 100.0% |
| **Ampicillin** | 13 | 13 | 100.0% | 9 | 9 | 100.0% | 10 | 10 | 100.0% | 6 | 6 | 100.0% | 9 | 9 | 100.0% |
| **Ampicillin-sulbactam** | 17 | 17 | 100.0% | 13 | 13 | 100.0% | 14 | 13 | 92.9% | 10 | 10 | 100.0% | 13 | 13 | 100.0% |
| **Cefazolin** | 13 | 13 | 100.0% | 9 | 9 | 100.0% | 10 | 10 | 100.0% | 6 | 6 | 100.0% | 9 | 9 | 100.0% |
| **Cefepime** | 17 | 17 | 100.0% | 13 | 13 | 100.0% | 14 | 14 | 100.0% | 10 | 10 | 100.0% | 13 | 13 | 100.0% |
| **Ceftazidime** | 13 | 13 | 100.0% | 9 | 9 | 100.0% | 10 | 10 | 100.0% | 6 | 6 | 100.0% | 9 | 9 | 100.0% |
| **Ceftazidime-avibactam** | 17 | 17 | 100.0% | 13 | 13 | 100.0% | 14 | 14 | 100.0% | 10 | 10 | 100.0% | 13 | 13 | 100.0% |
| **Ceftriaxone** | 15 | 15 | 100.0% | 11 | 11 | 100.0% | 12 | 12 | 100.0% | 8 | 8 | 100.0% | 11 | 11 | 100.0% |
| **Ciprofloxacin** | 17 | 16 | 94.1% | 13 | 13 | 100.0% | 14 | 12 | 85.7% | 10 | 10 | 100.0% | 13 | 12 | 92.3% |
| **Ertapenem** | 17 | 17 | 100.0% | 13 | 13 | 100.0% | 14 | 14 | 100.0% | 10 | 10 | 100.0% | 13 | 13 | 100.0% |
| **Gentamicin** | 17 | 17 | 100.0% | 13 | 13 | 100.0% | 14 | 14 | 100.0% | 10 | 10 | 100.0% | 13 | 13 | 100.0% |
| **Imipenem** | 13 | 13 | 100.0% | 9 | 9 | 100.0% | 10 | 10 | 100.0% | 6 | 6 | 100.0% | 9 | 9 | 100.0% |
| **Meropenem** | 17 | 17 | 100.0% | 13 | 13 | 100.0% | 14 | 14 | 100.0% | 10 | 10 | 100.0% | 13 | 13 | 100.0% |
| **Minocycline** | 13 | 13 | 100.0% | 9 | 9 | 100.0% | 10 | 10 | 100.0% | 6 | 6 | 100.0% | 9 | 9 | 100.0% |
| **Piperacillin-tazobactam** | 17 | 17 | 100.0% | 13 | 13 | 100.0% | 14 | 14 | 100.0% | 10 | 10 | 100.0% | 13 | 13 | 100.0% |
| **Tobramycin** | 13 | 13 | 100.0% | 9 | 9 | 100.0% | 10 | 10 | 100.0% | 6 | 6 | 100.0% | 9 | 9 | 100.0% |
| **Total** | 257 | 256 | 99.6% | 189 | 189 | 100.0% | 206 | 203 | 98.5% | 138 | 138 | 100.0% | 189 | 188 | 99.5% |

**Supplementary Table 7 Results of Blood Culture System Comparability Study**

| **Antimicrobial Agent** | **BioMérieux BACT/ALERT VIRTUO** | | | **BD BACTEC 9050** | | |
| --- | --- | --- | --- | --- | --- | --- |
|  | **# MIC Results** | **#EA** | **%EA** | **# MIC Results** | **#EA** | **%EA** |
| Amikacin | 94 | 94 | 100.0% | 31 | 31 | 100.0% |
| Amoxicillin-clavulanate | 42 | 42 | 100.0% | 15 | 15 | 100.0% |
| Ampicillin | 35 | 35 | 100.0% | 9 | 9 | 100.0% |
| Ampicillin-sulbactam | 70 | 70 | 100.0% | 23 | 23 | 100.0% |
| Cefazolin | 42 | 42 | 100.0% | 15 | 15 | 100.0% |
| Cefepime | 66 | 65 | 98.5% | 23 | 23 | 100.0% |
| Ceftazidime | 50 | 50 | 100.0% | 20 | 20 | 100.0% |
| Ceftazidime-avibactam | 66 | 66 | 100.0% | 23 | 23 | 100.0% |
| Ceftriaxone | 42 | 42 | 100.0% | 15 | 15 | 100.0% |
| Ciprofloxacin | 66 | 63 | 95.5% | 23 | 23 | 100.0% |
| Ertapenem | 42 | 42 | 100.0% | 15 | 15 | 100.0% |
| Gentamicin | 66 | 66 | 100.0% | 23 | 23 | 100.0% |
| Imipenem | 70 | 70 | 100.0% | 23 | 23 | 100.0% |
| Meropenem | 94 | 93 | 98.9% | 31 | 31 | 100.0% |
| Minocycline | 70 | 70 | 100.0% | 23 | 23 | 100.0% |
| Piperacillin-tazobactam | 94 | 90 | 95.7% | 31 | 31 | 100.0% |
| Tobramycin | 66 | 66 | 100.0% | 23 | 23 | 100.0% |
| **Total** | **1075** | **1066** | **99.2%** | **366** | **366** | **100.0%** |

**Supplementary Table 8 Results of the Endogenous Interfering Substance Study**

| **Antimicrobial Agent** | **Red Blood Cells** | | | **White Blood Cells** | | | **Platelets** | | | **Unconjugated Bilirubin** | | | **Conjugated Bilirubin** | | | **Triglycerides** | | | **Gamma Globulins** | | |
| --- | --- | --- | --- | --- | --- | --- | --- | --- | --- | --- | --- | --- | --- | --- | --- | --- | --- | --- | --- | --- | --- |
|  | **# MIC Results** | **#EA** | **%EA** | **# MIC Results** | **#EA** | **%EA** | **# MIC Results** | **#EA** | **%EA** | **# MIC Results** | **#EA** | **%EA** | **# MIC Results** | **#EA** | **%EA** | **# MIC Results** | **#EA** | **%EA** | **# MIC Results** | **#EA** | **%EA** |
| Amikacin | 15 | 15 | 100.0% | 14 | 14 | 100.0% | 12 | 12 | 100.0% | 15 | 15 | 100.0% | 15 | 15 | 100.0% | 11 | 10 | 90.9% | 17 | 17 | 100.0% |
| Amoxicillin-clavulanate | 11 | 11 | 100.0% | 5 | 5 | 100.0% | 5 | 5 | 100.0% | 11 | 11 | 100.0% | 11 | 11 | 100.0% | 7 | 7 | 100.0% | 10 | 10 | 100.0% |
| Ampicillin | 9 | 9 | 100.0% | 4 | 4 | 100.0% | 4 | 4 | 100.0% | 9 | 9 | 100.0% | 9 | 9 | 100.0% | 5 | 5 | 100.0% | 8 | 8 | 100.0% |
| Ampicillin-sulbactam | 13 | 13 | 100.0% | 9 | 9 | 100.0% | 10 | 10 | 100.0% | 13 | 13 | 100.0% | 13 | 13 | 100.0% | 10 | 10 | 100.0% | 14 | 14 | 100.0% |
| Cefazolin | 11 | 11 | 100.0% | 5 | 5 | 100.0% | 5 | 3 | 60.0% | 11 | 11 | 100.0% | 11 | 11 | 100.0% | 7 | 7 | 100.0% | 10 | 8 | 80.0% |
| Cefepime | 13 | 13 | 100.0% | 10 | 10 | 100.0% | 9 | 9 | 100.0% | 13 | 13 | 100.0% | 13 | 13 | 100.0% | 10 | 10 | 100.0% | 14 | 14 | 100.0% |
| Ceftazidime | 12 | 12 | 100.0% | 9 | 9 | 100.0% | 9 | 9 | 100.0% | 12 | 12 | 100.0% | 13 | 13 | 100.0% | 10 | 10 | 100.0% | 12 | 12 | 100.0% |
| Ceftazidime-avibactam | 13 | 13 | 100.0% | 10 | 10 | 100.0% | 9 | 9 | 100.0% | 13 | 13 | 100.0% | 13 | 13 | 100.0% | 10 | 10 | 100.0% | 14 | 14 | 100.0% |
| Ceftriaxone | 11 | 11 | 100.0% | 5 | 5 | 100.0% | 5 | 5 | 100.0% | 11 | 11 | 100.0% | 11 | 11 | 100.0% | 7 | 7 | 100.0% | 10 | 10 | 100.0% |
| Ciprofloxacin | 13 | 13 | 100.0% | 10 | 10 | 100.0% | 9 | 9 | 100.0% | 13 | 13 | 100.0% | 13 | 13 | 100.0% | 10 | 10 | 100.0% | 14 | 14 | 100.0% |
| Ertapenem | 11 | 11 | 100.0% | 5 | 5 | 100.0% | 5 | 5 | 100.0% | 11 | 11 | 100.0% | 11 | 11 | 100.0% | 7 | 7 | 100.0% | 10 | 10 | 100.0% |
| Gentamicin | 13 | 13 | 100.0% | 10 | 10 | 100.0% | 9 | 9 | 100.0% | 13 | 13 | 100.0% | 13 | 13 | 100.0% | 10 | 10 | 100.0% | 14 | 14 | 100.0% |
| Imipenem | 13 | 13 | 100.0% | 9 | 9 | 100.0% | 10 | 10 | 100.0% | 13 | 13 | 100.0% | 13 | 13 | 100.0% | 10 | 10 | 100.0% | 14 | 14 | 100.0% |
| Meropenem | 15 | 15 | 100.0% | 14 | 14 | 100.0% | 14 | 14 | 100.0% | 15 | 15 | 100.0% | 15 | 15 | 100.0% | 13 | 13 | 100.0% | 18 | 18 | 100.0% |
| Minocycline | 13 | 13 | 100.0% | 9 | 9 | 100.0% | 10 | 10 | 100.0% | 13 | 13 | 100.0% | 13 | 13 | 100.0% | 10 | 10 | 100.0% | 14 | 14 | 100.0% |
| Piperacillin-tazobactam | 15 | 13 | 86.7% | 14 | 13 | 92.9% | 14 | 11 | 78.6% | 15 | 13 | 86.7% | 15 | 12 | 80.0% | 13 | 10 | 76.9% | 18 | 16 | 88.9% |
| Tobramycin | 13 | 12 | 92.3% | 10 | 10 | 100.0% | 9 | 8 | 88.9% | 13 | 12 | 92.3% | 13 | 12 | 92.3% | 10 | 9 | 90.0% | 14 | 13 | 92.9% |
| **Total** | 214 | 211 | 98.6% | 152 | 151 | 99.3% | 148 | 142 | 95.9% | 214 | 211 | 98.6% | 215 | 211 | 98.1% | 160 | 155 | 96.9% | 225 | 220 | 97.8% |

**Supplementary Table 9 Results of the Exogenous Interfering Substance Study**

| **Antimicrobial Interferent** | **# MIC Results** | **#EA** | **%EA** |
| --- | --- | --- | --- |
| Cefpodoxime | 179 | 172 | 96.1% |
| Ciprofloxacin | 196 | 190 | 96.9% |
| Gentamicin | 197 | 190 | 96.4% |
| Penicillin | 214 | 207 | 96.7% |

**Supplementary Table 10: Resistance Mechanisms of the Challenge Samples Included in the Clinical Evaluation**

| **Drug** | **Drug Class Specific** | **Efflux Pump Genes** | **Truncated Porin Genes** |
| --- | --- | --- | --- |
| AMC | CMY-2, CMY-4, CMY-23, CMY-42, CTX-M-2, CTX-M-14, CTX-M-15, CTX-M-27, CTX-M-55, CTX-M-65, CTX-M-14b, DHA1, EC-5, EC-13, KPC-2, KPC-3, KPC-11, LAP-1, LAP-2, LEN-27, NDM-1, OKP-B-2, OXA-1, OXA-2, OXA-9, OXA-10, OXA-48, OXA-181, OXA-232, SHV-11, SHV-12, SHV-26, SHV-27, SHV-28, SHV-38, SHV-99, SHV-100, TEM-1, TEM-1B, TEM-1A, TEM-52B | ACRF, EMRD, KDEA | Omp35, OmpF, OmpK35, OmpK36 |
| AMK | aac(6')-Ib, aac(6')-Ib-AKT, aac(6')-Ib-cr, aadA1, aadA2, aadA5, aadB, ant(2")-la, aph(3')-Ia, aph(3')-Ib, aph(3')-Ic, aph(3')-VIa, aph(4)-Ia, aph(6)-Id, armA, rmtB, rmtD2, sat-2A, strA, strB | ACRF, EMRD, KDEA | Omp35, OmpF, OmpK35, OmpK36 |
| AMP | CMY-2, CMY-42, CTX-M-14, CTX-M-15, CTX-M-27, CTX-M-55, CTX-M-65, CTX-M-14b, EC-5, EC-13, KPC-3, LAP-1, OXA-1, OXA-10, SHV-11, TEM-1, TEM-1B, TEM-52B | ACRF, EMRD | OmpF |
| CAZ | CMY-2, CMY-4, CMY-23, CMY-42, CTX-M-2, CTX-M-14, CTX-M-15, CTX-M-27, CTX-M-55, CTX-M-65, CTX-M-14b, DHA1, EC-5, EC-13, GES-1, GES-19, GES-20, KPC-2, KPC-3, KPC-11, LAP-1, LAP-2, LEN-27, NDM-1, OKP-B-2, OXA-1, OXA-2, OXA-4, OXA-9, OXA-10, OXA-48, OXA-50, OXA-181, OXA-232, PAO, SHV-11, SHV-12, SHV-26, SHV-27, SHV-28, SHV-38, SHV-99, SHV-100, TEM-1, TEM-1B, TEM-1A, TEM-52B, VEB-1, VIM-2, VIM-11 | ACRF, EMRD, KDEA | Omp35, OmpF, OmpK35, OmpK36 |
| CFZ | CMY-2, CMY-4, CMY-23, CMY-42, CTX-M-2, CTX-M-14, CTX-M-15, CTX-M-27, CTX-M-55, CTX-M-65, CTX-M-14b, DHA1, EC-5, EC-13, KPC-2, KPC-3, KPC-11, LAP-1, LAP-2, LEN-27, NDM-1, OKP-B-2, OXA-1, OXA-2, OXA-9, OXA-10, OXA-48, OXA-181, OXA-232, SHV-11, SHV-12, SHV-26, SHV-27, SHV-28, SHV-38, SHV-99, SHV-100, TEM-1, TEM-1B, TEM-1A, TEM-52B | ACRF, EMRD, KDEA | Omp35, OmpF, OmpK35, OmpK36 |
| CIP | oqxA, oqxB, oqxB20, QnrB1, QnrB4, QnrS1 | ACRF, EMRD, KDEA | Omp35, OmpF, OmpK35, OmpK36 |
| CRO | ACT-7, CMY-2, CMY-4, CMY-23, CMY-42, CTX-M-2, CTX-M-14, CTX-M-15, CTX-M-27, CTX-M-55, CTX-M-65, CTX-M-14b, DHA1, EC-5, EC-13, KPC-2, KPC-3, KPC-11, LAP-1, LAP-2, LEN-27, NDM-1, NMC-A, OKP-B-2, OXA-1, OXA-2, OXA-9, OXA-10, OXA-48, OXA-181, OXA-232, SHV-11, SHV-12, SHV-26, SHV-27, SHV-28, SHV-38, SHV-99, SHV-100, TEM-1, TEM-1B, TEM-1A, TEM-52B | ACRF, EMRD, KDEA | Omp35, OmpF, OmpK35, OmpK36 |
| CZA | ACT-7, CMY-2, CMY-4, CMY-23, CMY-42, CTX-M-2, CTX-M-14, CTX-M-15, CTX-M-27, CTX-M-55, CTX-M-65, CTX-M-14b, DHA1, EC-5, EC-13, GES-1, GES-19, GES-20, KPC-2, KPC-3, KPC-11, LAP-1, LAP-2, LEN-27, NDM-1, NMC-A, OKP-B-2, OXA-1, OXA-2, OXA-4, OXA-9, OXA-10, OXA-48, OXA-50, OXA-181, OXA-232, PAO, SHV-11, SHV-12, SHV-26, SHV-27, SHV-28, SHV-38, SHV-99, SHV-100, TEM-1, TEM-1B, TEM-1A, TEM-52B, VEB-1, VIM-2, VIM-11 | ACRF, EMRD, KDEA | Omp35, OmpF, OmpK35, OmpK36 |
| ETP | ACT-7, CMY-2, CMY-4, CMY-42, CTX-M-2, CTX-M-14, CTX-M-15, CTX-M-27, CTX-M-55, CTX-M-65, CTX-M-14b, DHA1, EC-5, EC-13, KPC-2, KPC-3, KPC-11, LAP-1, LAP-2, LEN-27, NDM-1, NMC-A, OKP-B-2, OXA-1, OXA-2, OXA-9, OXA-10, OXA-48, OXA-181, OXA-232, SHV-11, SHV-12, SHV-26, SHV-27, SHV-28, SHV-38, SHV-99, SHV-100, TEM-1, TEM-1A, TEM-1B, TEM-52B | ACRF, EMRD, KDEA | Omp35, OmpK35, OmpK36 |
| FEP | ACT-7, CMY-2, CMY-4, CMY-23, CMY-42, CTX-M-2, CTX-M-14, CTX-M-15, CTX-M-27, CTX-M-55, CTX-M-65, DHA1, EC-5, EC-13, GES-1, GES-19, GES-20, KPC-2, KPC-3, KPC-11, LAP-1, LAP-2, LEN-27, NDM-1, NMC-A, OKP-B-2, OXA-1, OXA-2, OXA-4, OXA-9, OXA-10, OXA-48, OXA-50, OXA-181, OXA-232, PAO, SHV-11, SHV-12, SHV-26, SHV-27, SHV-28, SHV-99, SHV-100, TEM-1, TEM-1B, TEM-1A, VEB-1, VIM-2, VIM-11 | ACRF, EMRD, KDEA | Omp35, OmpF, OmpK35, OmpK36 |
| GEN | aac(6')-Ib, aac(6')-Ib-AKT, aac(6')-Ib-cr, aadA1, aadA2, aadA5, aadB, ant(2")-la, aph(3')-Ia, aph(3')-Ib, aph(3')-Ic, aph(4)-Ia, aph(6)-Id, armA, rmtB, rmtD2, sat-2A, strA, strB | ACRF, EMRD, KDEA | Omp35, OmpF, OmpK35, OmpK36 |
| IMP | ADC-25, CMY-2, CMY-4, CMY-23, CMY-42, CTX-M-2, CTX-M-14, CTX-M-15, CTX-M-27, CTX-M-55, CTX-M-65, CTX-M-14b, DHA1, EC-5, EC-13, KPC-2, KPC-3, KPC-11, LAP-1, LAP-2, LEN-27, NDM-1, OKP-B-2, OXA-1, OXA-2, OXA-9, OXA-10, OXA-23, OXA-24, OXA-48, OXA-66, OXA-69, OXA-72, OXA-82, OXA-181, OXA-203, OXA-232, PER-7, SHV-11, SHV-12, SHV-26, SHV-27, SHV-28, SHV-38, SHV-99, SHV-100, TEM-1, TEM-1D, TEM-1B, TEM-1A, TEM-52B | ACRF, EMRD, KDEA | Omp35, OmpF, OmpK35, OmpK36 |
| MEM | ACT-7, ADC-25, CMY-2, CMY-4, CMY-23, CMY-42, CTX-M-2, CTX-M-14, CTX-M-15, CTX-M-27, CTX-M-55, CTX-M-65, CTX-M-14b, EC-5, EC-13, GES-1, GES-19, GES-20, KPC-2, KPC-3, KPC-11, LAP-1, LAP-2, LEN-27, NDM-1, NMC-A, OKP-B-2, OXA-1, OXA-2, OXA-4, OXA-9, OXA-10, OXA-23, OXA-24, OXA-48, OXA-50, OXA-66, OXA-69, OXA-72, OXA-82, OXA-181, OXA-203, OXA-232, PAO, PER-7, SHV-11, SHV-12, SHV-26, SHV-27, SHV-28, SHV-38, SHV-99, SHV-100, TEM-1, TEM-1D, TEM-1B, TEM-1A, TEM-52B, VEB-1, VIM-2, VIM-11 | ACRF, EMRD, KDEA | Omp35, OmpF, OmpK35, OmpK36 |
| MIN | tet(A), tet(B), tet(C), tet(D), tet(G), tet(R) | ACRF, EMRD, KDEA | Omp35, OmpF, OmpK35, OmpK36 |
| SAM | ADC-25, CMY-2, CMY-4, CMY-23, CMY-42, CTX-M-2, CTX-M-14, CTX-M-15, CTX-M-27, CTX-M-55, CTX-M-65, CTX-M-14b, DHA1, EC-5, EC-13, KPC-2, KPC-3, KPC-11, LAP-1, LAP-2, LEN-27, NDM-1, OKP-B-2, OXA-1, OXA-2, OXA-9, OXA-10, OXA-23, OXA-24, OXA-48, OXA-66, OXA-69, OXA-72, OXA-82, OXA-181, OXA-203, OXA-232, PER-7, SHV-11, SHV-12, SHV-26, SHV-27, SHV-28, SHV-38, SHV-99, SHV-100, TEM-1, TEM-1D, TEM-1B, TEM-1A, TEM-52B | ACRF, EMRD, KDEA | Omp35, OmpF, OmpK35, OmpK36 |
| TOB | aac(6')-Ib, aac(6')-Ib-AKT, aac(6')-Ib-cr, aadA1, aadA2, aadA5, aadB, aph(3')-Ia, aph(3')-Ib, aph(3')-Ic, aph(3')-VI, aph(4)-Ia, aph(6)-Id, armA, rmtB, rmtD2, sat-2A, strA, strB | ACRF, EMRD, KDEA | Omp35, OmpF, OmpK35, OmpK36 |
| TZP | ADC-25, CMY-2, CMY-4, CMY-23, CMY-42, CTX-M-2, CTX-M-14, CTX-M-15, CTX-M-27, CTX-M-55, CTX-M-65, CTX-M-14b, DHA1, EC-5, EC-13, GES-1, GES-19, GES-20, KPC-2, KPC-3, KPC-11, LAP-1, LAP-2, LEN-27, NDM-1, OKP-B-2, OXA-1, OXA-2, OXA-4, OXA-9, OXA-10, OXA-23, OXA-24, OXA-48, OXA-50, OXA-66, OXA-69, OXA-72, OXA-82, OXA-181, OXA-203, OXA-232, PAO, PER-7, SHV-11, SHV-12, SHV-26, SHV-27, SHV-28, SHV-99, SHV-100, TEM-1, TEM-1D, TEM-1B, TEM-1A, TEM-52B, VEB-1, VIM-2, VIM-11 | ACRF, EMRD, KDEA | Omp35, OmpF, OmpK35, OmpK36 |

**Supplementary Table 11 organism phenotypic AST profile**

| **Accession** | **Organism ID** | **Organism Reporting Group** | **Sample Type** | **Reference Result** |
| --- | --- | --- | --- | --- |
| SPBC-2001 | Escherichia coli | Enterobacterales | Seeded Clinical | AMC S, AMK S, AMP S, CAZ R, CFZ S, CIP R, CRO R, CZA S, ETP R, FEP R, GEN S, IMP R, MEM R, MIN S, SAM S, TOB S, TZP S |
| SPBC-2002 | Escherichia coli | Enterobacterales | Seeded Clinical | AMC S, AMK S, AMP R, CAZ S, CFZ R, CIP R, CRO R, CZA R, ETP R, FEP R, GEN R, IMP R, MEM R, MIN S, SAM R, TOB I, TZP S |
| SPBC-2003 | Escherichia coli | Enterobacterales | Seeded Clinical | AMC S, AMK S, AMP S, CAZ S, CFZ S, CIP R, CRO R, CZA R, ETP R, FEP R, GEN R, IMP R, MEM R, MIN S, SAM S, TOB S, TZP R |
| SPBC-2004 | Escherichia coli | Enterobacterales | Seeded Clinical | AMC S, CIP R, GEN S, SAM I |
| SPBC-1008 | Escherichia coli | Enterobacterales | Seeded Clinical | AMC S, AMK S, AMP S, CAZ S, CFZ S, CIP R, CRO R, CZA R, ETP R, FEP R, GEN R, IMP R, MEM R, MIN S, SAM S, TOB S, TZP S |
| SPBC-3001 | Escherichia coli | Enterobacterales | Seeded Clinical | AMC S, AMK S, AMP S, CAZ S, CFZ S, CIP R, CRO R, CZA S, ETP R, FEP R, GEN R, IMP R, MEM R, MIN S, SAM S, TOB S, TZP S |
| SPBC-1002 | Escherichia coli | Enterobacterales | Seeded Clinical | AMC S, AMK S, AMP R, CAZ S, CFZ S, CIP S, CRO R, CZA R, ETP R, FEP R, GEN R, IMP R, MEM R, MIN S, SAM I, TOB S, TZP S |
| SPBC-1009 | Escherichia coli | Enterobacterales | Seeded Clinical | AMC S, AMK R, AMP R, CAZ S, CFZ S, CIP R, CRO R, CZA S, ETP R, FEP S, GEN R, IMP R, MEM R, MIN S, SAM I, TOB S, TZP S |
| SPBC-2005 | Escherichia coli | Enterobacterales | Seeded Clinical | AMC S, CIP R, GEN R, SAM S |
| SPBC-3002 | Escherichia coli | Enterobacterales | Seeded Clinical | AMC S, AMK S, AMP R, CAZ S, CFZ S, CIP R, CRO R, CZA S, ETP R, FEP R, GEN R, IMP R, MEM R, MIN S, SAM I, TOB S, TZP S |
| SPBC-3003 | Escherichia coli | Enterobacterales | Seeded Clinical | AMC S, AMK S, AMP S, CAZ R, CFZ S, CIP R, CRO R, CZA R, ETP R, FEP R, GEN R, IMP R, MEM R, MIN S, SAM S, TOB S, TZP S |
| SPBC-1006 | Escherichia coli | Enterobacterales | Seeded Clinical | AMC S, AMK S, AMP S, CAZ S, CFZ S, CIP R, CRO R, CZA S, ETP R, FEP R, GEN R, IMP R, MEM R, MIN S, SAM S, TOB S, TZP S |
| SPBC-2062 | Escherichia coli | Enterobacterales | Seeded Clinical | AMC S, AMK S, AMP R, CAZ S, CFZ S, CIP R, CRO R, CZA R, ETP R, FEP R, GEN R, IMP R, MEM R, MIN S, SAM R, TOB S, TZP S |
| SPBC-2063 | Escherichia coli | Enterobacterales | Seeded Clinical | AMC R, CIP R, GEN S, SAM R |
| SPBC-2064 | Escherichia coli | Enterobacterales | Seeded Clinical | AMC S, CIP R, GEN R, SAM S |
| SPBC-2065 | Escherichia coli | Enterobacterales | Seeded Clinical | AMC I, CIP R, GEN S, SAM R |
| SPBC-2066 | Escherichia coli | Enterobacterales | Seeded Clinical | AMC I, AMK S, AMP R, CAZ I, CFZ R, CIP R, CRO S, CZA S, ETP S, FEP S, GEN R, IMP R, MEM R, MIN S, SAM R, TOB S, TZP S |
| SPBC-3032 | Escherichia coli | Enterobacterales | Seeded Clinical | AMC S, AMK S, AMP R, CAZ S, CFZ S, CIP S, CRO R, CZA R, ETP R, FEP R, GEN R, IMP R, MEM R, MIN R, TOB S, TZP S |
| SPBC-3033 | Escherichia coli | Enterobacterales | Seeded Clinical | AMC S, AMK S, AMP S, CAZ S, CFZ S, CIP R, CRO R, CZA R, ETP R, FEP R, GEN R, IMP R, MEM R, MIN S, SAM S, TOB S, TZP S |
| SPBC-3034 | Escherichia coli | Enterobacterales | Seeded Clinical | AMC S, AMK S, AMP R, CAZ I, CFZ R, CIP R, CRO R, CZA R, ETP R, FEP R, GEN R, IMP R, MEM R, MIN S, SAM R, TOB R, TZP S |
| SPBC-2084 | Escherichia coli | Enterobacterales | Seeded Clinical | AMC S, AMK S, AMP R, CAZ S, CFZ S, CIP R, CRO R, CZA S, ETP R, FEP S, GEN R, IMP R, MEM R, MIN S, SAM I, TOB S, TZP S |
| SPBC-2085 | Escherichia coli | Enterobacterales | Seeded Clinical | AMC S, AMK S, AMP S, CAZ S, CFZ S, CIP S, CRO R, CZA S, ETP R, FEP R, GEN R, IMP R, MEM R, MIN S, SAM S, TOB S, TZP S |
| SPBC-1003 | Escherichia coli | Enterobacterales | Seeded Clinical | AMC I, AMK S, AMP R, CAZ S, CFZ I, CIP R, CRO R, CZA R, ETP R, FEP R, GEN R, IMP S, MEM R, MIN S, SAM R, TOB S, TZP S |
| SPBC-2086 | Escherichia coli | Enterobacterales | Seeded Clinical | AMC S, AMK S, AMP S, CAZ R, CFZ S, CIP S, CRO R, CZA S, ETP R, FEP R, GEN R, IMP R, MEM R, MIN S, SAM S, TOB S, TZP S |
| SPBC-3035 | Escherichia coli | Enterobacterales | Seeded Clinical | AMC S, AMK S, AMP R, CAZ S, CFZ S, CIP R, CRO R, CZA S, ETP R, FEP R, GEN R, IMP R, MEM R, MIN S, SAM I, TOB S, TZP S |
| SPBC-2087 | Escherichia coli | Enterobacterales | Seeded Clinical | AMC I, AMK S, AMP R, CAZ R, CFZ R, CIP R, CRO R, CZA S, ETP S, FEP R, GEN R, IMP R, MEM R, MIN S, SAM R, TOB R, TZP R |
| SPBC-2089 | Escherichia coli | Enterobacterales | Seeded Clinical | AMC S, GEN R, SAM R |
| SPBC-3048 | Escherichia coli | Enterobacterales | Seeded Clinical | AMC S, AMK S, AMP R, CAZ S, CFZ S, CIP R, CRO R, CZA S, ETP R, FEP R, GEN S, IMP R, MEM R, MIN S, SAM I, TOB S, TZP S |
| SPBC-2112 | Escherichia coli | Enterobacterales | Seeded Clinical | AMC I, CIP R, GEN R, SAM R |
| SPBC-3055 | Escherichia coli | Enterobacterales | Seeded Clinical | AMC S, AMK S, AMP S, CAZ S, CFZ S, CIP R, CRO R, CZA S, ETP R, FEP R, GEN R, IMP R, MEM R, MIN S, SAM S, TOB S, TZP S |
| SPBC-1004 | Escherichia coli | Enterobacterales | Seeded Clinical | AMC R, AMK R, AMP R, CAZ R, CFZ R, CIP R, CRO R, CZA S, FEP SDD, GEN R, IMP R, MEM S, MIN S, SAM R, TOB S, TZP R |
| SPBC-2124 | Escherichia coli | Enterobacterales | Seeded Clinical | AMC I, AMK S, AMP R, CAZ S, CFZ S, CIP R, CRO R, CZA S, ETP R, FEP R, GEN I, IMP R, MEM R, MIN R, SAM R, TZP S |
| SPBC-2125 | Escherichia coli | Enterobacterales | Seeded Clinical | AMC S, AMK S, AMP S, CAZ S, CFZ S, CIP R, CRO R, CZA S, ETP S, FEP R, GEN R, IMP R, MEM R, MIN S, SAM S, TOB S, TZP S |
| SPBC-3056 | Escherichia coli | Enterobacterales | Seeded Clinical | AMC S, AMK S, AMP R, CAZ S, CFZ R, CIP R, CRO R, CZA S, ETP R, FEP R, GEN R, IMP R, MEM R, MIN S, SAM R, TOB S, TZP S |
| SPBC-2126 | Escherichia coli | Enterobacterales | Seeded Clinical | AMC S, CIP R, GEN R, SAM S |
| SPBC-2127 | Escherichia coli | Enterobacterales | Seeded Clinical | AMC S, CIP R, GEN R, SAM S |
| SPBC-3057 | Escherichia coli | Enterobacterales | Seeded Clinical | AMC S, AMK S, AMP S, CAZ S, CFZ S, CIP R, CRO R, CZA S, ETP R, FEP R, GEN R, IMP R, MEM R, MIN S, SAM S, TOB S, TZP S |
| SPBC-1007 | Escherichia coli | Enterobacterales | Seeded Clinical | AMC S, AMK S, AMP S, CAZ S, CFZ S, CIP R, CRO R, CZA R, ETP R, FEP R, GEN R, IMP R, MEM R, MIN S, SAM S, TOB S, TZP R |
| SPBC-2128 | Escherichia coli | Enterobacterales | Seeded Clinical | AMC S, AMK S, AMP R, CAZ S, CFZ I, CIP R, CRO R, CZA S, ETP R, FEP R, GEN R, IMP R, MEM R, MIN S, SAM I, TOB I, TZP S |
| SPBC-1005 | Escherichia coli | Enterobacterales | Seeded Clinical | AMC S, AMK R, AMP S, CAZ S, CFZ S, CIP R, CRO R, CZA S, ETP R, FEP R, GEN R, IMP R, MEM R, MIN S, SAM S, TOB R, TZP R |
| 5026 | Escherichia coli | Enterobacterales | Challenge | AMC I, AMK S, AMP R, CAZ R, CIP R, CRO R, CZA R, ETP R, FEP R, GEN R, IMP R, MEM R, MIN S, SAM R, TOB R, TZP S |
| 5038 | Escherichia coli | Enterobacterales | Challenge | AMC S, AMK S, AMP R, CAZ R, CIP S, CRO R, CZA S, ETP R, FEP R, GEN R, IMP R, MEM R, MIN S, SAM R, TOB S, TZP S |
| 5040 | Escherichia coli | Enterobacterales | Challenge | AMC S, AMK S, AMP S, CAZ S, CIP R, CRO R, CZA R, ETP R, FEP S, GEN R, IMP R, MEM R, MIN S, SAM S, TOB S, TZP R |
| 5044 | Escherichia coli | Enterobacterales | Challenge | AMC S, AMK S, AMP R, CAZ S, CIP S, CRO R, CZA R, ETP R, FEP R, GEN R, IMP R, MEM R, MIN S, SAM I, TOB I, TZP S |
| PBC-1002 | Escherichia coli | Enterobacterales | Fresh Clinical | AMC S, CIP R, GEN R, SAM S |
| PBC-1003 | Escherichia coli | Enterobacterales | Fresh Clinical | AMC S, CIP R, GEN R, SAM S |
| PBC-1004 | Escherichia coli | Enterobacterales | Fresh Clinical | AMC S, CIP R, GEN R, SAM I |
| PBC-1005 | Escherichia coli | Enterobacterales | Fresh Clinical | AMC S, CIP R, GEN R, SAM S |
| PBC-1008 | Escherichia coli | Enterobacterales | Fresh Clinical | AMC I, CIP R, GEN R, SAM I |
| PBC-1009 | Escherichia coli | Enterobacterales | Fresh Clinical | AMC S, CIP R, GEN R, SAM S |
| PBC-1010 | Escherichia coli | Enterobacterales | Fresh Clinical | AMC S, CIP R, GEN R, SAM S |
| PBC-1012 | Escherichia coli | Enterobacterales | Fresh Clinical | AMC S, CIP R, GEN S, SAM I |
| PBC-1014 | Escherichia coli | Enterobacterales | Fresh Clinical | AMC S, CIP R, GEN S, SAM S |
| PBC-1016 | Escherichia coli | Enterobacterales | Fresh Clinical | AMC S, CIP R, GEN R, SAM S |
| PBC-1017 | Escherichia coli | Enterobacterales | Fresh Clinical | AMC S, CIP R, GEN R, SAM S |
| PBC-1018 | Escherichia coli | Enterobacterales | Fresh Clinical | AMC S, CIP R, GEN R, SAM I |
| PBC-1025 | Escherichia coli | Enterobacterales | Fresh Clinical | AMC I, CIP R, GEN R, SAM R |
| PBC-1029 | Escherichia coli | Enterobacterales | Fresh Clinical | AMC S, CIP R, GEN R, SAM I |
| PBC-1030 | Escherichia coli | Enterobacterales | Fresh Clinical | AMC S, CIP S, GEN R, SAM I |
| PBC-1032 | Escherichia coli | Enterobacterales | Fresh Clinical | AMC S, CIP S, GEN S, SAM I |
| PBC-1033 | Escherichia coli | Enterobacterales | Fresh Clinical | AMC S, CIP S, GEN R, SAM S |
| PBC-1034 | Escherichia coli | Enterobacterales | Fresh Clinical | AMC S, CIP R, GEN R, SAM I |
| PBC-1103 | Escherichia coli | Enterobacterales | Fresh Clinical | AMC S, AMK S, AMP R, CAZ R, CFZ R, CIP R, CRO R, CZA S, ETP R, FEP R, GEN R, IMP R, MEM R, MIN S, SAM I, TOB R, TZP I |
| PBC-1104 | Escherichia coli | Enterobacterales | Fresh Clinical | AMC S, AMK S, AMP S, CAZ S, CFZ S, CIP S, CRO R, CZA S, ETP R, FEP S, GEN R, IMP R, MEM R, MIN S, SAM S, TOB S, TZP S |
| PBC-1105 | Escherichia coli | Enterobacterales | Fresh Clinical | AMC S, AMK S, AMP S, CAZ S, CFZ S, CIP R, CRO R, CZA R, ETP R, FEP S, GEN R, IMP R, MEM R, MIN S, SAM S, TOB S, TZP R |
| PBC-1109 | Escherichia coli | Enterobacterales | Fresh Clinical | AMC S, AMK S, AMP S, CAZ S, CFZ S, CIP R, CRO R, CZA R, ETP R, FEP R, GEN R, IMP R, MEM R, MIN S, SAM S, TOB S, TZP S |
| PBC-1110 | Escherichia coli | Enterobacterales | Fresh Clinical | AMC S, AMK S, AMP R, CAZ S, CFZ S, CIP S, CRO R, CZA S, ETP R, FEP R, GEN R, IMP R, MEM R, MIN S, SAM I, TOB I, TZP S |
| PBC-1111 | Escherichia coli | Enterobacterales | Fresh Clinical | AMC S, AMK S, AMP R, CAZ S, CFZ I, CIP S, CRO R, CZA S, ETP R, FEP R, GEN R, IMP R, MEM R, MIN S, SAM I, TOB I, TZP R |
| PBC-1115 | Escherichia coli | Enterobacterales | Fresh Clinical | AMC S, AMK S, AMP R, CAZ S, CFZ R, CIP R, CRO R, CZA R, ETP R, FEP S, GEN R, IMP R, MEM R, MIN S, SAM S, TOB S, TZP S |
| PBC-1117 | Escherichia coli | Enterobacterales | Fresh Clinical | AMC S, AMK S, AMP R, CAZ S, CFZ S, CIP R, CRO R, CZA S, ETP R, FEP R, GEN S, IMP R, MEM R, MIN S, SAM I, TOB S, TZP S |
| PBC-1121 | Escherichia coli | Enterobacterales | Fresh Clinical | AMC S, AMK S, AMP R, CAZ S, CFZ S, CIP R, CRO R, CZA R, ETP R, FEP R, GEN R, IMP S, MEM R, MIN S, SAM I, TOB I, TZP S |
| PBC-1122 | Escherichia coli | Enterobacterales | Fresh Clinical | AMC S, AMK S, AMP R, CAZ R, CFZ R, CIP R, CRO R, CZA S, ETP S, FEP R, GEN R, IMP R, MEM R, MIN R, SAM I, TOB I, TZP S |
| PBC-1124 | Escherichia coli | Enterobacterales | Fresh Clinical | AMC S, AMK S, AMP S, CAZ S, CFZ S, CIP R, CRO R, CZA R, ETP R, FEP R, GEN S, IMP R, MEM R, MIN R, SAM S, TOB S, TZP S |
| PBC-1127 | Escherichia coli | Enterobacterales | Fresh Clinical | AMC S, AMK S, AMP R, CAZ S, CFZ I, CIP R, CRO R, CZA S, ETP R, FEP S, GEN S, IMP R, MEM R, MIN S, SAM R, TOB S, TZP S |
| PBC-1128 | Escherichia coli | Enterobacterales | Fresh Clinical | AMC S, AMK S, AMP R, CAZ R, CFZ R, CIP R, CRO R, CZA R, ETP R, FEP R, GEN R, IMP R, MEM R, MIN S, SAM R, TOB R, TZP S |
| PBC-1129 | Escherichia coli | Enterobacterales | Fresh Clinical | AMC S, AMK S, AMP S, CAZ S, CFZ S, CIP S, CRO R, CZA R, ETP R, FEP R, GEN S, IMP R, MEM R, MIN S, SAM S, TOB S, TZP S |
| PBC-1133 | Escherichia coli | Enterobacterales | Fresh Clinical | AMC S, AMK S, AMP R, CAZ S, CFZ I, CIP R, CRO R, CZA R, ETP R, FEP R, GEN R, IMP R, MEM R, MIN I, SAM I, TOB S, TZP S |
| PBC-1135 | Escherichia coli | Enterobacterales | Fresh Clinical | AMC S, AMK S, AMP R, CAZ I, CFZ R, CIP S, CRO R, CZA R, ETP R, FEP SDD, GEN R, IMP R, MEM R, MIN S, SAM R, TOB R, TZP S |
| PBC-1136 | Escherichia coli | Enterobacterales | Fresh Clinical | AMC S, AMK S, AMP S, CAZ S, CFZ S, CIP S, CRO R, CZA R, ETP R, FEP R, GEN S, IMP R, MEM R, MIN S, SAM S, TOB S, TZP S |
| PBC-1153 | Escherichia coli | Enterobacterales | Fresh Clinical | AMC S, AMK S, AMP R, CAZ S, CFZ I, CIP R, CRO R, CZA R, ETP R, FEP R, GEN S, IMP R, MEM R, MIN R, SAM R, TOB S, TZP S |
| PBC-1157 | Escherichia coli | Enterobacterales | Fresh Clinical | AMC S, AMK S, AMP R, CAZ I, CFZ R, CIP R, CRO R, CZA S, ETP S, FEP R, GEN R, IMP S, MEM R, MIN S, SAM R, TOB R, TZP S |
| PBC-1158 | Escherichia coli | Enterobacterales | Fresh Clinical | AMC S, AMK S, AMP R, CAZ I, CFZ R, CIP R, CRO R, CZA R, ETP R, FEP R, GEN R, IMP R, MEM R, MIN S, SAM R, TOB R, TZP S |
| PBC-1160 | Escherichia coli | Enterobacterales | Fresh Clinical | AMC S, AMK S, AMP R, CAZ R, CFZ I, CIP R, CRO R, CZA S, ETP R, FEP R, GEN R, IMP R, MEM R, MIN S, SAM I, TOB S, TZP S |
| PBC-1161 | Escherichia coli | Enterobacterales | Fresh Clinical | AMC S, AMK S, AMP R, CAZ S, CIP R, CRO R, CZA S, ETP R, FEP R, GEN R, IMP R, MEM R, MIN R, SAM I, TOB S, TZP S |
| PBC-1163 | Escherichia coli | Enterobacterales | Fresh Clinical | AMC S, AMK S, AMP R, CAZ R, CFZ R, CIP R, CRO R, CZA S, ETP S, FEP R, GEN R, IMP R, MEM R, MIN I, SAM R, TOB S, TZP S |
| PBC-1164 | Escherichia coli | Enterobacterales | Fresh Clinical | AMC I, AMK R, AMP R, CAZ S, CFZ R, CIP R, CRO R, CZA S, ETP R, FEP R, GEN R, IMP R, MEM R, MIN R, SAM R, TOB S, TZP S |
| PBC-1165 | Escherichia coli | Enterobacterales | Fresh Clinical | AMC S, AMK S, AMP S, CAZ S, CFZ S, CIP R, CRO R, CZA S, ETP R, FEP R, GEN R, IMP R, MEM R, MIN S, SAM S, TOB S, TZP R |
| PBC-1166 | Escherichia coli | Enterobacterales | Fresh Clinical | AMC S, AMK S, AMP S, CAZ S, CFZ S, CIP R, CRO R, CZA R, ETP R, FEP R, GEN R, IMP R, MEM R, MIN R, SAM S, TOB S, TZP S |
| PBC-1167 | Escherichia coli | Enterobacterales | Fresh Clinical | AMC S, AMK S, AMP S, CAZ S, CFZ S, CIP R, CRO R, CZA R, ETP R, FEP R, GEN S, IMP R, MEM R, MIN S, SAM S, TOB S, TZP S |
| PBC-1168 | Escherichia coli | Enterobacterales | Fresh Clinical | AMC S, AMK S, AMP S, CAZ S, CFZ S, CIP R, CRO R, CZA R, ETP R, FEP R, GEN R, IMP R, MEM R, MIN I, SAM S, TOB S, TZP S |
| PBC-1170 | Escherichia coli | Enterobacterales | Fresh Clinical | AMC I, AMK S, AMP R, CAZ R, CFZ R, CIP I, CRO R, CZA S, ETP R, FEP R, GEN R, IMP R, MEM R, MIN I, SAM R, TOB R, TZP R |
| PBC-1171 | Escherichia coli | Enterobacterales | Fresh Clinical | AMC I, AMK S, AMP R, CAZ S, CFZ R, CIP R, CRO R, CZA S, ETP R, FEP R, GEN R, IMP R, MEM R, MIN S, SAM I, TOB S, TZP S |
| PBC-1999 | Escherichia coli | Enterobacterales | Fresh Clinical | AMC S, AMK S, AMP R, CAZ R, CFZ I, CIP R, CRO R, CZA S, ETP R, FEP S, GEN R, IMP R, MEM R, MIN S, SAM S, TOB S, TZP S |
| PBC-2001 | Escherichia coli | Enterobacterales | Fresh Clinical | AMC S, CIP R, GEN S, SAM R |
| PBC-2106 | Escherichia coli | Enterobacterales | Fresh Clinical | AMC I, AMK S, AMP R, CAZ S, CFZ R, CIP R, CRO R, CZA S, ETP R, FEP R, GEN R, IMP R, MEM R, MIN S, SAM R, TOB I, TZP S |
| PBC-2108 | Escherichia coli | Enterobacterales | Fresh Clinical | AMC S, AMK S, AMP S, CAZ S, CFZ S, CIP R, CRO R, CZA S, ETP R, FEP R, GEN R, IMP R, MEM R, MIN S, SAM S, TOB S, TZP S |
| PBC-2111 | Escherichia coli | Enterobacterales | Fresh Clinical | AMC S, AMK S, AMP R, CAZ S, CFZ S, CIP S, CRO R, CZA S, ETP R, FEP R, GEN R, IMP R, MEM R, MIN S, SAM I, TOB S, TZP S |
| PBC-2112 | Escherichia coli | Enterobacterales | Fresh Clinical | AMC S, AMK S, AMP R, CAZ I, CFZ R, CIP R, CRO R, CZA S, ETP R, FEP R, GEN R, IMP R, MEM R, MIN S, SAM R, TOB I, TZP S |
| PBC-2113 | Escherichia coli | Enterobacterales | Fresh Clinical | AMC S, AMK S, AMP R, CAZ S, CIP R, CRO R, CZA S, ETP R, FEP R, GEN R, IMP R, MEM R, MIN S, SAM I, TOB S, TZP S |
| PBC-2114 | Escherichia coli | Enterobacterales | Fresh Clinical | AMC S, AMK S, AMP R, CAZ S, CFZ S, CIP R, CRO R, CZA S, ETP R, FEP R, GEN R, IMP R, MEM R, MIN S, SAM I, TOB S, TZP S |
| PBC-2116 | Escherichia coli | Enterobacterales | Fresh Clinical | AMC S, AMK S, AMP R, CAZ S, CFZ S, CIP S, CRO R, CZA S, ETP R, FEP R, GEN R, IMP R, MEM R, MIN S, SAM R, TOB S, TZP S |
| PBC-2117 | Escherichia coli | Enterobacterales | Fresh Clinical | AMC S, AMK S, AMP S, CAZ R, CFZ S, CIP R, CRO R, CZA R, ETP R, FEP R, GEN S, IMP R, MEM R, MIN S, SAM S, TOB S, TZP S |
| PBC-2118 | Escherichia coli | Enterobacterales | Fresh Clinical | AMC I, AMK S, AMP R, CAZ S, CFZ S, CIP R, CRO R, CZA S, ETP R, FEP S, GEN R, IMP R, MEM R, MIN S, SAM I, TOB I, TZP S |
| PBC-2120 | Escherichia coli | Enterobacterales | Fresh Clinical | AMC S, AMK S, AMP S, CAZ S, CFZ S, CIP R, CRO R, CZA R, ETP R, FEP R, GEN S, IMP R, MEM R, MIN S, SAM S, TOB S, TZP S |
| PBC-2121 | Escherichia coli | Enterobacterales | Fresh Clinical | AMC S, AMK S, AMP S, CAZ S, CFZ S, CIP R, CRO R, CZA R, ETP R, FEP R, GEN R, IMP R, MEM S, MIN S, SAM S, TOB S, TZP S |
| PBC-2123 | Escherichia coli | Enterobacterales | Fresh Clinical | AMC S, AMK S, AMP S, CAZ S, CFZ S, CIP S, CRO R, CZA R, ETP R, FEP R, IMP R, MEM R, MIN S, SAM S, TOB S, TZP S |
| PBC-3001 | Escherichia coli | Enterobacterales | Site 2 | AMC S, CIP R, GEN R, SAM S |
| PBC-3100 | Escherichia coli | Enterobacterales | Fresh Clinical | AMC I, AMK S, AMP R, CAZ S, CFZ R, CIP R, CRO R, CZA R, ETP R, FEP R, GEN R, IMP R, MEM R, MIN S, SAM R, TOB S, TZP S |
| PBC-3102 | Escherichia coli | Enterobacterales | Fresh Clinical | AMC S, AMK S, AMP S, CAZ S, CFZ S, CIP I, CRO R, CZA S, ETP R, FEP S, GEN R, IMP R, MEM R, MIN R, SAM S, TOB S, TZP R |
| PBC-3103 | Escherichia coli | Enterobacterales | Fresh Clinical | AMC S, AMK S, AMP R, CAZ S, CFZ R, CIP R, CRO R, CZA R, ETP R, FEP R, GEN R, IMP R, MEM R, MIN S, SAM R, TOB S, TZP S |
| PBC-3104 | Escherichia coli | Enterobacterales | Fresh Clinical | AMC S, AMK S, AMP R, CAZ R, CFZ I, CIP S, CRO R, CZA S, ETP R, FEP R, GEN R, IMP R, MEM R, MIN S, SAM I, TOB I, TZP S |
| PBC-3105 | Escherichia coli | Enterobacterales | Fresh Clinical | AMC S, AMK S, AMP R, CAZ R, CFZ I, CIP S, CRO R, CZA S, ETP R, FEP R, GEN R, IMP R, MEM R, MIN S, SAM I, TOB I, TZP S |
| PBC-3106 | Escherichia coli | Enterobacterales | Fresh Clinical | AMC S, AMK S, AMP R, CAZ S, CFZ S, CIP I, CRO R, CZA S, ETP R, FEP R, GEN R, IMP R, MEM R, MIN R, SAM I, TOB S, TZP S |
| PBC-3107 | Escherichia coli | Enterobacterales | Fresh Clinical | AMC I, AMK S, AMP R, CAZ S, CIP R, CRO R, CZA S, ETP R, FEP S, GEN R, IMP R, MEM R, MIN S, SAM I, TOB S, TZP S |
| PBC-3109 | Escherichia coli | Enterobacterales | Fresh Clinical | AMC S, AMK S, AMP R, CAZ S, CFZ I, CIP R, CRO R, CZA S, ETP R, FEP R, GEN R, IMP R, MEM R, MIN S, SAM I, TOB S, TZP S |
| PBC-3110 | Escherichia coli | Enterobacterales | Fresh Clinical | AMC S, AMK S, AMP S, CAZ R, CFZ S, CIP R, CRO R, CZA S, ETP R, FEP R, GEN R, IMP R, MEM R, MIN S, SAM S, TOB S, TZP S |
| PBC-3111 | Escherichia coli | Enterobacterales | Fresh Clinical | AMC I, AMK S, AMP R, CAZ S, CFZ S, CIP R, CRO R, CZA S, ETP R, FEP R, GEN S, IMP R, MEM R, MIN S, SAM R, TOB S, TZP S |
| PBC-3119 | Escherichia coli | Enterobacterales | Fresh Clinical | AMC S, AMK S, AMP R, CAZ R, CFZ I, CIP R, CRO R, CZA R, ETP R, FEP R, GEN R, IMP R, MEM R, MIN S, SAM I, TOB S, TZP S |
| PBC-3120 | Escherichia coli | Enterobacterales | Fresh Clinical | AMC S, AMK S, AMP R, CAZ S, CFZ R, CIP R, CRO R, CZA S, ETP R, FEP S, GEN R, IMP R, MEM R, MIN S, SAM S, TOB S, TZP S |
| PBC-4109 | Escherichia coli | Enterobacterales | Fresh Clinical | AMC S, AMK S, AMP S, CAZ S, CFZ S, CIP R, CRO R, CZA S, ETP R, FEP R, GEN S, IMP R, MEM R, MIN S, SAM S, TOB S, TZP S |
| PBC-4110 | Escherichia coli | Enterobacterales | Fresh Clinical | AMC S, AMK S, AMP R, CAZ R, CFZ S, CIP S, CRO R, CZA R, ETP R, FEP R, GEN R, IMP R, MEM R, MIN S, SAM S, TOB S, TZP S |
| PBC-4114 | Escherichia coli | Enterobacterales | Fresh Clinical | AMC S, AMK S, AMP R, CAZ S, CFZ R, CIP R, CRO R, CZA S, ETP R, FEP S, GEN S, IMP R, MEM R, MIN S, SAM S, TOB S, TZP S |
| PBC-4121 | Escherichia coli | Enterobacterales | Fresh Clinical | AMC S, AMK S, AMP R, CAZ R, CFZ R, CIP R, CRO R, CZA S, ETP S, FEP R, GEN S, IMP S, MEM R, MIN S, SAM I, TOB S, TZP S |
| PBC-4124 | Escherichia coli | Enterobacterales | Fresh Clinical | AMC S, AMK S, AMP R, CAZ I, CFZ R, CIP R, CRO R, CZA R, ETP S, FEP R, GEN S, IMP R, MEM R, MIN S, SAM I, TOB S, TZP S |
| PBC-4125 | Escherichia coli | Enterobacterales | Fresh Clinical | AMC S, AMK S, AMP R, CAZ I, CFZ R, CIP R, CRO R, CZA S, ETP S, FEP R, GEN S, IMP R, MEM R, MIN S, SAM I, TOB S, TZP R |
| PBC-4126 | Escherichia coli | Enterobacterales | Fresh Clinical | AMC S, AMK S, AMP R, CAZ I, CFZ R, CIP R, CRO R, CZA R, ETP R, FEP R, GEN R, IMP S, MEM R, MIN S, SAM R, TOB R, TZP S |
| PBC-4127 | Escherichia coli | Enterobacterales | Fresh Clinical | AMC S, AMK S, AMP R, CAZ R, CFZ R, CIP R, CRO R, CZA S, ETP R, FEP R, GEN R, IMP R, MEM R, MIN S, SAM I, TOB S, TZP S |
| PBC-4129 | Escherichia coli | Enterobacterales | Fresh Clinical | AMC R, AMK S, AMP R, CAZ R, CFZ R, CIP R, CRO R, CZA R, ETP R, FEP R, GEN R, IMP R, MEM R, MIN S, SAM R, TOB I |
| PBC-4130 | Escherichia coli | Enterobacterales | Fresh Clinical | AMC S, AMK S, AMP R, CAZ R, CFZ R, CIP R, CRO R, CZA S, FEP R, GEN R, IMP S, MEM R, MIN S, SAM I, TOB S, TZP S |
| PBC-4142 | Escherichia coli | Enterobacterales | Fresh Clinical | AMC S, AMK S, AMP R, CAZ R, CFZ R, CIP R, CRO R, CZA R, ETP S, FEP R, GEN R, IMP R, MEM R, MIN S, SAM R, TOB R, TZP S |
| PBC-4147 | Escherichia coli | Enterobacterales | Fresh Clinical | AMC S, AMK S, AMP R, CAZ S, CFZ S, CIP R, CRO R, CZA S, ETP R, FEP R, GEN R, IMP R, MEM R, MIN S, SAM I, TOB S, TZP S |
| PBC-4149 | Escherichia coli | Enterobacterales | Fresh Clinical | AMC S, AMK S, AMP S, CAZ S, CFZ S, CIP R, CRO R, CZA S, ETP R, FEP R, GEN R, IMP R, MEM R, MIN S, SAM S, TOB S, TZP R |
| PBC-4152 | Escherichia coli | Enterobacterales | Fresh Clinical | AMC S, AMK S, AMP R, CAZ R, CFZ S, CIP R, CRO R, CZA S, ETP R, FEP R, GEN R, IMP R, MEM R, MIN S, SAM S, TOB S, TZP S |
| SD-1066 | Escherichia coli | Enterobacterales | Challenge | AMC R, AMK S, AMP R, CAZ R, CFZ R, CIP R, CRO R, CZA S, ETP S, FEP S, GEN R, IMP R, MEM R, MIN S, SAM R, TOB S, TZP S |
| SD-1069 | Escherichia coli | Enterobacterales | Challenge | AMC S, AMK S, AMP R, CAZ R, CFZ R, CIP R, CRO R, CZA S, ETP R, FEP R, GEN S, IMP R, MEM R, MIN S, SAM R, TOB I, TZP S |
| SD-1896 | Escherichia coli | Enterobacterales | Challenge | AMC R, AMK S, AMP R, CAZ R, CFZ R, CIP R, CRO R, CZA S, ETP S, FEP R, GEN R, IMP R, MEM R, MIN I, SAM R, TOB S, TZP R |
| SD-2131 | Escherichia coli | Enterobacterales | Challenge | AMC S, AMK S, AMP R, CAZ R, CFZ I, CIP R, CRO R, CZA R, ETP R, FEP R, GEN R, IMP R, MEM R, MIN S, SAM R, TOB S, TZP S |
| SD-308 | Escherichia coli | Enterobacterales | Challenge | AMC R, AMK S, AMP R, CAZ R, CFZ R, CIP R, CRO R, CZA S, ETP R, FEP R, GEN S, IMP R, MIN I, SAM R, TOB R, TZP R |
| SD-310 | Escherichia coli | Enterobacterales | Challenge | AMC S, AMK S, AMP R, CAZ S, CFZ R, CIP S, CRO R, CZA R, ETP R, FEP SDD, GEN R, IMP R, MEM R, MIN S, SAM I, TOB I, TZP R |
| SD-311 | Escherichia coli | Enterobacterales | Challenge | AMC I, AMK S, AMP R, CAZ R, CFZ R, CIP R, CRO R, CZA R, ETP R, FEP R, GEN R, IMP R, MEM R, MIN S, SAM R, TOB R, TZP S |
| SD-312 | Escherichia coli | Enterobacterales | Challenge | AMC R, AMK S, AMP R, CAZ R, CFZ R, CIP R, CRO R, CZA S, FEP R, GEN R, IMP R, MEM R, MIN I, SAM R, TOB R, TZP R |
| SD-315 | Escherichia coli | Enterobacterales | Challenge | AMC R, AMK R, AMP R, CAZ R, CFZ R, CIP R, CRO R, CZA S, ETP S, FEP R, GEN R, IMP R, MEM R, MIN I, SAM R, TOB R, TZP R |
| SD-317 | Escherichia coli | Enterobacterales | Challenge | AMC S, AMK S, AMP R, CAZ S, CFZ R, CIP R, CRO R, CZA S, ETP S, GEN R, IMP R, MEM R, MIN S, SAM I, TOB S, TZP R |
| SD-318 | Escherichia coli | Enterobacterales | Challenge | AMC S, AMK S, AMP R, CAZ R, CFZ R, CIP R, CRO R, CZA R, ETP S, FEP R, GEN I, IMP R, MEM R, MIN S, SAM R, TOB R, TZP R |
| SD-5219 | Escherichia coli | Enterobacterales | Challenge | AMC S, AMK S, AMP R, CAZ S, CFZ I, CIP R, CRO R, CZA S, ETP R, FEP R, GEN R, IMP R, MEM R, MIN S, SAM S, TOB S, TZP R |
| SD-5403 | Escherichia coli | Enterobacterales | Challenge | AMC S, AMK S, AMP R, CAZ I, CFZ R, CIP R, CRO R, CZA R, ETP R, GEN R, IMP R, MEM R, MIN I, SAM I, TOB S, TZP S |
| SD-5406 | Escherichia coli | Enterobacterales | Challenge | AMC S, AMK S, AMP R, CAZ S, CFZ R, CIP S, CRO R, CZA S, ETP R, FEP S, GEN R, IMP R, MEM R, MIN S, SAM R, TOB S, TZP S |
| SD-5408 | Escherichia coli | Enterobacterales | Challenge | AMC S, AMK S, AMP R, CAZ I, CFZ R, CIP R, CRO R, CZA R, ETP R, GEN R, IMP R, MEM R, MIN S, SAM I, TOB I, TZP R |
| SD-5410 | Escherichia coli | Enterobacterales | Challenge | AMC S, AMK S, AMP R, CAZ R, CFZ R, CIP R, CRO R, CZA R, ETP R, FEP R, GEN R, IMP R, MEM R, MIN S, SAM R, TOB I, TZP S |
| SD-5412 | Escherichia coli | Enterobacterales | Challenge | AMC I, AMK S, AMP R, CAZ S, CFZ R, CIP R, CRO R, CZA S, ETP R, GEN R, IMP R, MEM S, MIN R, SAM R, TOB S, TZP S |
| SD-5415 | Escherichia coli | Enterobacterales | Challenge | AMC S, AMK S, AMP R, CAZ S, CFZ R, CIP R, CRO R, CZA R, ETP R, GEN I, IMP R, MEM R, MIN S, SAM I, TZP S |
| SD-5417 | Escherichia coli | Enterobacterales | Challenge | AMC S, AMK S, AMP R, CAZ S, CFZ R, CIP R, CRO R, CZA R, ETP R, GEN R, IMP R, MEM R, MIN S, SAM R, TOB I, TZP S |
| SD-5419 | Escherichia coli | Enterobacterales | Challenge | AMC S, AMK S, AMP R, CAZ S, CFZ R, CIP R, CRO R, CZA S, ETP S, GEN R, IMP R, MEM R, MIN S, SAM R, TOB I, TZP S |
| SD-5423 | Escherichia coli | Enterobacterales | Challenge | AMC S, AMK S, AMP R, CAZ S, CFZ R, CIP R, CRO R, CZA S, ETP R, FEP SDD, GEN S, IMP R, MEM R, MIN S, SAM S, TOB S, TZP S |
| SD-5425 | Escherichia coli | Enterobacterales | Challenge | AMC S, AMK S, AMP R, CAZ R, CFZ R, CIP S, CRO R, CZA S, ETP S, FEP R, GEN R, IMP R, MEM R, MIN S, SAM R, TOB S, TZP S |
| SD-5426 | Escherichia coli | Enterobacterales | Challenge | AMC S, AMK S, AMP R, CAZ R, CFZ R, CIP R, CRO R, CZA S, ETP R, FEP S, GEN R, IMP R, MEM R, MIN R, SAM R, TOB S, TZP S |
| SD-5427 | Escherichia coli | Enterobacterales | Challenge | AMC S, AMK S, AMP R, CAZ I, CFZ R, CIP R, CRO R, CZA R, ETP R, GEN I, IMP R, MEM R, MIN S, SAM R, TOB I |
| SPBC-2015 | Klebsiella oxytoca | Enterobacterales | Seeded Clinical | AMC S, CIP R, CRO S, CZA S, ETP R, FEP S, GEN R, MEM S, SAM R |
| SPBC-2016 | Klebsiella oxytoca | Enterobacterales | Seeded Clinical | AMC S, CIP R, CRO S, CZA S, ETP S, FEP S, GEN R, MEM S, SAM S |
| SPBC-2017 | Klebsiella oxytoca | Enterobacterales | Seeded Clinical | AMC S, CIP R, CRO S, CZA S, ETP R, FEP S, GEN R, MEM S, SAM S |
| SPBC-2018 | Klebsiella oxytoca | Enterobacterales | Seeded Clinical | AMC R, CIP S, CRO R, CZA S, ETP R, GEN R, MEM S, SAM R |
| SPBC-2019 | Klebsiella oxytoca | Enterobacterales | Seeded Clinical | AMC S, CIP R, CRO S, CZA S, ETP R, FEP S, GEN R, MEM S, SAM S |
| SPBC-2020 | Klebsiella oxytoca | Enterobacterales | Seeded Clinical | AMC S, CIP S, CRO R, CZA S, ETP R, FEP S, GEN R, MEM S, SAM S |
| SPBC-2090 | Klebsiella oxytoca | Enterobacterales | Seeded Clinical | AMC S, CIP R, CRO S, CZA S, ETP S, FEP S, GEN R, MEM S, SAM S |
| SPBC-2095 | Klebsiella oxytoca | Enterobacterales | Seeded Clinical | AMC R, CIP S, CRO R, CZA S, ETP S, FEP SDD, GEN R, MEM S, SAM R |
| SPBC-2096 | Klebsiella oxytoca | Enterobacterales | Seeded Clinical | AMC S, CIP R, CRO S, CZA S, ETP R, FEP S, GEN R, MEM S, SAM S |
| SPBC-2097 | Klebsiella oxytoca | Enterobacterales | Seeded Clinical | AMC S, CIP R, CRO S, CZA S, ETP R, FEP S, GEN R, MEM S, SAM S |
| SPBC-2101 | Klebsiella oxytoca | Enterobacterales | Seeded Clinical | AMC S, CIP S, CRO S, CZA S, ETP R, FEP S, GEN R, MEM S, SAM S |
| SPBC-2102 | Klebsiella oxytoca | Enterobacterales | Seeded Clinical | AMC S, CIP S, CRO S, CZA S, ETP S, FEP S, GEN R, MEM S, SAM S |
| SPBC-2115 | Klebsiella oxytoca | Enterobacterales | Seeded Clinical | AMC S, CIP R, CRO S, CZA S, ETP R, FEP S, GEN R, MEM S, SAM S |
| SPBC-2134 | Klebsiella oxytoca | Enterobacterales | Seeded Clinical | AMC S, CIP R, CRO S, CZA S, ETP R, FEP S, GEN R, MEM S, SAM S |
| SPBC-3064 | Klebsiella oxytoca | Enterobacterales | Seeded Clinical | AMC S, CIP R, CRO S, CZA S, ETP S, FEP S, GEN R, MEM S, SAM S |
| SPBC-3065 | Klebsiella oxytoca | Enterobacterales | Seeded Clinical | AMC S, CIP R, CRO S, CZA S, ETP S, FEP S, GEN R, MEM S, SAM S |
| SPBC-3066 | Klebsiella oxytoca | Enterobacterales | Seeded Clinical | AMC S, CIP R, CRO S, CZA S, ETP R, FEP S, GEN R, MEM S, SAM R |
| SPBC-3067 | Klebsiella oxytoca | Enterobacterales | Seeded Clinical | AMC R, CIP R, CRO R, CZA S, ETP S, FEP R, GEN R, MEM S, SAM R |
| PBC-4116 | Klebsiella oxytoca | Enterobacterales | Fresh Clinical | AMC S, CIP R, CRO S, CZA S, ETP R, FEP S, GEN R, MEM S, SAM S |
| PBC-4131 | Klebsiella oxytoca | Enterobacterales | Fresh Clinical | AMC S, CIP R, CRO S, CZA S, ETP S, FEP S, GEN R, MEM S, SAM S |
| PBC-4137 | Klebsiella oxytoca | Enterobacterales | Fresh Clinical | AMC S, CIP R, CRO S, CZA S, ETP R, FEP S, GEN R, MEM S, SAM S |
| SPBC-3004 | Klebsiella pneumoniae | Enterobacterales | Seeded Clinical | AMC S, CIP R, GEN R, SAM S |
| SPBC-3005 | Klebsiella pneumoniae | Enterobacterales | Seeded Clinical | AMC S, AMK R, CAZ S, CFZ S, CIP R, CRO S, CZA S, ETP S, FEP S, GEN R, IMP R, MEM S, MIN S, SAM S, TOB S, TZP S |
| SPBC-2006 | Klebsiella pneumoniae | Enterobacterales | Seeded Clinical | AMC S, AMK R, CAZ S, CFZ S, CIP R, CRO S, CZA S, ETP S, FEP S, GEN R, IMP S, MEM S, MIN S, SAM S, TOB S, TZP S |
| SPBC-3006 | Klebsiella pneumoniae | Enterobacterales | Seeded Clinical | AMC S, AMK R, CAZ R, CFZ R, CIP R, CRO R, CZA S, ETP S, FEP R, GEN R, IMP R, MEM S, MIN R, SAM R, TOB S, TZP R |
| SPBC-2007 | Klebsiella pneumoniae | Enterobacterales | Seeded Clinical | AMC R, AMK R, CAZ R, CFZ R, CIP S, CRO R, CZA S, ETP R, FEP R, GEN R, IMP R, MEM R, MIN R, SAM R, TOB S, TZP R |
| SPBC-1013 | Klebsiella pneumoniae | Enterobacterales | Seeded Clinical | AMC R, AMK R, CAZ R, CFZ R, CIP R, CRO R, CZA S, ETP R, FEP SDD, GEN R, IMP R, MEM R, MIN S, SAM R, TOB S, TZP R |
| SPBC-1010 | Klebsiella pneumoniae | Enterobacterales | Seeded Clinical | AMC R, AMK S, CAZ R, CFZ R, CIP R, CRO I, ETP R, FEP R, GEN R, IMP R, MEM R, MIN S, SAM R, TOB S, TZP R |
| SPBC-2008 | Klebsiella pneumoniae | Enterobacterales | Seeded Clinical | AMC R, CIP R, GEN R, SAM R |
| SPBC-2009 | Klebsiella pneumoniae | Enterobacterales | Seeded Clinical | AMC R, AMK S, CAZ R, CFZ R, CIP R, CRO R, CZA S, ETP R, FEP R, GEN R, IMP R, MEM R, MIN R, SAM R, TOB R, TZP R |
| SPBC-2010 | Klebsiella pneumoniae | Enterobacterales | Seeded Clinical | AMC R, CIP R, GEN S, SAM R |
| SPBC-3007 | Klebsiella pneumoniae | Enterobacterales | Seeded Clinical | AMC R, AMK R, CAZ R, CFZ R, CIP R, CRO R, CZA S, ETP R, FEP R, GEN R, IMP R, MEM R, MIN S, SAM R, TOB S, TZP R |
| SPBC-3008 | Klebsiella pneumoniae | Enterobacterales | Seeded Clinical | AMC S, AMK R, CAZ R, CFZ R, CIP R, CRO R, CZA S, ETP S, FEP SDD, GEN R, IMP R, MEM S, MIN S, SAM R, TOB S, TZP R |
| SPBC-3009 | Klebsiella pneumoniae | Enterobacterales | Seeded Clinical | AMC S, AMK I, CAZ R, CFZ R, CIP R, CRO R, CZA S, ETP S, FEP R, GEN S, IMP R, MEM S, MIN R, SAM R, TOB R, TZP R |
| SPBC-2011 | Klebsiella pneumoniae | Enterobacterales | Seeded Clinical | AMC I, AMK R, CAZ R, CFZ R, CIP R, CRO R, CZA S, ETP S, FEP R, GEN R, IMP R, MEM S, MIN R, SAM R, TOB S, TZP R |
| SPBC-3010 | Klebsiella pneumoniae | Enterobacterales | Seeded Clinical | AMC S, AMK R, CAZ S, CFZ S, CIP R, CRO S, CZA S, ETP S, FEP S, GEN R, IMP R, MEM S, MIN S, SAM S, TOB S, TZP S |
| SPBC-2012 | Klebsiella pneumoniae | Enterobacterales | Seeded Clinical | AMC I, CIP R, GEN R, SAM R |
| SPBC-2013 | Klebsiella pneumoniae | Enterobacterales | Seeded Clinical | AMC S, AMK R, CAZ S, CFZ S, CIP R, CRO S, CZA S, ETP R, FEP S, GEN R, IMP R, MEM S, MIN R, SAM S, TOB S, TZP R |
| SPBC-2014 | Klebsiella pneumoniae | Enterobacterales | Seeded Clinical | AMC S, AMK R, CAZ S, CFZ S, CIP R, CRO S, CZA S, ETP R, FEP S, GEN R, IMP R, MEM S, MIN S, SAM S, TOB S, TZP S |
| SPBC-2075 | Klebsiella pneumoniae | Enterobacterales | Seeded Clinical | AMC S, AMK R, CAZ S, CFZ S, CIP R, CRO S, CZA S, ETP R, FEP S, GEN R, IMP S, MEM S, MIN S, SAM S, TOB S, TZP S |
| SPBC-3028 | Klebsiella pneumoniae | Enterobacterales | Seeded Clinical | AMC S, AMK S, CAZ S, CFZ S, CIP S, CRO S, CZA S, ETP R, FEP S, GEN R, IMP S, MEM S, MIN S, SAM S, TOB S, TZP S |
| SPBC-1014 | Klebsiella pneumoniae | Enterobacterales | Seeded Clinical | AMC S, AMK R, CAZ R, CFZ S, CIP S, CRO S, CZA S, ETP S, FEP S, MEM S, MIN S, SAM S, TOB S, TZP R |
| SPBC-3036 | Klebsiella pneumoniae | Enterobacterales | Seeded Clinical | AMC S, AMK R, CAZ S, CFZ S, CIP R, CRO S, CZA S, ETP R, FEP S, GEN R, IMP S, MEM S, MIN S, SAM S, TOB S, TZP S |
| SPBC-2091 | Klebsiella pneumoniae | Enterobacterales | Seeded Clinical | AMC S, CIP R, GEN R, SAM R |
| SPBC-2092 | Klebsiella pneumoniae | Enterobacterales | Seeded Clinical | AMC S, AMK R, CAZ S, CFZ S, CIP R, CRO S, CZA S, ETP S, FEP S, GEN R, IMP R, MEM S, MIN S, SAM S, TOB S, TZP S |
| SPBC-2093 | Klebsiella pneumoniae | Enterobacterales | Seeded Clinical | AMC I, CIP R, GEN R, SAM R |
| SPBC-2094 | Klebsiella pneumoniae | Enterobacterales | Seeded Clinical | AMC R, AMK R, CAZ I, CFZ R, CIP S, CRO R, CZA S, ETP S, FEP S, GEN R, MEM S, MIN S, SAM R, TOB S, TZP S |
| SPBC-1015 | Klebsiella pneumoniae | Enterobacterales | Seeded Clinical | AMC S, AMK R, CAZ S, CFZ S, CIP R, CRO S, CZA S, ETP R, FEP S, GEN R, IMP R, MEM S, MIN S, SAM S, TOB S, TZP S |
| SPBC-2105 | Klebsiella pneumoniae | Enterobacterales | Seeded Clinical | AMC S, CIP R, GEN R, SAM S |
| SPBC-3041 | Klebsiella pneumoniae | Enterobacterales | Seeded Clinical | AMC R, CIP R, GEN S, SAM R |
| SPBC-3050 | Klebsiella pneumoniae | Enterobacterales | Seeded Clinical | AMC S, CIP R, GEN R |
| SPBC-2129 | Klebsiella pneumoniae | Enterobacterales | Seeded Clinical | AMC R, AMK R, CAZ R, CFZ R, CIP R, CRO R, CZA R, ETP R, FEP R, GEN R, IMP R, MEM R, MIN I, SAM R, TOB R, TZP R |
| SPBC-2130 | Klebsiella pneumoniae | Enterobacterales | Seeded Clinical | AMC R, CIP R, GEN R, SAM R |
| SPBC-3058 | Klebsiella pneumoniae | Enterobacterales | Seeded Clinical | AMC S, AMK R, CAZ S, CFZ S, CIP R, CRO S, CZA S, ETP R, FEP S, GEN R, IMP S, MEM S, MIN S, SAM S, TOB S, TZP S |
| SPBC-3059 | Klebsiella pneumoniae | Enterobacterales | Seeded Clinical | AMC S, AMK R, CAZ S, CFZ S, CIP R, CRO S, CZA S, ETP R, FEP S, GEN R, IMP S, MEM S, MIN S, SAM S, TOB S, TZP S |
| SPBC-1011 | Klebsiella pneumoniae | Enterobacterales | Seeded Clinical | AMC S, AMK R, CAZ S, CFZ S, CIP R, CRO S, CZA S, ETP R, FEP S, GEN R, IMP R, MEM S, MIN S, SAM S, TOB S, TZP S |
| SPBC-3060 | Klebsiella pneumoniae | Enterobacterales | Seeded Clinical | AMC S, AMK R, CAZ S, CFZ S, CIP R, CRO S, CZA S, ETP R, FEP S, GEN R, IMP S, MEM S, MIN S, SAM S, TOB S, TZP S |
| SPBC-2131 | Klebsiella pneumoniae | Enterobacterales | Seeded Clinical | AMC S, AMK R, CAZ S, CFZ S, CIP R, CRO S, CZA S, ETP R, FEP S, GEN R, IMP R, MEM S, MIN S, SAM S, TOB S, TZP S |
| SPBC-2132 | Klebsiella pneumoniae | Enterobacterales | Seeded Clinical | AMC S, AMK R, CAZ S, CIP R, CRO S, CZA S, ETP R, FEP S, GEN R, IMP R, MEM S, MIN S, SAM S, TOB S, TZP S |
| SPBC-3061 | Klebsiella pneumoniae | Enterobacterales | Seeded Clinical | AMC I, CIP R, GEN R, SAM R |
| SPBC-3062 | Klebsiella pneumoniae | Enterobacterales | Seeded Clinical | AMC R, AMK R, CAZ R, CFZ R, CIP R, CRO R, CZA S, ETP R, FEP R, GEN R, IMP R, MEM R, MIN I, SAM R, TOB S, TZP R |
| SPBC-3063 | Klebsiella pneumoniae | Enterobacterales | Seeded Clinical | AMC S, CIP R, GEN R, SAM R |
| SPBC-2133 | Klebsiella pneumoniae | Enterobacterales | Seeded Clinical | AMC I, CIP R, GEN R, SAM R |
| SPBC-1012 | Klebsiella pneumoniae | Enterobacterales | Seeded Clinical | AMC S, AMK R, CAZ S, CFZ S, CIP R, CRO S, CZA S, ETP R, FEP S, GEN R, IMP S, MEM S, MIN R, SAM S, TOB S, TZP S |
| SPBC-2163 | Klebsiella pneumoniae | Enterobacterales | Seeded Clinical | AMC S, CIP R, GEN R, SAM S |
| SPBC-3089 | Klebsiella pneumoniae | Enterobacterales | Seeded Clinical | AMC S, AMK R, CAZ S, CFZ S, CIP R, CRO S, CZA S, ETP R, FEP S, GEN R, IMP S, MEM S, MIN S, SAM S, TOB S, TZP S |
| SPBC-1001 | Klebsiella pneumoniae | Enterobacterales | Seeded Clinical | AMC S, CIP R, GEN R, SAM R |
| 5056 | Klebsiella pneumoniae | Enterobacterales | Challenge | AMC S, AMK S, CAZ R, CIP R, CRO R, CZA R, ETP R, FEP SDD, GEN R, IMP R, MEM S, MIN S, SAM R, TOB R, TZP S |
| 5061 | Klebsiella pneumoniae | Enterobacterales | Challenge | AMC S, AMK R, CAZ S, CIP R, CRO S, CZA S, ETP R, FEP S, GEN R, IMP R, MEM S, MIN R, SAM R, TOB S, TZP S |
| 5076 | Klebsiella pneumoniae | Enterobacterales | Challenge | AMC I, AMK R, CAZ I, CIP R, CRO S, CZA S, ETP R, FEP S, GEN R, IMP R, MEM S, MIN R, SAM R, TOB S, TZP R |
| PBC-1007 | Klebsiella pneumoniae | Enterobacterales | Fresh Clinical | AMC S, CIP I, GEN R, SAM R |
| PBC-1011 | Klebsiella pneumoniae | Enterobacterales | Fresh Clinical | AMC S, CIP R, GEN R, SAM S |
| PBC-1015 | Klebsiella pneumoniae | Enterobacterales | Fresh Clinical | AMC S, CIP R, GEN R, SAM R |
| PBC-1019 | Klebsiella pneumoniae | Enterobacterales | Fresh Clinical | AMC S, CIP R, GEN R, SAM I |
| PBC-1020 | Klebsiella pneumoniae | Enterobacterales | Fresh Clinical | AMC S, CIP R, GEN R, SAM S |
| PBC-1021 | Klebsiella pneumoniae | Enterobacterales | Fresh Clinical | AMC S, CIP R, GEN R, SAM S |
| PBC-1027 | Klebsiella pneumoniae | Enterobacterales | Fresh Clinical | AMC S, CIP R, GEN R, SAM S |
| PBC-1031 | Klebsiella pneumoniae | Enterobacterales | Fresh Clinical | AMC S, CIP R, GEN R, SAM S |
| PBC-1106 | Klebsiella pneumoniae | Enterobacterales | Fresh Clinical | AMC S, AMK R, CAZ S, CFZ S, CIP R, CRO S, CZA R, ETP R, FEP S, GEN R, IMP S, MEM S, MIN S, SAM S, TOB S, TZP S |
| PBC-1114 | Klebsiella pneumoniae | Enterobacterales | Fresh Clinical | AMC S, AMK R, CAZ S, CFZ S, CIP S, CRO S, CZA S, ETP S, FEP S, GEN R, IMP R, MEM S, MIN I, SAM S, TOB S, TZP S |
| PBC-1126 | Klebsiella pneumoniae | Enterobacterales | Fresh Clinical | AMC R, AMK S, CAZ R, CFZ R, CIP R, CRO R, CZA S, ETP S, FEP R, GEN R, IMP S, MEM S, MIN R, SAM R, TOB R, TZP R |
| PBC-1137 | Klebsiella pneumoniae | Enterobacterales | Fresh Clinical | AMC S, AMK R, CAZ S, CFZ S, CIP R, CRO S, CZA S, ETP R, FEP S, GEN R, IMP R, MEM S, MIN S, SAM S, TOB S, TZP S |
| PBC-1141 | Klebsiella pneumoniae | Enterobacterales | Fresh Clinical | AMC S, AMK R, CAZ S, CFZ S, CIP S, CRO S, CZA S, ETP S, FEP S, GEN R, IMP R, MEM S, MIN R, SAM I, TOB S, TZP R |
| PBC-1149 | Klebsiella pneumoniae | Enterobacterales | Fresh Clinical | AMC S, AMK R, CAZ S, CFZ S, CIP R, CRO S, CZA S, ETP S, FEP S, GEN R, IMP S, MEM S, MIN R, SAM S, TOB S, TZP S |
| PBC-1151 | Klebsiella pneumoniae | Enterobacterales | Fresh Clinical | AMC S, AMK R, CAZ S, CFZ S, CIP R, CRO S, CZA S, ETP R, FEP S, GEN R, MEM S, MIN S, SAM S, TOB S, TZP S |
| PBC-1152 | Klebsiella pneumoniae | Enterobacterales | Fresh Clinical | AMC S, AMK R, CAZ S, CFZ S, CIP R, CRO S, CZA R, ETP S, FEP S, GEN R, IMP S, MEM S, MIN S, SAM S, TOB S, TZP S |
| PBC-1156 | Klebsiella pneumoniae | Enterobacterales | Fresh Clinical | AMC S, AMK R, CAZ S, CFZ S, CIP R, CRO S, CZA S, ETP S, FEP S, GEN R, IMP S, MEM S, MIN S, SAM S, TOB S, TZP S |
| PBC-2101 | Klebsiella pneumoniae | Enterobacterales | Fresh Clinical | AMC S, AMK R, CAZ I, CFZ R, CIP I, CRO R, CZA R, ETP S, FEP SDD, GEN R, IMP R, MEM S, MIN S, SAM S, TOB S, TZP S |
| PBC-2103 | Klebsiella pneumoniae | Enterobacterales | Fresh Clinical | AMC S, AMK R, CAZ S, CFZ S, CIP R, CRO S, CZA S, ETP S, FEP S, GEN R, IMP R, MEM S, MIN S, SAM I, TOB S, TZP S |
| PBC-2105 | Klebsiella pneumoniae | Enterobacterales | Fresh Clinical | AMC S, AMK R, CAZ S, CFZ R, CIP R, CRO S, CZA S, ETP R, FEP S, GEN R, IMP R, MEM S, MIN R, SAM R, TOB S |
| PBC-2119 | Klebsiella pneumoniae | Enterobacterales | Fresh Clinical | AMC S, AMK R, CAZ S, CFZ S, CIP R, CRO S, CZA S, ETP R, FEP S, GEN R, IMP R, MEM S, MIN S, SAM S, TOB S, TZP S |
| PBC-2124 | Klebsiella pneumoniae | Enterobacterales | Fresh Clinical | AMC S, AMK R, CAZ S, CFZ S, CIP R, CRO S, CZA S, ETP S, FEP S, GEN S, IMP S, MEM S, MIN S, SAM S, TOB S, TZP S |
| PBC-2128 | Klebsiella pneumoniae | Enterobacterales | Fresh Clinical | AMC S, AMK R, CAZ S, CFZ S, CIP R, CRO S, CZA S, ETP R, FEP S, GEN R, IMP R, MEM S, MIN S, SAM S, TOB S, TZP S |
| PBC-2130 | Klebsiella pneumoniae | Enterobacterales | Fresh Clinical | AMC S, AMK R, CAZ S, CFZ S, CIP R, CRO S, CZA S, ETP R, FEP S, GEN R, IMP R, MEM S, MIN S, SAM S, TOB S, TZP S |
| PBC-3108 | Klebsiella pneumoniae | Enterobacterales | Fresh Clinical | AMC S, AMK S, CAZ S, CFZ S, CIP R, CRO S, CZA R, ETP R, FEP S, GEN R, IMP S, MEM S, MIN R, SAM S, TOB I, TZP S |
| PBC-3112 | Klebsiella pneumoniae | Enterobacterales | Fresh Clinical | AMC R, AMK R, CAZ R, CFZ R, CIP R, CRO R, CZA S, ETP S, FEP S, GEN R, IMP R, MEM S, MIN S, SAM R, TOB S, TZP R |
| PBC-3114 | Klebsiella pneumoniae | Enterobacterales | Fresh Clinical | AMC S, AMK R, CAZ S, CFZ S, CIP R, CRO S, CZA S, ETP R, FEP S, GEN R, IMP R, MEM S, MIN S, SAM S, TOB S, TZP S |
| PBC-3117 | Klebsiella pneumoniae | Enterobacterales | Fresh Clinical | AMC S, AMK R, CAZ S, CFZ S, CIP R, CRO S, CZA S, ETP S, FEP S, GEN R, IMP S, MEM S, MIN S, SAM S, TOB S, TZP S |
| PBC-3118 | Klebsiella pneumoniae | Enterobacterales | Fresh Clinical | AMC S, AMK R, CAZ S, CFZ S, CIP R, CRO S, CZA S, ETP R, FEP S, GEN R, IMP R, MEM S, MIN S, SAM S, TOB S, TZP S |
| PBC-3122 | Klebsiella pneumoniae | Enterobacterales | Fresh Clinical | AMC S, AMK R, CAZ S, CFZ S, CIP R, CRO S, CZA S, ETP R, FEP S, GEN R, IMP R, MEM S, MIN S, SAM S, TOB S, TZP R |
| PBC-4115 | Klebsiella pneumoniae | Enterobacterales | Fresh Clinical | AMC S, AMK R, CAZ S, CFZ S, CIP R, CRO S, CZA S, ETP S, FEP SDD, IMP I, MEM S, MIN S, SAM S, TOB S, TZP R |
| PBC-4117 | Klebsiella pneumoniae | Enterobacterales | Fresh Clinical | AMC S, AMK R, CAZ S, CFZ S, CIP R, CRO S, CZA S, ETP S, FEP S, GEN R, IMP S, MEM S, MIN S, SAM S, TOB S, TZP S |
| PBC-4119 | Klebsiella pneumoniae | Enterobacterales | Fresh Clinical | AMC S, AMK R, CAZ R, CFZ R, CIP S, CRO R, CZA S, ETP R, FEP SDD, GEN R, MEM S, MIN S, SAM I, TOB S, TZP S |
| PBC-4143 | Klebsiella pneumoniae | Enterobacterales | Fresh Clinical | AMC S, AMK R, CAZ S, CFZ S, CIP R, CRO S, CZA S, ETP R, FEP S, GEN S, IMP R, MEM S, MIN S, SAM S, TOB I, TZP S |
| PBC-4144 | Klebsiella pneumoniae | Enterobacterales | Fresh Clinical | AMC S, AMK R, CAZ S, CFZ S, CIP S, CRO S, CZA S, ETP R, FEP S, GEN R, IMP R, MEM I, MIN I, SAM I, TOB S, TZP I |
| PBC-4148 | Klebsiella pneumoniae | Enterobacterales | Fresh Clinical | AMC S, AMK R, CAZ S, CFZ S, CIP R, CRO S, CZA S, ETP S, FEP S, GEN R, IMP S, MEM S, MIN S, SAM S, TOB S, TZP S |
| PBC-4154 | Klebsiella pneumoniae | Enterobacterales | Fresh Clinical | AMC S, AMK R, CAZ R, CFZ R, CIP R, CRO R, CZA S, ETP S, FEP R, GEN R, IMP S, MEM S, MIN S, SAM R, TOB R, TZP I |
| SD-1021 | Klebsiella pneumoniae | Enterobacterales | Challenge | AMC R, AMK S, CAZ R, CFZ R, CIP R, CRO R, CZA S, ETP S, FEP R, GEN R, IMP R, MEM S, MIN I, SAM R, TOB R, TZP R |
| SD-1022 | Klebsiella pneumoniae | Enterobacterales | Challenge | AMC S, AMK R, CAZ S, CFZ S, CIP S, CRO S, CZA S, ETP R, FEP S, GEN R, IMP R, MEM S, MIN R, SAM I, TOB S, TZP S |
| SD-1033 | Klebsiella pneumoniae | Enterobacterales | Challenge | AMC R, AMK R, CAZ R, CFZ R, CIP R, CRO R, CZA R, ETP R, FEP R, GEN R, IMP R, MEM R, MIN R, SAM R, TOB R, TZP R |
| SD-1037 | Klebsiella pneumoniae | Enterobacterales | Challenge | AMC R, AMK R, CAZ R, CFZ R, CIP R, CRO R, CZA S, ETP R, FEP R, GEN R, IMP R, MIN I, SAM R, TOB R, TZP R |
| SD-1060 | Klebsiella pneumoniae | Enterobacterales | Challenge | AMC R, AMK S, CAZ R, CFZ R, CIP R, CRO R, CZA S, ETP R, FEP R, GEN S, IMP R, MEM R, MIN R, SAM R, TOB R, TZP R |
| SD-1063 | Klebsiella pneumoniae | Enterobacterales | Challenge | AMC R, AMK R, CAZ R, CFZ R, CIP R, CRO R, CZA R, ETP R, FEP SDD, GEN R, IMP R, MIN S, SAM R, TOB R, TZP R |
| SD-1901 | Klebsiella pneumoniae | Enterobacterales | Challenge | AMC R, AMK I, CAZ R, CFZ R, CIP R, CRO R, CZA S, ETP R, FEP R, GEN R, IMP R, MEM R, MIN S, SAM R, TOB R, TZP R |
| SD-1906 | Klebsiella pneumoniae | Enterobacterales | Challenge | AMC R, AMK S, CAZ R, CFZ R, CIP R, CRO R, CZA S, ETP R, FEP R, GEN R, IMP R, MEM R, MIN I, SAM R, TOB R, TZP R |
| SD-1965 | Klebsiella pneumoniae | Enterobacterales | Challenge | AMC I, AMK R, CAZ R, CFZ R, CIP R, CRO R, CZA S, ETP R, FEP R, GEN R, IMP R, MIN I, SAM R, TOB R, TZP R |
| SD-1979 | Klebsiella pneumoniae | Enterobacterales | Challenge | AMC R, AMK S, CAZ R, CFZ R, CIP R, CRO R, CZA S, ETP R, FEP R, GEN R, IMP R, MEM R, MIN R, SAM R, TOB R, TZP R |
| SD-1980 | Klebsiella pneumoniae | Enterobacterales | Challenge | AMC R, CAZ R, CFZ R, CIP R, CRO R, CZA R, ETP R, FEP R, IMP R, MEM R, MIN R, SAM R, TOB R, TZP R |
| SD-1981 | Klebsiella pneumoniae | Enterobacterales | Challenge | AMC R, AMK R, CAZ R, CFZ R, CIP R, CRO R, CZA S, ETP R, FEP R, GEN R, IMP R, MEM R, MIN I, SAM R, TOB R, TZP R |
| SD-1983 | Klebsiella pneumoniae | Enterobacterales | Challenge | AMC R, AMK R, CAZ R, CFZ R, CIP R, CRO R, CZA R, ETP R, FEP R, GEN R, IMP R, MEM R, MIN I, SAM R, TOB R, TZP R |
| SD-2028 | Klebsiella pneumoniae | Enterobacterales | Challenge | AMC R, AMK S, CAZ R, CFZ R, CIP R, CRO R, CZA S, ETP R, FEP R, GEN S, IMP R, MEM R, MIN I, SAM R, TOB R, TZP R |
| SD-2584 | Klebsiella pneumoniae | Enterobacterales | Challenge | AMC R, AMK R, CAZ R, CFZ R, CIP R, CRO R, CZA S, FEP R, GEN R, IMP R, MEM S, MIN R, SAM R, TOB R, TZP R |
| SD-2586 | Klebsiella pneumoniae | Enterobacterales | Challenge | AMC R, AMK R, CAZ R, CFZ R, CIP R, CRO R, CZA S, ETP R, FEP R, GEN R, MEM R, MIN R, SAM R, TOB R, TZP R |
| SD-2596 | Klebsiella pneumoniae | Enterobacterales | Challenge | AMC R, AMK S, CAZ R, CFZ R, CIP R, CRO R, CZA S, ETP R, FEP R, GEN R, IMP R, MEM R, MIN S, SAM R, TOB R, TZP R |
| SD-5405 | Klebsiella pneumoniae | Enterobacterales | Challenge | AMC I, AMK S, CAZ R, CFZ R, CIP R, CRO R, CZA S, ETP S, GEN R, IMP R, MEM S, MIN S, SAM R, TOB R |
| SD-5407 | Klebsiella pneumoniae | Enterobacterales | Challenge | AMC S, AMK R, CAZ R, CFZ R, CIP I, CRO R, CZA S, ETP S, FEP R, GEN R, IMP R, MEM S, MIN S, SAM I, TOB S, TZP S |
| SD-5409 | Klebsiella pneumoniae | Enterobacterales | Challenge | AMC I, AMK S, CAZ R, CFZ R, CIP R, CRO R, CZA S, ETP S, FEP R, GEN R, IMP R, MEM S, MIN R, SAM R, TOB I, TZP R |
| SD-5414 | Klebsiella pneumoniae | Enterobacterales | Challenge | AMC S, AMK R, CAZ I, CFZ R, CRO R, CZA R, ETP S, FEP R, GEN R, IMP R, MEM S, MIN S, SAM R, TOB S, TZP S |
| SD-5418 | Klebsiella pneumoniae | Enterobacterales | Challenge | AMC S, AMK R, CAZ R, CFZ R, CIP I, CRO R, CZA S, ETP R, GEN R, IMP R, MEM S, MIN S, SAM R, TOB S, TZP S |
| SD-5420 | Klebsiella pneumoniae | Enterobacterales | Challenge | AMC S, AMK R, CAZ R, CFZ R, CIP R, CRO R, CZA S, ETP S, GEN R, IMP R, MEM S, MIN I, SAM R, TOB S, TZP S |
| SD-5422 | Klebsiella pneumoniae | Enterobacterales | Challenge | AMC S, AMK R, CAZ S, CFZ R, CIP I, CRO R, CZA R, ETP S, FEP SDD, GEN R, IMP R, MEM S, MIN S, SAM R, TOB S, TZP S |
| SD-5424 | Klebsiella pneumoniae | Enterobacterales | Challenge | AMC S, AMK S, CAZ R, CFZ R, CIP R, CRO R, CZA R, ETP S, GEN R, IMP R, MEM S, MIN S, SAM R, TOB R, TZP S |
| SPBC-2038 | Proteus mirabilis | Enterobacterales | Seeded Clinical | AMC R, AMP R, CIP S, CRO S, CZA S, ETP S, FEP R, GEN R, MEM S, SAM S, TZP R |
| SPBC-3017 | Proteus mirabilis | Enterobacterales | Seeded Clinical | AMC R, AMP R, CIP S, CRO S, CZA S, ETP S, FEP R, GEN S, MEM S, SAM S, TZP R |
| SPBC-2039 | Proteus mirabilis | Enterobacterales | Seeded Clinical | AMC R, AMP R, CIP S, CRO S, CZA S, ETP S, FEP S, GEN R, MEM S, SAM S, TZP R |
| SPBC-2040 | Proteus mirabilis | Enterobacterales | Seeded Clinical | AMC R, AMP R, CIP S, CRO S, CZA S, ETP S, FEP R, GEN R, MEM S, SAM S, TZP R |
| SPBC-2041 | Proteus mirabilis | Enterobacterales | Seeded Clinical | AMC R, AMP R, CIP S, CRO S, CZA S, ETP S, FEP R, GEN R, MEM R, SAM S, TZP R |
| SPBC-2042 | Proteus mirabilis | Enterobacterales | Seeded Clinical | AMC R, CIP S, CRO S, CZA S, ETP S, FEP R, GEN R, MEM R, SAM S, TZP R |
| SPBC-2069 | Proteus mirabilis | Enterobacterales | Seeded Clinical | AMC R, AMP R, CIP R, CRO S, CZA S, ETP S, FEP R, GEN R, MEM S, SAM S, TZP R |
| SPBC-2070 | Proteus mirabilis | Enterobacterales | Seeded Clinical | AMC R, AMP R, CIP S, CRO S, CZA S, ETP S, FEP S, GEN R, MEM R, SAM S, TZP R |
| SPBC-3026 | Proteus mirabilis | Enterobacterales | Seeded Clinical | AMC R, AMP R, CIP S, CRO S, CZA S, ETP S, FEP R, GEN S, MEM S, SAM S, TZP R |
| SPBC-2071 | Proteus mirabilis | Enterobacterales | Seeded Clinical | AMC R, AMP R, CIP S, CRO S, CZA S, ETP S, FEP R, GEN R, MEM S, SAM S, TZP R |
| SPBC-2110 | Proteus mirabilis | Enterobacterales | Seeded Clinical | AMC S, AMP R, CIP R, CRO S, CZA S, ETP S, FEP R, GEN R, MEM S, SAM I, TZP R |
| SPBC-2111 | Proteus mirabilis | Enterobacterales | Seeded Clinical | AMC R, AMP R, CIP S, CRO S, CZA S, ETP S, FEP R, GEN R, MEM S, SAM S, TZP R |
| SPBC-3079 | Proteus mirabilis | Enterobacterales | Seeded Clinical | AMC R, AMP R, CIP S, CRO S, CZA S, ETP S, FEP R, GEN R, MEM S, SAM S, TZP R |
| SPBC-2149 | Proteus mirabilis | Enterobacterales | Seeded Clinical | AMC R, AMP R, CIP S, CRO S, CZA S, ETP S, FEP R, GEN S, MEM S, SAM S, TZP R |
| SPBC-1031 | Proteus mirabilis | Enterobacterales | Seeded Clinical | AMC R, AMP R, CIP S, CRO S, CZA S, FEP R, GEN S, MEM S, SAM S, TZP R |
| PBC-1154 | Proteus mirabilis | Enterobacterales | Fresh Clinical | AMC R, AMP R, CIP S, CRO S, CZA S, ETP R, FEP S, GEN S, MEM S, SAM S, TZP R |
| PBC-1173 | Proteus mirabilis | Enterobacterales | Fresh Clinical | AMC R, AMP R, CIP S, CRO S, CZA S, ETP R, FEP S, GEN R, MEM S, SAM S, TZP R |
| PBC-2127 | Proteus mirabilis | Enterobacterales | Fresh Clinical | AMC R, AMP S, CIP S, CRO S, CZA S, ETP S, FEP R, GEN S, MEM R, SAM S, TZP R |
| PBC-2129 | Proteus mirabilis | Enterobacterales | Fresh Clinical | AMC R, AMP R, CIP S, CRO S, CZA S, ETP S, FEP R, GEN R, MEM S, SAM S, TZP R |
| PBC-3101 | Proteus mirabilis | Enterobacterales | Fresh Clinical | AMC R, AMP R, CIP S, CRO S, CZA S, ETP S, FEP R, GEN R, MEM S, SAM S, TZP R |
| SPBC-3018 | Proteus vulgaris | Enterobacterales | Seeded Clinical | AMC S, CIP S, CZA S, ETP S, FEP R, GEN S, MEM S, TZP R |
| SPBC-2043 | Proteus vulgaris | Enterobacterales | Seeded Clinical | AMC S, CIP S, CZA S, ETP S, FEP R, GEN R, MEM S, TZP R |
| SPBC-2044 | Proteus vulgaris | Enterobacterales | Seeded Clinical | AMC S, CIP S, CZA S, ETP S, FEP R, GEN R, MEM R, TZP R |
| SPBC-2045 | Proteus vulgaris | Enterobacterales | Seeded Clinical | AMC S, CIP S, CZA S, ETP S, FEP R, GEN R, MEM R, TZP R |
| SPBC-2046 | Proteus vulgaris | Enterobacterales | Seeded Clinical | AMC S, CIP S, CZA S, ETP S, FEP R, GEN R, MEM S, TZP R |
| SPBC-2047 | Proteus vulgaris | Enterobacterales | Seeded Clinical | AMC S, CIP S, CZA S, ETP S, FEP R, GEN R, MEM R, TZP R |
| SPBC-2048 | Proteus vulgaris | Enterobacterales | Seeded Clinical | AMC S, CIP S, CZA S, ETP S, FEP S, GEN R, MEM R, TZP R |
| SPBC-2049 | Proteus vulgaris | Enterobacterales | Seeded Clinical | AMC S, CIP S, CZA S, ETP S, FEP S, GEN R, MEM R, TZP R |
| SPBC-2050 | Proteus vulgaris | Enterobacterales | Seeded Clinical | AMC S, CIP S, CZA S, ETP S, FEP R, GEN R, MEM R, TZP R |
| SPBC-2081 | Proteus vulgaris | Enterobacterales | Seeded Clinical | AMC R, CIP S, CZA S, ETP S, FEP S, GEN R, MEM R, TZP R |
| SPBC-2098 | Proteus vulgaris | Enterobacterales | Seeded Clinical | AMC S, CIP S, CZA S, ETP S, FEP R, GEN R, MEM R, TZP R |
| SPBC-3049 | Proteus vulgaris | Enterobacterales | Seeded Clinical | AMC S, CIP S, CZA S, ETP S, FEP R, GEN S, MEM S, TZP R |
| SPBC-2113 | Proteus vulgaris | Enterobacterales | Seeded Clinical | AMC S, CIP S, CZA S, ETP S, FEP S, GEN R, MEM R, TZP R |
| SPBC-1033 | Proteus vulgaris | Enterobacterales | Seeded Clinical | AMC S, CIP S, CZA S, ETP S, FEP S, GEN S, MEM S, TZP R |
| SPBC-2150 | Proteus vulgaris | Enterobacterales | Seeded Clinical | AMC S, CIP S, CZA S, ETP S, FEP R, GEN R, MEM R, TZP R |
| SPBC-1034 | Proteus vulgaris | Enterobacterales | Seeded Clinical | AMC S, CIP R, CZA S, ETP S, FEP R, GEN S, MEM S, TZP R |
| SPBC-2151 | Proteus vulgaris | Enterobacterales | Seeded Clinical | AMC R, CIP S, CZA S, ETP S, FEP R, GEN R, MEM S, TZP R |
| SPBC-3080 | Proteus vulgaris | Enterobacterales | Seeded Clinical | AMC S, CIP S, CZA S, ETP S, FEP R, GEN S, MEM S, TZP R |
| SPBC-2152 | Proteus vulgaris | Enterobacterales | Seeded Clinical | AMC R, CIP S, CZA S, ETP S, FEP R, GEN R, MEM R, TZP R |
| SPBC-2153 | Proteus vulgaris | Enterobacterales | Seeded Clinical | AMC S, CIP R, CZA S, ETP S, FEP R, GEN R, MEM R, TZP R |
| SPBC-1032 | Proteus vulgaris | Enterobacterales | Seeded Clinical | AMC S, CIP S, CZA S, ETP S, FEP R, GEN R, MEM S, TZP R |
| SPBC-3022 | Acinetobacter baumannii complex | Acinetobacter baumannii | Seeded Clinical | AMK S, MIN R, SAM S |
| SPBC-3023 | Acinetobacter baumannii complex | Acinetobacter baumannii | Seeded Clinical | AMK S, IMP R, MEM S, MIN R, SAM R, TZP R |
| SPBC-1016 | Acinetobacter baumannii complex | Acinetobacter baumannii | Seeded Clinical | AMK S, IMP R, MEM S, MIN S, SAM S, TZP S |
| SPBC-2057 | Acinetobacter baumannii complex | Acinetobacter baumannii | Seeded Clinical | AMK R, IMP R, MEM R, MIN R, SAM R, TZP R |
| SPBC-3024 | Acinetobacter baumannii complex | Acinetobacter baumannii | Seeded Clinical | AMK R, IMP R, MEM R, MIN R, SAM R, TZP R |
| SPBC-2058 | Acinetobacter baumannii complex | Acinetobacter baumannii | Seeded Clinical | AMK S, IMP R, MEM S, MIN S, SAM S, TZP R |
| SPBC-2059 | Acinetobacter baumannii complex | Acinetobacter baumannii | Seeded Clinical | AMK I, IMP R, MEM S, MIN R, SAM S, TZP I |
| SPBC-2060 | Acinetobacter baumannii complex | Acinetobacter baumannii | Seeded Clinical | AMK S, IMP R, MEM R, MIN R, SAM R, TZP R |
| SPBC-1018 | Acinetobacter baumannii complex | Acinetobacter baumannii | Seeded Clinical | AMK S, IMP R, MEM S, MIN S, SAM S, TZP R |
| SPBC-2061 | Acinetobacter baumannii complex | Acinetobacter baumannii | Seeded Clinical | AMK S, IMP R, MEM S, MIN R, SAM S, TZP R |
| SPBC-1019 | Acinetobacter baumannii complex | Acinetobacter baumannii | Seeded Clinical | AMK S, IMP R, MEM S, MIN S, SAM S, TZP R |
| SPBC-3031 | Acinetobacter baumannii complex | Acinetobacter baumannii | Seeded Clinical | AMK S, IMP R, MEM R, MIN S, SAM R, TZP R |
| SPBC-2083 | Acinetobacter baumannii complex | Acinetobacter baumannii | Seeded Clinical | AMK R, IMP R, MEM R, MIN R, SAM R, TZP R |
| SPBC-3052 | Acinetobacter baumannii complex | Acinetobacter baumannii | Seeded Clinical | AMK R, IMP R, MEM R, MIN S, SAM R, TZP R |
| SPBC-3053 | Acinetobacter baumannii complex | Acinetobacter baumannii | Seeded Clinical | AMK R, IMP R, MEM R, SAM R, TZP R |
| SPBC-2122 | Acinetobacter baumannii complex | Acinetobacter baumannii | Seeded Clinical | AMK R, IMP R, MEM R, MIN S, SAM R, TZP R |
| SPBC-3054 | Acinetobacter baumannii complex | Acinetobacter baumannii | Seeded Clinical | AMK S, IMP R, MEM R, MIN S, SAM R, TZP R |
| SPBC-2123 | Acinetobacter baumannii complex | Acinetobacter baumannii | Seeded Clinical | AMK R, IMP R, MEM R, MIN R, SAM R, TZP R |
| SPBC-2160 | Acinetobacter baumannii complex | Acinetobacter baumannii | Seeded Clinical | AMK S, IMP R, MEM S, MIN R, SAM S, TZP S |
| SPBC-3084 | Acinetobacter baumannii complex | Acinetobacter baumannii | Seeded Clinical | AMK S, IMP S, MEM R, MIN S, SAM S, TZP R |
| SPBC-3085 | Acinetobacter baumannii complex | Acinetobacter baumannii | Seeded Clinical | AMK R, IMP R, MEM R, MIN S, SAM I, TZP R |
| SPBC-2161 | Acinetobacter baumannii complex | Acinetobacter baumannii | Seeded Clinical | AMK S, IMP R, MEM S, MIN R, SAM R, TZP R |
| SPBC-3086 | Acinetobacter baumannii complex | Acinetobacter baumannii | Seeded Clinical | AMK R, IMP R, MEM R, MIN R, SAM R, TZP R |
| SPBC-3087 | Acinetobacter baumannii complex | Acinetobacter baumannii | Seeded Clinical | AMK S, IMP R, MEM S, MIN R, SAM S, TZP R |
| SPBC-2162 | Acinetobacter baumannii complex | Acinetobacter baumannii | Seeded Clinical | AMK S, IMP R, MEM R, MIN R, SAM R, TZP R |
| SPBC-3088 | Acinetobacter baumannii complex | Acinetobacter baumannii | Seeded Clinical | AMK S, IMP R, MEM S, MIN S, SAM R, TZP R |
| SPBC-1017 | Acinetobacter baumannii complex | Acinetobacter baumannii | Seeded Clinical | AMK S, IMP R, MEM S, MIN S, SAM S, TZP R |
| SD-1001 | Acinetobacter baumannii complex | Acinetobacter baumannii | Challenge | AMK R, IMP R, MEM R, MIN R, SAM R, TZP R |
| SD-1002 | Acinetobacter baumannii complex | Acinetobacter baumannii | Challenge | AMK R, IMP R, MEM R, MIN S, SAM R, TZP R |
| SD-1004 | Acinetobacter baumannii complex | Acinetobacter baumannii | Challenge | AMK S, IMP R, MEM R, MIN S, SAM R, TZP R |
| SD-4393 | Acinetobacter baumannii complex | Acinetobacter baumannii | Challenge | AMK R, IMP R, MEM R, MIN R, SAM R, TZP R |
| SD-4394 | Acinetobacter baumannii complex | Acinetobacter baumannii | Challenge | AMK R, IMP R, MEM R, MIN R, SAM R, TZP R |
| SD-954 | Acinetobacter baumannii complex | Acinetobacter baumannii | Challenge | AMK R, IMP R, MEM R, MIN R, SAM R, TZP R |
| SD-967 | Acinetobacter baumannii complex | Acinetobacter baumannii | Challenge | AMK R, IMP R, MEM R, MIN R, SAM R, TZP R |
| SD-986 | Acinetobacter baumannii complex | Acinetobacter baumannii | Challenge | AMK S, IMP R, MEM R, MIN R, SAM R, TZP R |
| SD-988 | Acinetobacter baumannii complex | Acinetobacter baumannii | Challenge | AMK R, IMP R, MEM R, MIN R, SAM R, TZP R |
| SD-990 | Acinetobacter baumannii complex | Acinetobacter baumannii | Challenge | AMK R, IMP R, MEM R, MIN S, SAM R, TZP R |
| PBC-4150 | Acinetobacter baumannii complex | Acinetobacter baumannii | Fresh Clinical | AMK R, IMP R, MEM R, MIN R, SAM R, TZP R |
| SD-5416 | Klebsiella pneumoniae | Enterobacterales | Challenge | AMK S, CAZ R, CFZ R, CIP R, CRO R, CZA S, ETP S, FEP R, GEN R, IMP R, MEM S, MIN S, SAM R, TOB R, TZP S |
| SPBC-3021 | Pseudomonas aeruginosa | Pseudomonas aeruginosa | Seeded Clinical | AMK S, CAZ S, CIP S, CZA S, FEP S, GEN S, MEM S, TOB S, TZP S |
| SPBC-2056 | Pseudomonas aeruginosa | Pseudomonas aeruginosa | Seeded Clinical | AMK S, CAZ S, CIP S, CZA S, FEP S, GEN S, MEM S, TOB S, TZP S |
| SPBC-3029 | Pseudomonas aeruginosa | Pseudomonas aeruginosa | Seeded Clinical | AMK S, CAZ S, CIP S, CZA S, FEP S, GEN S, MEM S, TOB S, TZP S |
| SPBC-2076 | Pseudomonas aeruginosa | Pseudomonas aeruginosa | Seeded Clinical | AMK S, CAZ S, CIP S, CZA S, FEP S, GEN S, MEM S, TOB S, TZP S |
| SPBC-3045 | Pseudomonas aeruginosa | Pseudomonas aeruginosa | Seeded Clinical | AMK S, CAZ R, CIP R, CZA R, FEP R, GEN S, MEM R, TOB I, TZP S |
| SPBC-3051 | Pseudomonas aeruginosa | Pseudomonas aeruginosa | Seeded Clinical | AMK S, CAZ S, CIP S, CZA S, FEP S, GEN S, MEM S, TOB S, TZP S |
| SPBC-3081 | Pseudomonas aeruginosa | Pseudomonas aeruginosa | Seeded Clinical | AMK S, CIP S, CZA S, FEP R, GEN S, MEM S, TOB S, TZP R |
| SPBC-2157 | Pseudomonas aeruginosa | Pseudomonas aeruginosa | Seeded Clinical | AMK S, CAZ S, CIP S, CZA S, FEP S, GEN S, MEM S, TOB S, TZP S |
| SPBC-2158 | Pseudomonas aeruginosa | Pseudomonas aeruginosa | Seeded Clinical | AMK S, CAZ S, CIP I, CZA S, FEP R, GEN S, MEM S, TOB S, TZP S |
| SPBC-3082 | Pseudomonas aeruginosa | Pseudomonas aeruginosa | Seeded Clinical | AMK S, CAZ S, CIP S, CZA S, FEP S, GEN S, MEM I, TOB R, TZP S |
| SPBC-1036 | Pseudomonas aeruginosa | Pseudomonas aeruginosa | Seeded Clinical | AMK S, CAZ S, CIP S, CZA S, FEP S, GEN S, MEM S, TOB S, TZP S |
| SPBC-2159 | Pseudomonas aeruginosa | Pseudomonas aeruginosa | Seeded Clinical | AMK S, CAZ S, CIP S, CZA S, FEP S, GEN S, MEM S, TOB S, TZP S |
| SPBC-1035 | Pseudomonas aeruginosa | Pseudomonas aeruginosa | Seeded Clinical | AMK S, CAZ S, CIP S, CZA S, FEP S, GEN S, MEM S, TOB S, TZP S |
| SPBC-3083 | Pseudomonas aeruginosa | Pseudomonas aeruginosa | Seeded Clinical | AMK S, CAZ S, CIP S, CZA S, FEP S, MEM I, TOB S, TZP S |
| SPBC-1037 | Pseudomonas aeruginosa | Pseudomonas aeruginosa | Seeded Clinical | AMK S, CAZ S, CIP S, CZA S, FEP S, GEN S, MEM S, TOB S, TZP S |
| PBC-1116 | Pseudomonas aeruginosa | Pseudomonas aeruginosa | Fresh Clinical | AMK S, CAZ S, CIP S, CZA S, FEP S, GEN S, MEM S, TOB S, TZP S |
| PBC-1119 | Pseudomonas aeruginosa | Pseudomonas aeruginosa | Fresh Clinical | AMK S, CAZ S, CIP S, CZA S, FEP S, GEN S, MEM S, TOB S, TZP S |
| PBC-1130 | Pseudomonas aeruginosa | Pseudomonas aeruginosa | Fresh Clinical | AMK S, CAZ S, CIP S, CZA S, FEP S, GEN S, MEM S, TOB S, TZP S |
| PBC-1134 | Pseudomonas aeruginosa | Pseudomonas aeruginosa | Fresh Clinical | AMK S, GEN S |
| PBC-1142 | Pseudomonas aeruginosa | Pseudomonas aeruginosa | Fresh Clinical | AMK S, CAZ S, CIP S, CZA S, FEP S, GEN S, MEM S, TOB S, TZP S |
| PBC-1155 | Pseudomonas aeruginosa | Pseudomonas aeruginosa | Fresh Clinical | AMK S, CAZ S, CIP S, CZA S, FEP S, GEN S, MEM S, TOB S, TZP S |
| PBC-1162 | Pseudomonas aeruginosa | Pseudomonas aeruginosa | Fresh Clinical | AMK S, CIP I, CZA S, FEP R, GEN S, MEM R, TOB S, TZP I |
| PBC-1169 | Pseudomonas aeruginosa | Pseudomonas aeruginosa | Fresh Clinical | AMK S, CAZ S, CIP S, CZA S, FEP S, GEN S, MEM S, TOB S, TZP S |
| PBC-2102 | Pseudomonas aeruginosa | Pseudomonas aeruginosa | Fresh Clinical | AMK S, CAZ S, CIP S, CZA S, FEP S, GEN S, MEM S, TOB S, TZP S |
| PBC-2104 | Pseudomonas aeruginosa | Pseudomonas aeruginosa | Fresh Clinical | AMK S, CIP S, CZA S, FEP S, GEN S, MEM S, TOB S, TZP S |
| PBC-2109 | Pseudomonas aeruginosa | Pseudomonas aeruginosa | Fresh Clinical | AMK S, CAZ S, CIP S, CZA S, FEP S, GEN R, MEM S, TOB S, TZP S |
| PBC-3113 | Pseudomonas aeruginosa | Pseudomonas aeruginosa | Fresh Clinical | AMK S, CAZ S, CIP S, CZA S, FEP S, GEN S, MEM S, TOB S, TZP S |
| PBC-4136 | Pseudomonas aeruginosa | Pseudomonas aeruginosa | Fresh Clinical | AMK I, CAZ S, CIP S, CZA S, FEP S, GEN S, MEM S, TOB R, TZP S |
| SD-1427 | Pseudomonas aeruginosa | Pseudomonas aeruginosa | Challenge | AMK R, CAZ R, CIP R, CZA R, FEP R, GEN R, MEM R, TOB R, TZP R |
| SD-1441 | Pseudomonas aeruginosa | Pseudomonas aeruginosa | Challenge | AMK S, CAZ S, CIP R, CZA S, FEP S, GEN R, MEM I, TOB R, TZP S |
| SD-1442 | Pseudomonas aeruginosa | Pseudomonas aeruginosa | Challenge | AMK S, CAZ S, CIP S, CZA S, FEP S, GEN S, MEM R, TOB S, TZP S |
| SD-1444 | Pseudomonas aeruginosa | Pseudomonas aeruginosa | Challenge | AMK I, CAZ R, CIP R, CZA R, FEP R, GEN R, MEM R, TOB R, TZP R |
| SD-1446 | Pseudomonas aeruginosa | Pseudomonas aeruginosa | Challenge | AMK S, CAZ S, CIP S, CZA S, FEP S, GEN S, MEM S, TOB S, TZP S |
| SD-1447 | Pseudomonas aeruginosa | Pseudomonas aeruginosa | Challenge | AMK S, CAZ S, CIP S, CZA S, FEP S, GEN S, MEM S, TOB S, TZP S |
| SD-1454 | Pseudomonas aeruginosa | Pseudomonas aeruginosa | Challenge | AMK R, CAZ R, CIP R, CZA R, FEP R, GEN R, MEM R, TOB R, TZP R |
| SD-1456 | Pseudomonas aeruginosa | Pseudomonas aeruginosa | Challenge | AMK I, CAZ R, CIP R, CZA R, FEP R, GEN R, MEM I, TOB R, TZP R |
| SD-1463 | Pseudomonas aeruginosa | Pseudomonas aeruginosa | Challenge | AMK I, CAZ R, CIP R, CZA R, FEP R, GEN R, MEM R, TOB R, TZP R |
| SD-1476 | Pseudomonas aeruginosa | Pseudomonas aeruginosa | Challenge | AMK S, CAZ R, CIP R, CZA R, FEP R, GEN S, MEM R, TOB I, TZP I |
| SD-1478 | Pseudomonas aeruginosa | Pseudomonas aeruginosa | Challenge | AMK S, CAZ R, CIP R, CZA S, FEP R, GEN S, MEM R, TOB S, TZP R |
| SD-1481 | Pseudomonas aeruginosa | Pseudomonas aeruginosa | Challenge | AMK S, CAZ S, CIP R, CZA S, FEP S, GEN S, MEM I, TOB S, TZP S |
| SD-1482 | Pseudomonas aeruginosa | Pseudomonas aeruginosa | Challenge | AMK S, CAZ S, CIP S, CZA S, FEP S, GEN S, MEM S, TOB S, TZP S |
| SD-1483 | Pseudomonas aeruginosa | Pseudomonas aeruginosa | Challenge | AMK S, CAZ S, CIP S, CZA S, FEP S, GEN S, MEM S, TOB S, TZP S |
| SD-1489 | Pseudomonas aeruginosa | Pseudomonas aeruginosa | Challenge | AMK S, CAZ S, CIP R, CZA S, FEP S, GEN R, MEM I, TOB R, TZP S |
| SD-4335 | Pseudomonas aeruginosa | Pseudomonas aeruginosa | Challenge | AMK R, CAZ R, CIP R, CZA R, FEP R, GEN R, MEM R, TOB R, TZP R |
| SPBC-3047 | Proteus mirabilis | Enterobacterales | Seeded Clinical | AMP R, CIP S, CRO S, CZA S, ETP S, FEP R, GEN S, MEM R, SAM R, TZP R |
| SPBC-3046 | Proteus mirabilis | Enterobacterales | Seeded Clinical | AMP R, CIP R, CRO S, CZA S, ETP S, FEP S, GEN R, MEM S, SAM R, TZP R |
| SD-1038 | Klebsiella pneumoniae | Enterobacterales | Challenge | CFZ R, CIP R, CRO R, ETP R, IMP R, MEM R, MIN R, SAM R, TOB R |
| SPBC-2032 | Citrobacter freundii complex | Enterobacterales | Seeded Clinical | CIP S, CRO S, CZA S, ETP S, FEP R, GEN R, MEM S |
| SPBC-1022 | Citrobacter freundii complex | Enterobacterales | Seeded Clinical | CIP S, CRO S, CZA S, ETP S, FEP R, GEN R, MEM S |
| SPBC-3015 | Citrobacter freundii complex | Enterobacterales | Seeded Clinical | CIP S, CRO R, CZA S, ETP I, FEP R, GEN R, MEM S |
| SPBC-2033 | Citrobacter freundii complex | Enterobacterales | Seeded Clinical | CIP S, CRO S, CZA S, ETP R, FEP R, GEN R, MEM S |
| SPBC-2034 | Citrobacter freundii complex | Enterobacterales | Seeded Clinical | CIP S, CRO S, CZA S, ETP S, FEP R, GEN R, MEM S |
| SPBC-2077 | Citrobacter freundii complex | Enterobacterales | Seeded Clinical | CIP R, CRO R, CZA S, ETP S, FEP S, GEN S, MEM S |
| SPBC-2088 | Citrobacter freundii complex | Enterobacterales | Seeded Clinical | CIP S, CRO S, CZA S, ETP S, FEP R, GEN R, MEM S |
| SPBC-3042 | Citrobacter freundii complex | Enterobacterales | Seeded Clinical | CIP S, CRO R, CZA S, ETP S, FEP S, GEN R, MEM S |
| SPBC-2117 | Citrobacter freundii complex | Enterobacterales | Seeded Clinical | CIP S, CRO S, CZA S, ETP S, FEP S, GEN R, MEM S |
| SPBC-3076 | Citrobacter freundii complex | Enterobacterales | Seeded Clinical | CIP S, CRO S, CZA S, ETP S, FEP R, GEN R, MEM S |
| SPBC-3077 | Citrobacter freundii complex | Enterobacterales | Seeded Clinical | CIP R, CRO S, CZA S, ETP S, FEP R, GEN R, MEM S |
| SPBC-2143 | Citrobacter freundii complex | Enterobacterales | Seeded Clinical | CIP S, CRO S, CZA S, ETP S, FEP R, GEN R, MEM S |
| SPBC-2144 | Citrobacter freundii complex | Enterobacterales | Seeded Clinical | CIP S, CRO S, CZA S, ETP S, FEP R, GEN R, MEM S |
| SPBC-1020 | Citrobacter freundii complex | Enterobacterales | Seeded Clinical | CIP S, CRO R, CZA S, ETP S, FEP S, GEN R, MEM S |
| SPBC-3090 | Citrobacter freundii complex | Enterobacterales | Seeded Clinical | CIP R, CRO S, CZA S, ETP S, FEP R, GEN R, MEM S |
| SPBC-3091 | Citrobacter freundii complex | Enterobacterales | Seeded Clinical | CIP S, CRO S, CZA S, ETP S, FEP R, GEN R, MEM S |
| SPBC-1021 | Citrobacter freundii complex | Enterobacterales | Seeded Clinical | CIP S, CRO S, CZA S, ETP S, FEP R, GEN R, MEM S |
| SPBC-3093 | Citrobacter freundii complex | Enterobacterales | Seeded Clinical | CIP S, CRO S, CZA S, ETP S, FEP R, GEN R, MEM S |
| SPBC-2164 | Citrobacter freundii complex | Enterobacterales | Seeded Clinical | CIP S, CRO R, CZA S, ETP S, FEP R, GEN R, MEM S |
| SPBC-2165 | Citrobacter freundii complex | Enterobacterales | Seeded Clinical | CIP S, CRO S, CZA S, ETP S, FEP R, GEN R, MEM S |
| SPBC-3016 | Citrobacter koseri | Enterobacterales | Seeded Clinical | CIP S, CRO S, CZA S, ETP S, FEP R, GEN R, MEM S, SAM S, TZP S |
| SPBC-2035 | Citrobacter koseri | Enterobacterales | Seeded Clinical | CIP S, CRO S, CZA S, ETP S, FEP S, GEN R, MEM S, SAM S, TZP S |
| SPBC-2036 | Citrobacter koseri | Enterobacterales | Seeded Clinical | CIP S, CRO S, CZA S, ETP S, FEP R, GEN R, MEM S, SAM S, TZP R |
| SPBC-2037 | Citrobacter koseri | Enterobacterales | Seeded Clinical | CIP S, CRO S, CZA S, ETP S, FEP R, GEN R, MEM S, SAM S, TZP S |
| SPBC-2078 | Citrobacter koseri | Enterobacterales | Seeded Clinical | CIP S, CRO S, CZA S, ETP R, FEP R, GEN R, MEM S, SAM R, TZP R |
| SPBC-1023 | Citrobacter koseri | Enterobacterales | Seeded Clinical | CIP S, CRO S, CZA S, ETP S, FEP R, GEN R, MEM S, SAM S, TZP S |
| SPBC-2146 | Citrobacter koseri | Enterobacterales | Seeded Clinical | CIP R, CRO S, CZA S, ETP S, FEP S, GEN R, MEM S, SAM S, TZP R |
| SPBC-3043 | Citrobacter koseri | Enterobacterales | Seeded Clinical | CIP S, CRO S, CZA S, ETP S, FEP R, GEN R, MEM S, SAM S, TZP S |
| SPBC-3044 | Citrobacter koseri | Enterobacterales | Seeded Clinical | CIP S, CRO S, CZA S, ETP S, FEP R, GEN R, MEM S, SAM S, TZP S |
| SPBC-2109 | Citrobacter koseri | Enterobacterales | Seeded Clinical | CIP S, CRO S, CZA S, ETP R, FEP S, GEN R, MEM S, SAM S, TZP R |
| SPBC-3078 | Citrobacter koseri | Enterobacterales | Seeded Clinical | CIP S, CRO S, CZA S, ETP S, FEP R, GEN R, MEM S, SAM S, TZP S |
| SPBC-2145 | Citrobacter koseri | Enterobacterales | Seeded Clinical | CIP S, CRO S, CZA S, ETP S, FEP R, GEN R, MEM S, SAM S, TZP S |
| SPBC-2147 | Citrobacter koseri | Enterobacterales | Seeded Clinical | CIP S, CRO S, CZA S, ETP S, FEP R, GEN R, MEM S, SAM S, TZP S |
| SPBC-1024 | Citrobacter koseri | Enterobacterales | Seeded Clinical | CIP S, CRO S, CZA S, ETP S, FEP R, GEN R, MEM S, SAM S, TZP S |
| SPBC-2148 | Citrobacter koseri | Enterobacterales | Seeded Clinical | CIP S, CRO S, CZA S, ETP S, FEP R, GEN R, MEM S, SAM S, TZP R |
| SPBC-2166 | Citrobacter koseri | Enterobacterales | Seeded Clinical | CIP S, CRO S, CZA S, ETP S, FEP R, GEN R, MEM S, SAM S, TZP R |
| SPBC-2172 | Citrobacter koseri | Enterobacterales | Seeded Clinical | CIP S, CRO S, CZA S, ETP S, FEP R, GEN R, MEM S, SAM S, TZP S |
| SPBC-3094 | Citrobacter koseri | Enterobacterales | Seeded Clinical | CIP S, CRO S, CZA S, ETP S, FEP R, GEN R, MEM S, SAM S, TZP S |
| SPBC-2173 | Citrobacter koseri | Enterobacterales | Seeded Clinical | CIP S, CRO S, CZA S, ETP S, FEP R, GEN R, MEM S, SAM S, TZP R |
| PBC-4122 | Citrobacter koseri | Enterobacterales | Fresh Clinical | CIP S, CRO S, CZA S, ETP S, FEP R, GEN R, MEM S, SAM S, TZP S |
| SPBC-3012 | Enterobacter cloacae complex | Enterobacterales | Seeded Clinical | CIP S, CRO S, CZA S, ETP S, FEP R, GEN R, MEM S |
| SPBC-2026 | Enterobacter cloacae complex | Enterobacterales | Seeded Clinical | CIP S, CRO S, CZA S, ETP S, FEP R, GEN R, MEM S |
| SPBC-1025 | Enterobacter cloacae complex | Enterobacterales | Seeded Clinical | CIP S, CRO R, CZA S, ETP I, FEP SDD, GEN R, MEM S |
| SPBC-3013 | Enterobacter cloacae complex | Enterobacterales | Seeded Clinical | CIP S, CRO R, CZA S, ETP I, FEP S, GEN R, MEM S |
| SPBC-3014 | Enterobacter cloacae complex | Enterobacterales | Seeded Clinical | CIP S, CRO S, CZA S, ETP S, FEP R, GEN R, MEM S |
| SPBC-1027 | Enterobacter cloacae complex | Enterobacterales | Seeded Clinical | CIP S, CRO S, CZA S, ETP S, FEP S, GEN R, MEM S |
| SPBC-3025 | Enterobacter cloacae complex | Enterobacterales | Seeded Clinical | CIP S, CRO R, CZA S, ETP R, FEP R, GEN R, MEM S |
| SPBC-2067 | Enterobacter cloacae complex | Enterobacterales | Seeded Clinical | CIP S, CRO S, CZA S, ETP S, FEP R, GEN R, MEM S |
| SPBC-2068 | Enterobacter cloacae complex | Enterobacterales | Seeded Clinical | CIP S, CRO S, CZA S, ETP S, FEP R, GEN R, MEM S |
| SPBC-3030 | Enterobacter cloacae complex | Enterobacterales | Seeded Clinical | CIP S, CZA S, ETP S, FEP R, GEN R, MEM S |
| SPBC-3038 | Enterobacter cloacae complex | Enterobacterales | Seeded Clinical | CIP R, CRO R, CZA S, FEP R, GEN R, MEM S |
| SPBC-3039 | Enterobacter cloacae complex | Enterobacterales | Seeded Clinical | CIP S, CZA S, ETP S, FEP R, GEN R, MEM S |
| SPBC-2103 | Enterobacter cloacae complex | Enterobacterales | Seeded Clinical | CIP R, CRO R, CZA S, ETP S, FEP R, GEN R, MEM S |
| SPBC-1028 | Enterobacter cloacae complex | Enterobacterales | Seeded Clinical | CIP S, CRO S, CZA S, ETP S, FEP S, GEN R, MEM S |
| SPBC-2118 | Enterobacter cloacae complex | Enterobacterales | Seeded Clinical | CIP S, CRO S, CZA S, ETP S, FEP S, GEN R, MEM S |
| SPBC-2119 | Enterobacter cloacae complex | Enterobacterales | Seeded Clinical | CIP S, CRO S, CZA S, ETP S, FEP R, GEN R, MEM S |
| SPBC-2121 | Enterobacter cloacae complex | Enterobacterales | Seeded Clinical | CIP S, CRO S, CZA S, ETP S, FEP R, GEN R, MEM S |
| SPBC-3070 | Enterobacter cloacae complex | Enterobacterales | Seeded Clinical | CIP S, CRO S, CZA S, ETP S, FEP R, GEN R, MEM S |
| SPBC-2137 | Enterobacter cloacae complex | Enterobacterales | Seeded Clinical | CIP S, CZA S, ETP S, FEP R, GEN R, MEM S |
| SPBC-2138 | Enterobacter cloacae complex | Enterobacterales | Seeded Clinical | CIP S, CRO R, CZA S, ETP S, FEP R, GEN R, MEM S |
| SPBC-3071 | Enterobacter cloacae complex | Enterobacterales | Seeded Clinical | CIP S, CRO S, CZA S, ETP S, FEP R, GEN R, MEM S |
| SPBC-1026 | Enterobacter cloacae complex | Enterobacterales | Seeded Clinical | CIP S, CRO R, CZA S, ETP I, FEP SDD, GEN S, MEM S |
| SPBC-3072 | Enterobacter cloacae complex | Enterobacterales | Seeded Clinical | CIP S, CRO R, CZA S, ETP I, FEP S, GEN R, MEM S |
| SPBC-2139 | Enterobacter cloacae complex | Enterobacterales | Seeded Clinical | CIP S, CRO S, CZA S, ETP S, FEP R, GEN R, MEM S |
| SPBC-3073 | Enterobacter cloacae complex | Enterobacterales | Seeded Clinical | CIP S, CRO S, CZA S, ETP S, FEP R, GEN R, MEM S |
| PBC-1123 | Enterobacter cloacae complex | Enterobacterales | Fresh Clinical | CIP S, CRO R, CZA S, ETP R, FEP S, GEN R, MEM S |
| PBC-1145 | Enterobacter cloacae complex | Enterobacterales | Fresh Clinical | CIP S, CRO S, CZA S, ETP S, FEP S, GEN R, MEM S |
| PBC-2115 | Enterobacter cloacae complex | Enterobacterales | Fresh Clinical | CIP S, CRO S, CZA S, ETP S, FEP R, GEN R, MEM S |
| PBC-4128 | Enterobacter cloacae complex | Enterobacterales | Fresh Clinical | CIP S, CRO S, CZA S, ETP S, FEP R, GEN R, MEM S |
| SD-1156 | Enterobacter cloacae complex | Enterobacterales | Challenge | CIP S, CRO R, CZA S, ETP R, FEP SDD, GEN R, MEM S |
| SD-1158 | Enterobacter cloacae complex | Enterobacterales | Challenge | CIP S, CRO S, CZA S, ETP R, FEP R, GEN R, MEM I |
| SD-2364 | Enterobacter cloacae complex | Enterobacterales | Challenge | CIP S, CRO S, CZA S, ETP S, FEP S, GEN R, MEM S |
| SPBC-2005 | Escherichia coli | Enterobacterales | Seeded Clinical | AMC S, CIP R, GEN R, SAM S |
| SPBC-2021 | Klebsiella aerogenes | Enterobacterales | Seeded Clinical | CIP R, CRO S, CZA S, ETP R, FEP S, GEN R |
| SPBC-2022 | Klebsiella aerogenes | Enterobacterales | Seeded Clinical | CIP S, CRO S, CZA S, ETP S, FEP S, GEN R |
| SPBC-3011 | Klebsiella aerogenes | Enterobacterales | Seeded Clinical | CIP R, CRO S, CZA R, ETP S, FEP S, GEN R |
| SPBC-2024 | Klebsiella aerogenes | Enterobacterales | Seeded Clinical | CIP R, CRO R, CZA S, ETP R, FEP S, GEN R |
| SPBC-2023 | Klebsiella aerogenes | Enterobacterales | Seeded Clinical | CIP R, CRO S, CZA S, ETP S, FEP S, GEN R |
| SPBC-2025 | Klebsiella aerogenes | Enterobacterales | Seeded Clinical | CIP R, CRO S, CZA S, ETP S, FEP S, GEN R |
| SPBC-2080 | Klebsiella aerogenes | Enterobacterales | Seeded Clinical | CIP R, CRO S, CZA S, ETP S, FEP S, GEN R |
| SPBC-2135 | Klebsiella aerogenes | Enterobacterales | Seeded Clinical | CIP R, CRO S, CZA S, ETP S, FEP S, GEN R |
| SPBC-1029 | Klebsiella aerogenes | Enterobacterales | Seeded Clinical | CIP R, CRO S, CZA S, ETP S, FEP R, GEN R |
| SPBC-2136 | Klebsiella aerogenes | Enterobacterales | Seeded Clinical | CIP R, CRO R, CZA S, ETP S, FEP R, GEN R |
| SPBC-3037 | Klebsiella aerogenes | Enterobacterales | Seeded Clinical | CIP R, CRO S, CZA S, ETP S, FEP S, GEN R |
| SPBC-2099 | Klebsiella aerogenes | Enterobacterales | Seeded Clinical | CIP S, CRO S, CZA R, ETP R, FEP S, GEN R |
| SPBC-2100 | Klebsiella aerogenes | Enterobacterales | Seeded Clinical | CIP S, CRO S, CZA S, ETP R, FEP S, GEN R |
| SPBC-3068 | Klebsiella aerogenes | Enterobacterales | Seeded Clinical | CIP R, CRO S, CZA R, ETP R, FEP S, GEN R |
| SPBC-1030 | Klebsiella aerogenes | Enterobacterales | Seeded Clinical | CIP R, CRO S, CZA S, ETP S, FEP S, GEN R |
| SPBC-3069 | Klebsiella aerogenes | Enterobacterales | Seeded Clinical | CIP R, CRO S, CZA S, ETP S, FEP S, GEN R |
| PBC-1139 | Klebsiella aerogenes | Enterobacterales | Fresh Clinical | CIP S, CRO S, CZA S, ETP S, FEP S, GEN R |
| PBC-1148 | Klebsiella aerogenes | Enterobacterales | Fresh Clinical | CIP S, CRO S, CZA R, ETP R, FEP S, GEN R |
| PBC-2122 | Klebsiella aerogenes | Enterobacterales | Fresh Clinical | CIP S, CRO S, CZA S, ETP R, FEP S, GEN R |
| PBC-4120 | Klebsiella aerogenes | Enterobacterales | Fresh Clinical | CIP R, CRO S, CZA S, ETP R, FEP S, GEN R |
| SPBC-2008 | Klebsiella pneumoniae | Enterobacterales | Seeded Clinical | AMC R, CIP R, GEN R, SAM R |
| SPBC-2010 | Klebsiella pneumoniae | Enterobacterales | Seeded Clinical | AMC R, CIP R, GEN S, SAM R |
| SPBC-2012 | Klebsiella pneumoniae | Enterobacterales | Seeded Clinical | AMC I, CIP R, GEN R, SAM R |
| SPBC-2051 | Morganella morganii | Enterobacterales | Seeded Clinical | CIP R, CZA S, ETP R, FEP S, GEN R, MEM R, SAM I, TZP R |
| SPBC-2053 | Morganella morganii | Enterobacterales | Seeded Clinical | CIP R, CZA S, ETP R, FEP R, GEN R, MEM R, SAM R, TZP R |
| SPBC-2054 | Morganella morganii | Enterobacterales | Seeded Clinical | CIP R, CZA S, ETP R, FEP R, GEN R, MEM R, SAM R, TZP R |
| SPBC-3019 | Morganella morganii | Enterobacterales | Seeded Clinical | CIP R, CZA S, ETP R, FEP R, GEN R, MEM S, SAM R, TZP R |
| SPBC-2072 | Morganella morganii | Enterobacterales | Seeded Clinical | CIP R, CZA S, ETP R, FEP R, GEN R, MEM S, SAM R, TZP R |
| SPBC-2052 | Morganella morganii | Enterobacterales | Seeded Clinical | CIP R, CZA S, ETP R, FEP R, GEN R, MEM S, SAM R, TZP R |
| SPBC-2074 | Morganella morganii | Enterobacterales | Seeded Clinical | CIP R, CZA S, ETP S, FEP S, GEN R, MEM S, SAM R, TZP R |
| SPBC-2082 | Morganella morganii | Enterobacterales | Seeded Clinical | CIP R, CZA S, ETP R, FEP R, GEN R, MEM R, SAM R, TZP R |
| SPBC-3020 | Morganella morganii | Enterobacterales | Seeded Clinical | CIP R, CZA S, ETP R, FEP R, GEN R, MEM S, SAM R, TZP R |
| SPBC-2055 | Morganella morganii | Enterobacterales | Seeded Clinical | CIP R, CZA S, ETP R, FEP R, GEN R, MEM R, SAM I |
| SPBC-2120 | Morganella morganii | Enterobacterales | Seeded Clinical | CIP R, CZA S, ETP R, FEP R, GEN R, MEM R, SAM I, TZP R |
| SPBC-3027 | Morganella morganii | Enterobacterales | Seeded Clinical | CIP R, CZA S, ETP R, FEP R, GEN R, MEM S, SAM I, TZP R |
| SPBC-2073 | Morganella morganii | Enterobacterales | Seeded Clinical | CIP R, CZA S, ETP R, FEP R, GEN S, MEM S, SAM R, TZP R |
| SPBC-2079 | Morganella morganii | Enterobacterales | Seeded Clinical | CIP S, CZA S, ETP R, FEP R, GEN R, MEM S, SAM R, TZP R |
| SPBC-2104 | Morganella morganii | Enterobacterales | Seeded Clinical | CIP R, CZA S, ETP R, FEP S, GEN R, MEM R, SAM R, TZP R |
| SPBC-3040 | Morganella morganii | Enterobacterales | Seeded Clinical | CIP R, CZA S, ETP R, FEP S, GEN R, MEM S, SAM I, TZP R |
| SPBC-2116 | Morganella morganii | Enterobacterales | Seeded Clinical | CIP R, CZA S, ETP S, FEP S, GEN R, MEM S, SAM R, TZP R |
| SPBC-2154 | Morganella morganii | Enterobacterales | Seeded Clinical | CIP R, CZA S, ETP S, FEP SDD, GEN R, MEM S, SAM R, TZP R |
| SPBC-2155 | Morganella morganii | Enterobacterales | Seeded Clinical | CIP R, CZA S, ETP R, FEP R, GEN R, MEM R, SAM R, TZP R |
| SPBC-2156 | Morganella morganii | Enterobacterales | Seeded Clinical | CIP R, CZA S, ETP R, FEP R, GEN R, MEM R, SAM R, TZP R |
| PBC-4108 | Morganella morganii | Enterobacterales | Fresh Clinical | CIP R, CZA S, ETP R, FEP R, GEN S, MEM S, SAM R, TZP R |
| SPBC-2114 | Proteus vulgaris | Enterobacterales | Seeded Clinical | CIP S, CZA S, ETP S, FEP S, GEN R, MEM S, TZP R |
| SPBC-1039 | Serratia marcescens | Enterobacterales | Seeded Clinical | CIP S, CRO S, CZA S, ETP S, FEP R, GEN R, MEM R, TZP R |
| SPBC-2027 | Serratia marcescens | Enterobacterales | Seeded Clinical | CIP R, CRO S, CZA S, ETP S, FEP S, GEN R, MEM S, TZP R |
| SPBC-2028 | Serratia marcescens | Enterobacterales | Seeded Clinical | CIP S, CRO S, CZA S, ETP S, FEP R, GEN R, MEM S, TZP R |
| SPBC-2029 | Serratia marcescens | Enterobacterales | Seeded Clinical | CIP I, CRO R, CZA S, ETP R, FEP SDD, GEN R, TZP R |
| SPBC-2030 | Serratia marcescens | Enterobacterales | Seeded Clinical | CIP S, CRO S, CZA S, ETP S, FEP R, GEN R, MEM R, TZP S |
| SPBC-2031 | Serratia marcescens | Enterobacterales | Seeded Clinical | CIP I, CRO S, CZA S, ETP R, FEP S, GEN R, MEM R, TZP R |
| SPBC-2106 | Serratia marcescens | Enterobacterales | Seeded Clinical | CIP S, CRO S, CZA S, ETP S, FEP R, GEN R, MEM S, TZP R |
| SPBC-1040 | Serratia marcescens | Enterobacterales | Seeded Clinical | CIP S, CRO S, CZA S, ETP S, FEP S, GEN R, MEM S, TZP R |
| SPBC-2107 | Serratia marcescens | Enterobacterales | Seeded Clinical | CIP S, CRO S, CZA S, ETP S, FEP R, GEN R, MEM S, TZP S |
| SPBC-2108 | Serratia marcescens | Enterobacterales | Seeded Clinical | CIP S, CRO S, CZA S, ETP S, FEP S, GEN R, MEM S, TZP R |
| SPBC-3074 | Serratia marcescens | Enterobacterales | Seeded Clinical | CIP S, CRO S, CZA S, FEP R, GEN R, MEM S, TZP S |
| SPBC-2140 | Serratia marcescens | Enterobacterales | Seeded Clinical | CIP S, CRO S, CZA S, ETP S, FEP R, GEN R, MEM S, TZP R |
| SPBC-3075 | Serratia marcescens | Enterobacterales | Seeded Clinical | CIP S, CRO S, CZA S, ETP S, FEP R, GEN S, MEM R, TZP S |
| SPBC-2141 | Serratia marcescens | Enterobacterales | Seeded Clinical | CIP S, CRO S, CZA S, ETP S, FEP S, GEN R, MEM S, TZP R |
| SPBC-2142 | Serratia marcescens | Enterobacterales | Seeded Clinical | CIP S, CRO S, CZA S, ETP S, FEP S, GEN R, MEM R, TZP R |
| SPBC-2167 | Serratia marcescens | Enterobacterales | Seeded Clinical | CIP S, CRO S, CZA S, ETP S, FEP S, GEN R, MEM S, TZP R |
| SPBC-3092 | Serratia marcescens | Enterobacterales | Seeded Clinical | CIP S, CRO S, CZA S, ETP S, FEP R, GEN S, MEM R, TZP S |
| SPBC-2168 | Serratia marcescens | Enterobacterales | Seeded Clinical | CIP S, CRO S, CZA S, FEP S, GEN R, MEM S, TZP S |
| SPBC-2169 | Serratia marcescens | Enterobacterales | Seeded Clinical | CIP S, CRO S, CZA S, ETP R, FEP R, GEN R, MEM R, TZP R |
| SPBC-2170 | Serratia marcescens | Enterobacterales | Seeded Clinical | CIP S, CRO S, CZA S, ETP S, FEP R, GEN R, MEM S, TZP R |
| SPBC-2171 | Serratia marcescens | Enterobacterales | Seeded Clinical | CIP I, CRO S, CZA S, ETP R, FEP R, GEN S, MEM S, TZP R |
| SPBC-1038 | Serratia marcescens | Enterobacterales | Seeded Clinical | CIP S, CRO S, CZA S, ETP R, FEP R, GEN R, MEM S, TZP R |
| PBC-1159 | Serratia marcescens | Enterobacterales | Fresh Clinical | CIP S, CRO S, CZA S, ETP S, FEP R, GEN R, MEM S, TZP R |
| PBC-4140 | Enterobacter cloacae complex | Enterobacterales | Fresh Clinical | CZA S, ETP R, FEP R |
| SD-1441 | Pseudomonas aeruginosa | Pseudomonas aeruginosa | Challenge | AMK S, CAZ S, CIP R, CZA S, FEP S, GEN R, MEM I, TOB R, TZP S |
| SD-1446 | Pseudomonas aeruginosa | Pseudomonas aeruginosa | Challenge | AMK S, CAZ S, CIP S, CZA S, FEP S, GEN S, MEM S, TOB S, TZP S |
| SD-1476 | Pseudomonas aeruginosa | Pseudomonas aeruginosa | Challenge | AMK S, CAZ R, CIP R, CZA R, FEP R, GEN S, MEM R, TOB I, TZP I |
| SD-1478 | Pseudomonas aeruginosa | Pseudomonas aeruginosa | Challenge | AMK S, CAZ R, CIP R, CZA S, FEP R, GEN S, MEM R, TOB S, TZP R |
| SD-1483 | Pseudomonas aeruginosa | Pseudomonas aeruginosa | Challenge | AMK S, CAZ S, CIP S, CZA S, FEP S, GEN S, MEM S, TOB S, TZP S |
| SD-1489 | Pseudomonas aeruginosa | Pseudomonas aeruginosa | Challenge | AMK S, CAZ S, CIP R, CZA S, FEP S, GEN R, MEM I, TOB R, TZP S |
| PBC-3115 | Acinetobacter baumannii complex | Acinetobacter baumannii | Fresh Clinical | IMP R, MEM R, MIN R, SAM R, TZP R |
| PBC-1120 | Acinetobacter baumannii complex | Acinetobacter baumannii | Fresh Clinical | IMP R, MEM R, MIN R, SAM R, TZP R |

**Supplementary Table 12: Results of Clinical Performance Evaluation for the PBC Separator and Selux AST System: Fresh PBC samples only**

| **Antimicrobial Agent** | **Organism Group** | **Total Tested** | **# in EA** | **% EA** | **Total Eval** | **# Eval in EA** | **% EA of Eval** | **# in CA** | **% CA** | **# R** | **# VMJ** | **# MAJ** | **# MIN** |
| --- | --- | --- | --- | --- | --- | --- | --- | --- | --- | --- | --- | --- | --- |
| Amikican | *A. baumannii complex* | 1 | 1 | 100 | 0 | 0 | N/A | 1 | 100 | 1 | 0 | 0 | 0 |
|  | Enterobacterales | 98 | 94 | 95.9 | 11 | 7 | 63.6 | 98 | 100 | 0 | 0 | 0 | 0 |
|  | *P. aeruginosa* | 13 | 13 | 100 | 13 | 13 | 100 | 13 | 100 | 0 | 0 | 0 | 0 |
| Amoxicillin-clavulanate | Enterobacterales | 134 | 134 | 100 | 97 | 97 | 100 | 120 | 89.6 | 2 | 0 | 0 | 14 |
| Ampicillin | Enterobacterales | 74 | 73 | 98.6 | 2 | 1 | 50 | 73 | 98.6 | 49 | 0 | 0 | 1 |
| Ampicillin-sulbactam | *A. baumannii complex* | 3 | 1 | 33.3 | 2 | 0 | 0 | 3 | 100 | 3 | 0 | 0 | 0 |
|  | Enterobacterales | 136 | 135 | 99.3 | 131 | 130 | 99.2 | 115 | 84.6 | 26 | 0 | 0 | 21 |
| Cefazolin | Enterobacterales | 95 | 90 | 94.7 | 70 | 65 | 92.9 | 84 | 88.4 | 32 | 0 | 0 | 11 |
| Cefepime | Enterobacterales | 118 | 113 | 95.8 | 10 | 5 | 50 | 113 | 95.8 | 16 | 0 | 0 | 5 |
|  | *P. aeruginosa* | 12 | 12 | 100 | 12 | 12 | 100 | 12 | 100 | 1 | 0 | 0 | 0 |
| Ceftazidime | Enterobacterales | 98 | 96 | 98 | 30 | 28 | 93.3 | 91 | 92.9 | 20 | 0 | 0 | 7 |
|  | *P. aeruginosa* | 10 | 10 | 100 | 10 | 10 | 100 | 10 | 100 | 0 | 0 | 0 | 0 |
| Ceftazidime-avibactam | Enterobacterales | 118 | 116 | 98.3 | 9 | 7 | 77.8 | 118 | 100 | 0 | 0 | 0 | 0 |
|  | *P. aeruginosa* | 12 | 12 | 100 | 12 | 12 | 100 | 12 | 100 | 0 | 0 | 0 | 0 |
| Ceftriaxone | Enterobacterales | 116 | 115 | 99.1 | 1 | 0 | 0 | 115 | 99.1 | 26 | 0 | 1 | 0 |
| Ciprofloxacin | Enterobacterales | 145 | 141 | 97.2 | 25 | 21 | 84 | 140 | 96.6 | 36 | 0 | 1 | 4 |
|  | *P. aeruginosa* | 12 | 12 | 100 | 10 | 10 | 100 | 11 | 91.7 | 1 | 0 | 0 | 1 |
| Ertapenem | Enterobacterales | 117 | 116 | 99.1 | 12 | 11 | 91.7 | 116 | 99.1 | 1 | 0 | 0 | 1 |
| Gentamicin | Enterobacterales | 143 | 141 | 98.6 | 10 | 8 | 80 | 141 | 98.6 | 20 | 0 | 1 | 1 |
|  | *P. aeruginosa* | 13 | 13 | 100 | 11 | 11 | 100 | 13 | 100 | 0 | 0 | 0 | 0 |
| Imipenem | *A. baumannii complex* | 3 | 3 | 100 | 0 | 0 | N/A | 3 | 100 | 3 | 0 | 0 | 0 |
|  | Enterobacterales | 96 | 93 | 96.9 | 4 | 1 | 25 | 94 | 97.9 | 0 | 0 | 1 | 1 |
| Meropenem | *A. baumannii complex* | 3 | 3 | 100 | 0 | 0 | N/A | 3 | 100 | 3 | 0 | 0 | 0 |
|  | Enterobacterales | 113 | 112 | 99.1 | 2 | 1 | 50 | 112 | 99.1 | 0 | 0 | 0 | 1 |
|  | *P. aeruginosa* | 12 | 12 | 100 | 6 | 6 | 100 | 12 | 100 | 1 | 0 | 0 | 0 |
| Minocycline | *A. baumannii complex* | 3 | 3 | 100 | 3 | 3 | 100 | 3 | 100 | 3 | 0 | 0 | 0 |
|  | Enterobacterales | 98 | 93 | 94.9 | 93 | 88 | 94.6 | 89 | 90.8 | 11 | 0 | 1 | 8 |
| Piperacillin-tazobactam | *A. baumannii complex* | 3 | 3 | 100 | 0 | 0 | N/A | 3 | 100 | 3 | 0 | 0 | 0 |
|  | Enterobacterales | 104 | 101 | 97.1 | 13 | 10 | 76.9 | 100 | 96.2 | 1 | 0 | 0 | 4 |
|  | *P. aeruginosa* | 12 | 11 | 91.7 | 12 | 11 | 91.7 | 11 | 91.7 | 1 | 0 | 0 | 1 |
| Tobramycin | Enterobacterales | 98 | 92 | 93.9 | 96 | 90 | 93.8 | 91 | 92.9 | 12 | 0 | 0 | 7 |
|  | *P. aeruginosa* | 12 | 11 | 91.7 | 12 | 11 | 91.7 | 12 | 100 | 1 | 0 | 0 | 0 |

**Supplementary Table 13: Results of Clinical Performance Evaluation for the PBC Separator and Selux AST System: Contrived PBC samples only**

| **Antimicrobial Agent** | **Organism Group** | **Total Tested** | **# EA** | **% EA** | **Total Eval** | **# Eval in EA** | **% EA of Eval** | **# CA** | **% CA** | **# R** | **# VMJ** | **# MAJ** | **# MIN** |
| --- | --- | --- | --- | --- | --- | --- | --- | --- | --- | --- | --- | --- | --- |
| Amikacin | *A. baumannii complex* | 37 | 34 | 91.9 | 23 | 20 | 87 | 35 | 94.6 | 16 | 0 | 0 | 2 |
|  | Enterobacterales | 118 | 114 | 96.6 | 13 | 9 | 69.2 | 115 | 97.5 | 8 | 0 | 0 | 3 |
|  | *P. aeruginosa* | 31 | 29 | 93.5 | 30 | 28 | 93.3 | 27 | 87.1 | 6 | 0 | 0 | 4 |
| Amoxicillin-clavulanate | Enterobacterales | 196 | 194 | 99 | 125 | 123 | 98.4 | 175 | 89.3 | 37 | 0 | 0 | 21 |
| Ampicillin | Enterobacterales | 75 | 75 | 100 | 2 | 2 | 100 | 75 | 100 | 47 | 0 | 0 | 0 |
| Ampicillin-sulbactam | *A. baumannii complex* | 37 | 36 | 97.3 | 19 | 18 | 94.7 | 35 | 94.6 | 22 | 0 | 0 | 2 |
|  | Enterobacterales | 216 | 212 | 98.1 | 172 | 168 | 97.7 | 191 | 88.4 | 109 | 0 | 0 | 25 |
| Cefazolin | Enterobacterales | 112 | 107 | 95.5 | 48 | 43 | 89.6 | 103 | 92 | 67 | 1 | 0 | 8 |
| Cefepime | Enterobacterales | 288 | 283 | 98.3 | 25 | 20 | 80 | 277 | 96.2 | 45 | 0 | 0 | 11 |
|  | *P. aeruginosa* | 31 | 30 | 96.8 | 25 | 24 | 96 | 30 | 96.8 | 10 | 0 | 0 | 0 |
| Ceftazidime | Enterobacterales | 119 | 119 | 100 | 36 | 36 | 100 | 113 | 95 | 50 | 0 | 0 | 6 |
|  | *P. aeruginosa* | 30 | 30 | 100 | 25 | 25 | 100 | 30 | 100 | 9 | 0 | 0 | 0 |
| Ceftazidime-avibactam | Enterobacterales | 300 | 293 | 97.7 | 87 | 80 | 92 | 300 | 100 | 4 | 0 | 0 | 0 |
|  | *P. aeruginosa* | 31 | 31 | 100 | 27 | 27 | 100 | 30 | 96.8 | 9 | 0 | 0 | 0 |
| Ceftriaxone | Enterobacterales | 257 | 255 | 99.2 | 17 | 15 | 88.2 | 255 | 99.2 | 85 | 0 | 0 | 2 |
| Ciprofloxacin | Enterobacterales | 324 | 320 | 98.8 | 57 | 53 | 93 | 317 | 97.8 | 81 | 0 | 0 | 7 |
|  | *P. aeruginosa* | 31 | 31 | 100 | 22 | 22 | 100 | 31 | 100 | 12 | 0 | 0 | 0 |
| Ertapenem | Enterobacterales | 295 | 289 | 98 | 50 | 44 | 88 | 292 | 99 | 27 | 0 | 0 | 3 |
| Gentamicin | Enterobacterales | 323 | 318 | 98.5 | 23 | 18 | 78.3 | 318 | 98.5 | 44 | 0 | 0 | 5 |
|  | *P. aeruginosa* | 30 | 29 | 96.7 | 20 | 19 | 95 | 29 | 96.7 | 8 | 0 | 0 | 1 |
| Imipenem | *A. baumannii complex* | 36 | 35 | 97.2 | 4 | 3 | 75 | 36 | 100 | 23 | 0 | 0 | 0 |
|  | Enterobacterales | 117 | 112 | 95.7 | 10 | 5 | 50 | 115 | 98.3 | 20 | 0 | 0 | 2 |
| Meropenem | *A. baumannii complex* | 36 | 34 | 94.4 | 14 | 12 | 85.7 | 36 | 100 | 24 | 0 | 0 | 0 |
|  | Enterobacterales | 281 | 276 | 98.2 | 17 | 12 | 70.6 | 278 | 98.9 | 19 | 0 | 0 | 3 |
|  | *P. aeruginosa* | 31 | 28 | 90.3 | 22 | 19 | 86.4 | 27 | 87.1 | 12 | 0 | 0 | 4 |
| Minocycline | *A. baumannii complex* | 36 | 35 | 97.2 | 21 | 20 | 95.2 | 30 | 83.3 | 9 | 0 | 0 | 6 |
|  | Enterobacterales | 120 | 116 | 96.7 | 100 | 96 | 96 | 106 | 88.3 | 21 | 0 | 1 | 13 |
| Piperacillin-tazobactam | *A. baumannii complex* | 36 | 34 | 94.4 | 5 | 3 | 60 | 35 | 97.2 | 24 | 0 | 0 | 1 |
|  | Enterobacterales | 216 | 212 | 98.1 | 23 | 19 | 82.6 | 212 | 98.1 | 36 | 0 | 1 | 3 |
|  | *P. aeruginosa* | 31 | 31 | 100 | 28 | 28 | 100 | 31 | 100 | 8 | 0 | 0 | 0 |
| Tobramycin | Enterobacterales | 118 | 115 | 97.5 | 104 | 101 | 97.1 | 109 | 92.4 | 37 | 0 | 0 | 9 |
|  | *P. aeruginosa* | 31 | 30 | 96.8 | 25 | 24 | 96 | 29 | 93.5 | 9 | 0 | 0 | 2 |

**Supplementary Table 14: Results of Performance Evaluation of Antimicrobial-Organism Combinations with CLSI Breakpoints Differing from FDA Breakpoints**

| **Antimicrobial Agent** | **Organism Group** | **CLSI Breakpoints (S/I/R)** | **Total Tested** | **# EA** | **% EA** | **# CA** | **% CA** | **# R** | **# VMJ** | **# MAJ** | **# MIN** |
| --- | --- | --- | --- | --- | --- | --- | --- | --- | --- | --- | --- |
| Amikacin | Enterobacterales | 4/8/16 | 216 | 208 | 96.3 | 204 | 94.4 | 18 | 1 | 0 | 11 |
| Ceftazidime | *P. aeruginosa* | 8/16/32 | 40 | 40 | 100 | 40 | 100 | 9 | 0 | 0 | 0 |
| Cefepime | *P. aeruginosa* | 8/16/32 | 43 | 42 | 97.7 | 42 | 97.7 | 9 | 0 | 0 | 1 |
| Gentamicin | Enterobacterales | 2/4/8 | 466 | 459 | 98.5 | 462 | 99.1 | 68 | 1 | 1 | 2 |
| Tobramycin | Enterobacterales | 2/4/8 | 216 | 207 | 95.8 | 205 | 94.9 | 58 | 0 | 3 | 8 |
|  | *P. aeruginosa* | 1/2/4 | 43 | 41 | 95.3 | 43 | 100 | 12 | 0 | 0 | 0 |
| Piperacillin-tazobactam | *P. aeruginosa* | 16/32/64 | 43 | 42 | 97.7 | 43 | 100 | 10 | 0 | 0 | 0 |
